# Evolutionary investigations of the biosynthetic diversity in the skin microbiome using *lsa*BGC

**DOI:** 10.1099/mgen.0.000988

**Published:** 2023-04-28

**Authors:** Rauf Salamzade, J.Z. Alex Cheong, Shelby Sandstrom, Mary Hannah Swaney, Reed M. Stubbendieck, Nicole Lane Starr, Cameron R. Currie, Anne Marie Singh, Lindsay R. Kalan

**Affiliations:** ^1^​ Department of Medical Microbiology and Immunology, School of Medicine and Public Health, University of Wisconsin, Madison, WI, USA; ^2^​ Microbiology Doctoral Training Program, University of Wisconsin, Madison, WI, USA; ^3^​ Department of Bacteriology, University of Wisconsin, Madison, Wisconsin, USA; ^4^​ M.G. DeGroote Institute for Infectious Disease Research, David Braley Centre for Antibiotic Discovery, Department of Biochemistry and Biomedical Sciences, McMaster University, Hamilton, Ontario, Canada; ^5^​ Department of Pediatrics, School of Medicine and Public Health, University of Wisconsin, Madison, WI, USA; ^6^​ Department of Medicine, Division of Infectious Disease, School of Medicine and Public Health, University of Wisconsin, Madison, WI, USA

**Keywords:** biosynthetic gene clusters, microbial evolution, bioinformatics, skin microbiome, staphyloxanthin, metagenomic mining

## Abstract

Bacterial secondary metabolites, synthesized by enzymes encoded in biosynthetic gene clusters (BGCs), can underlie microbiome homeostasis and serve as commercialized products, which have historically been mined from a select group of taxa. While evolutionary approaches have proven beneficial for prioritizing BGCs for experimental characterization efforts to uncover new natural products, dedicated bioinformatics tools designed for comparative and evolutionary analysis of BGCs within focal taxa are limited. We thus developed lineage specific analysis of BGCs (*lsa*BGC; https://github.com/Kalan-Lab/lsaBGC) to aid exploration of microdiversity and evolutionary trends across homologous groupings of BGCs, gene cluster families (GCFs), in any bacterial taxa of interest. *lsa*BGC enables rapid and direct identification of GCFs in genomes, calculates evolutionary statistics and conservation for BGC genes, and builds a framework to allow for base resolution mining of novel variants through metagenomic exploration. Through application of the suite to four genera commonly found in skin microbiomes, we uncover new insights into the evolution and diversity of their BGCs. We show that the BGC of the virulence-associated carotenoid staphyloxanthin in *Staphylococcus aureus* is ubiquitous across the genus *Staphylococcus *. While one GCF encoding the biosynthesis of staphyloxanthin showcases evidence for plasmid-mediated horizontal gene transfer (HGT) between species, another GCF appears to be transmitted vertically amongst a sub-clade of skin-associated *Staphylococcus *. Further, the latter GCF, which is well conserved in *S. aureus *, has been lost in most *Staphylococcus epidermidis *, which is the most common *Staphylococcus* species on human skin and is also regarded as a commensal. We also identify thousands of novel single-nucleotide variants (SNVs) within BGCs from the *Corynebacterium tuberculostearicum* sp. complex, a narrow, multi-species clade that features the most prevalent *Corynebacterium* in healthy skin microbiomes. Although novel SNVs were approximately 10 times as likely to correspond to synonymous changes when located in the top five percentile of conserved sites, *lsa*BGC identified SNVs that defied this trend and are predicted to underlie amino acid changes within functionally key enzymatic domains. Ultimately, beyond supporting evolutionary investigations of BGCs, *lsa*BGC also provides important functionalities to aid efforts for the discovery or directed modification of natural products.

## Data Summary

Genomic assemblies and sequencing reads for *Staphylococcus* and *Micrococcus luteus* isolates we introduced in this study are provided on the National Center for Biotechnology Information (NCBI) database under BioProjects PRJNA803478 and PRJNA830888. The *lsa*BGC software suite was developed in Python 3 and R and is available on Github at: https://github.com/Kalan-Lab/lsaBGC. Algorithmic descriptions of programs can be found on the Github wiki and in Text S1 (available in the online version of this article). A small test case consisting of *Cutibacterium* BGCs and genomes is included in the Github repository. Version 1.0 of the software was used for the analyses and results described in this study unless otherwise noted. Supplementary materials for the paper can be found on Figshare at 10.6084/m9.figshare.21689132, including primary results by *lsa*BGC-Easy (v1.31) for *Cutibacterium avidum* using default parameters and antiSMASH (v6.0.0) for BGC identification (Dataset S1). The authors confirm all supporting data, code and protocols have been provided within the article or through supplementary data files [1].

**Impact Statement**

Comprehensive studies of the biosynthetic gene clusters (BGCs) for specific taxa have become commonplace in the last 5 years. Deeper investigations into individual gene cluster families (GCFs), however, remain largely a manual process. Here we introduce *lsa*BGC to simplify such investigations with easy-to-use bioinformatic workflows. The functionalities provided by *lsa*BGC will not only support evolutionary investigations of BGCs but also aid efforts to discover or synthesize new natural products, such as antimicrobials. We applied *lsa*BGC to genera common to the skin microbiome and revealed that the virulence factor staphyloxanthin is not confined to *Staphylococcus aureus* but prevalent across staphylococci. This has important implications for research aimed at targeting this metabolite to quell *S. aureus *. In addition, through application to the *Corynebacterium tuberculostearicum* species complex, we show how *lsa*BGC can identify specific metagenomes harbouring strains with novel and predicted non-synonymous variants within functionally important domains.

## Introduction

The secondary metabolome of bacteria has served as a valuable reservoir of natural products with great societal benefit [2]. Historically, many commercially available drugs, including antibiotics, have been identified from particular taxonomic groups, such as the genus *Streptomyces* within Actinomycetota [2–4]. Several studies have explored the secondary metabolome of these metabolically rich microbial taxa to develop a comprehensive understanding of their chemical diversity and unique traits [5–8]. Most of these studies first identify biosynthetic gene clusters within genomes using the popular antiSMASH software [9] and then group biosynthetic gene clusters (BGCs) into gene cluster families (GCFs) using BiG-SCAPE [10]. This approach uses a set of key protein domains as their base to group BGCs predicted to encode similar metabolites – a method similar to other bioinformatics software for genomics annotation and analysis of BGCs [11–13]. However, when working in the context of a single species or genus, it becomes advantageous to use full protein sequences and identify orthologues [14], which provide greater resolution for determining evolutionary relationships between BGCs. Formal identification of orthologous proteins then permits the calculation of evolutionary statistics for genes across BGC instances belonging to a common GCF. Further, trends around which genes might be the most rapidly evolving or display signatures of vertical vs horizontal descent can be identified.

Due to difficulties in the cultivation of many bacterial taxa, several recent studies have begun to explore metagenomic datasets to unearth novel secondary metabolites [15–17]. While previous endeavours relied on performing metagenomic assembly, two recent read-based approaches were described to reliably and sensitively search for key BGC domains in metagenomes [16, 18]. This highlights a larger trend toward seeking highly novel secondary metabolites and, in turn, prioritized investigation of poorly studied or newly discovered taxa [19]. Nevertheless, there could be tremendous reward for efforts to advance our understanding of catalogued but uncharacterized BGCs from well-studied taxa that may lead to novel biological insights beyond natural products discovery. Recent studies that explored the vertical descent and conservation of BGCs across the genus of *Salinospora* and the fungal species *Aspergillus flavus* have shown that intra-genus or intra-species evolution can result in chemical and regulatory diversification of the encoded metabolites [7, 20]. Moreover, methods to leverage metagenomic datasets and identify homologous instances of rare BGCs from species with limited representative genomes can lead to critical insight into their function and ecological distribution [5].

To aid the advancement of such taxa-specific analyses of BGCs, we introduce the comprehensive software suite lineage specific analysis of BGCs (*lsa*BGC; https://github.com/Kalan-Lab/lsaBGC), which consists of several programs for comparative genetics of BGCs determined directly from antiSMASH, as well as metagenomic mining. The utility of the multiple functionalities included in the *lsa*BGC suite, which expand and complement the existing array of software for evolutionary investigations of BGCs, is described here through application to four major genera commonly found in healthy human skin microbiomes. Several secondary metabolites that function as antibiotics [21–23], virulence factors [24], or in microbe–host interactions [25, 26] have been identified in bacteria from skin microbiomes, primarily from staphylococci. Human skin is also easily accessible for sampling and most species can be cultivated for follow-up studies. Using *lsa*BGC, we reveal new ecological and evolutionary insights for both well-characterized and unknown predicted metabolites, including signatures of inter-species transfer for the virulence-associated staphylococcal carotenoid staphyloxanthin and a highly conserved predicted siderophore found encoded by diverse *Corynebacterium* species. In addition, through mining skin metagenomic datasets, we find novel coding variants within the functionally important ketide synthase (KS) domain of the polyketide synthase (PKS) responsible for mycolic acid biosynthesis, a defining cell wall component of *Corynebacterium* species implicated in recognition by host immune systems.

## Results

### *Isa*BGC allows for sensitive and systematic identification of BGC homologue groups

**Fig. 1.**
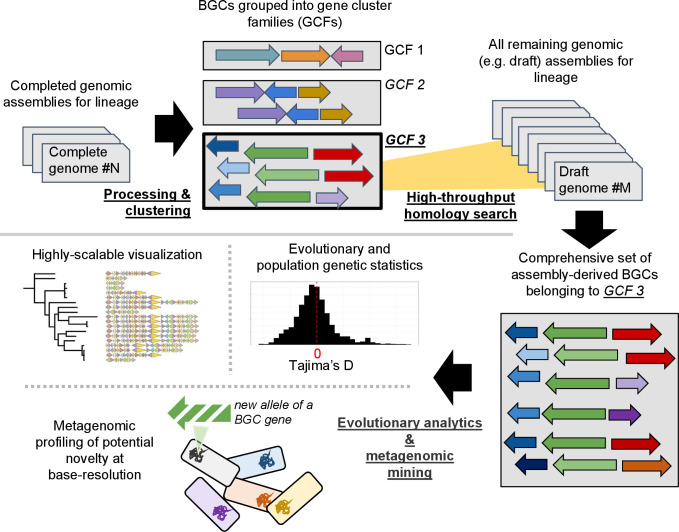
An overview of the *lsa*BGC suite. A schematic overview of the *lsa*BGC suite is shown, highlighting functionalities to: cluster BGCs to GCFs, identify homologous instances of GCFs directly in assemblies, and perform evolutionary and metagenomic investigations.

The *lsa*BGC suite consists of eight core programs, four workflows and several additional scripts for analyses [Figs 1, S1 and S2 and Text S1 (available in the online version of this article)]. These programs allow for clustering BGCs into GCFs, refining boundaries of BGCs, and sensitive, yet specific, rapid detection of GCF instances in draft-quality assemblies that often contain BGCs fragmented across scaffolds. The suite additionally includes dedicated tools for GCF visualization, evolutionary and population genetic analysis of individual genes within GCFs, and base resolution identification of novel single-nucleotide variants (SNVs) in complex metagenomes.

To demonstrate the capabilities of the *lsa*BGC suite we applied it to four genera that are stable and integral members of the human skin microbiome [27–31]. These included three genera in the phylum Actinomycetota (formerly Actinobacteria), *Corynebacterium *, *Cutibacterium* and *Micrococcus *, as well as the Bacillota (formerly Firmicutes) genus *Staphylococcus. lsa*BGC was also independently applied to the species or species complex from each genus most commonly represented in skin microbiomes. These were the *Corynebacterium tuberculostearicum* species complex, *Cutibacterium acnes *, *Micrococcus luteus* and *Staphylococcus epidermidis* (Table S1).

**Fig. 2.**
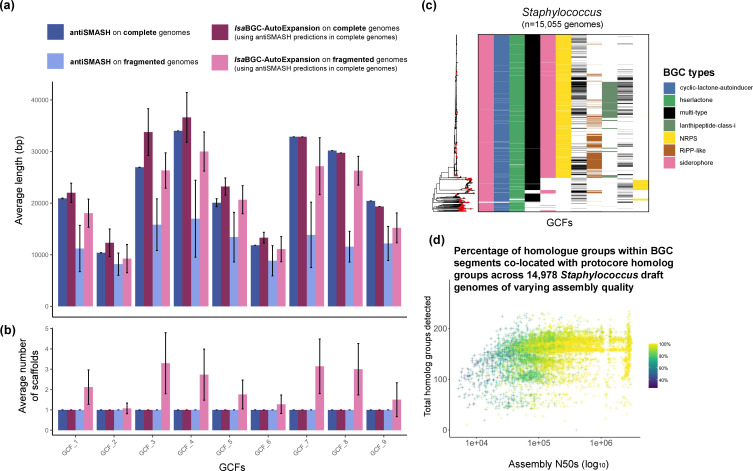
*lsa*BGC offers an efficient and sensitive means for GCF homology detection. (**a**) The aggregate length in base pairs and (**b**) the number of scaffolds that featured a segment regarded as belonging to 1 of 9 GCFs are shown for antiSMASH and *lsa*BGC-AutoExpansion annotation of BGCs when applied to 14 complete *M. luteus* genomes and their fragmented variants generated using read-based simulation and reassembly. *lsa*BGC-AutoExpansion was applied to both complete and fragmented genomes using GCF profiles established from BGCs identified by antiSMASH on complete genomes and clustered with *lsa*BGC-Cluster. Results were averaged across either samples or across samples and replicate fragmented genomes with bars corresponding to a single standard deviation. Asterisks (*) indicate singleton GCFs found in only one genome. (**c**) A neighbour-joining tree of 15 057 *Staphylococcus* genomes based on pairwise MASH ANI estimates is shown alongside a heatmap indicating the presence of GCFs. Only GCFs found in >5 % of genomes are shown. Red dots on neighbour-joining trees signify complete or chromosomal genomes used for initial identification of BGCs. Heatmap cell colour indicates the major BGC class as predicted by antiSMASH. (**d**) The relationship between the assembly quality measured by N50 and the total number of homologue groups detected across 14 978 *Staphylococcus* draft genomes is shown. The colouring corresponds to the percentage of BGC homologue groups co-located in a segment with a homologue group commonly found in protocore regions of BGC predictions by antiSMASH run on completed genomes.

First, *lsa*BGC-AutoProcess was used to run gene calling [32, 33], BGC annotation [9] and *de novo* homologue group delineation [14] with complete or chromosomally complete genomes for each genus or species. Then *lsa*BGC-Cluster was applied to group homologous BGCs into GCFs and systematically searched for instances of GCFs using *lsa*BGC-AutoExpansion (Figs 2, S1c; Tables S2, S3; Text S1). To assess the validity and sensitivity of this approach, two benchmarking tests using BGCs identified in *M. luteus* were performed showing that the *lsa*BGC-AutoExpansion framework for direct identification of GCF instances leads to greater sensitivity compared to running *de novo* antiSMASH on fragmented draft-quality assemblies (Figs 2a, b, S3, S4; Text S1). This increased sensitivity was due to more comprehensive detection of auxiliary genes located on the edges of scaffolds not containing core domains used by antiSMASH for BGC detection. Because antiSMASH delineates BGC boundaries based on physical distance to core domains, we also found that *lsa*BGC-AutoExpansion can synchronize inclusion of genes near BGC boundaries, which are variably included as part of BGCs by antiSMASH across homologous instances from different genomes (Figs 2a, b, S4; Text S1). In addition, *lsa*BGC-AutoExpansion is highly efficient and allowed us to search for 63 GCFs identified from high-quality *Staphylococcus* genomes in 14 978 draft-quality genomes in approximately 14 h using 40 cores and less than 150 Gb of memory (Fig. 2c). A positive association between complete BGC instances and assembly quality was observed (Fig. 2d; Text S1). While the detection of false-positive BGC segments annotated using *lsa*BGC-AutoExpansion is rare (Figs 1c, d, S4) due to BGC conservation within species and genera [34, 35], users are still advised to visually inspect such segments using *lsa*BGC-See figures and filter segments, if necessary.

### *lsa*BGC enables reliable comparative genomics to explore evolutionary trends of BGCs

The increased sensitivity of *lsa*BGC to uniformly detect auxiliary components of BGCs in each genome enabled us to perform reliable comparative genetic analyses across GCF instances. We first focused on two of the four genera with the greatest breadth of diversity, *Corynebacterium* and *Staphylococcus *, and selected a subset of 456 and 229 dereplicated, representative genomes from each genus, respectively (Table S1). To better understand which species in these genera were most relevant to the skin environment, we first profiled how often distinct species were found among healthy skin microbiomes using a large, recently generated metagenomics dataset [36, 37] (Table S4; Text S1). Skin-associated species (found in more than 10 of 270 metagenomes) were phylogenetically clustered for both genera, within the *S. epidermidis/aureus* clade (Fig. S5a) and the *C. tuberculostearicum* species complex (Fig. S5b).

Examining the association of individual homologue groups to skin-associated species revealed that several GCFs were enriched within skin-associated staphylococci, but only two were enriched for skin-associated corynebacteria, both encoding hypothetical proteins (Table S5; Text S1). Nearly half of the homologue groups enriched for skin-associated staphylococci (5 of 12; 41.7%) were related to GCF-10, predicted to encode a non-ribosomal peptide synthetase (NRPS) corresponding to pyrazinone biosynthesis. Pyrazinones are reported to regulate virulence in *S. aureus* [24, 38] and were previously identified as being synthesized by two enzymes, PznA and PznB, specific to species commonly associated with human skin, including *S. aureus* and *S. epidermidis* [24, 38]. Through examination of the *lsa*BGC GCF classifications, we discovered seven distinct GCFs corresponding to novel variants and genomic contexts for *pznAB* (Fig. S6a), including within genomes of species that are non-human skin-associated. Orthologous copies of *pznA* and *pznB* between GCFs were largely divergent in sequence and found in vastly different genomic contexts (Fig. S6ab), which could be the result of either ancestral HGT events where *pznAB* integrated into different genomic loci in different species or ancestral intra-genomic rearrangements.

## *lsa*BGC enables easy identification of incidents of HGT for individual GCFs

To evaluate more evident incidents of HGT occurring for individual GCFs, we systematically applied *lsa*BGC-Divergence, paired with Bayesian shrinkage analysis, for calculating Beta-RD distributions for each GCF from *Corynebacterium* and *Staphylococcus *. Beta-RD is a statistic that measures the sequence similarity between pairs of BGCs belonging to the same GCF normalized by the expected similarity of their genomic contexts. Thus, if two BGCs are highly similar in sequence but are found in vastly divergent genomes, such as those classified as different species, the Beta-RD statistic will be greater than one and indicate either high conservation or, potentially, the occurrence of recent HGT (Fig. S5cd; Table S6; Text S1).

**Fig. 3.**
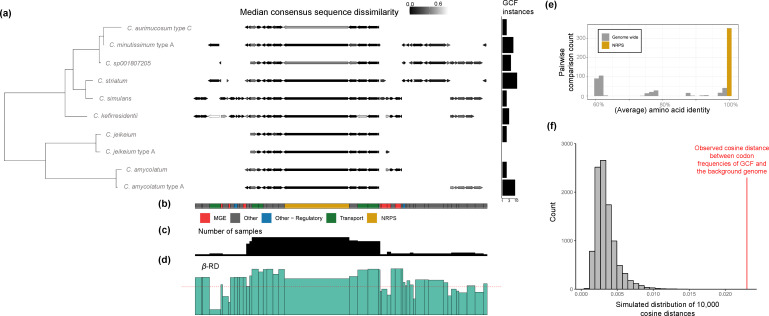
Evidence for HGT of an NRPS between divergent *Corynebacterium* species. (**a**) Phylogeny constructed from ribosomal proteins showing the conservation of *Corynebacterium* GCF-50 across the diverse species encoding the NRPS using results from *lsa*BGC-PopGene analysis. The gene schematics show the consensus gene order for the GCF. Gene lengths correspond to the median length across all BGC instances and only genes found in two or more genomes are shown. For each species, the gene is coloured based on the median similarity of gene sequences from the species to the consensus sequence across all species. The barplot displays the total number of GCF instances identified per species across the full set of *Corynebacterium* genomes. (**b**) Broad functional annotations for homologue groups are shown as a colour strip. (**c**) Below it are bar plots corresponding to the conservation of homologue groups across samples and (**d**) their median Beta-RD. (**e**) A histogram of the amino acid identities between GCF-50 NRP synthase instances that are complete (excluding partial instances due to assembly fragmentation included in panels (**a–d**) is compared to the average amino acid identities (AAI) between genomes they were gathered from. (**f**) compareBGCtoGenomeCodonUsage.py was used to infer the cosine distance between codon frequencies within GCF-50 and the background genome in one *Corynebacterium* isolate, and to compare this value to a simulated distribution of cosine distances for codon sets of equivalent size to the BGC.

High Beta-RD posterior ranges were observed for two NRPS containing GCFs from *Corynebacterium *. One of these GCFs, predicted to encode an NRPS-dependent siderophore [39], was found in several species commonly isolated from skin [37]. Using results from *lsa*BGC-PopGene for this GCF, which includes determination of a consensus gene order across BGC instances and a gene-specific calculation of Beta-RD, we find that the NRPS is highly conserved in sequence across species and found flanked by MGEs, as previously reported in *Corynebacterium jeikeium* [39] (Fig. 3a–e). The taxonomic breadth and high sequence conservation of this GCF suggests that it has undergone recent HGT between distantly related species.

We further assessed the likelihood of HGT for this GCF through the measurement of codon usage concordance between BGCs and their background genomes, using a standalone program provided in *lsa*BGC. Application of this program to the genome of *Corynebacterium simulans* strain PES1 showed that the observed cosine distance for the BGC was high relative to a simulated distribution of cosine distances based on coding genes across the full genome, further suggesting that the BGC was gained in the genome relatively recently and supporting horizontal acquisition (Fig. 3f).

## *lsa*BGC provides comprehensive and detailed views into evolutionary trends within GCFs

**Fig. 4.**
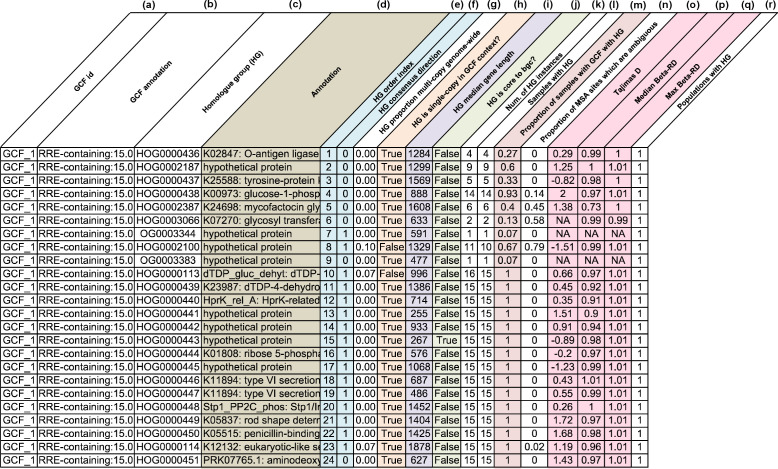
An example report produced by *lsa*BGC-PopGene. A report generated by *lsa*BGC-PopGene (using *lsa*BGC v1.31) was slightly modified (select columns presented, headers renamed, and colouring was manually added to highlight certain columns). The columns shown correspond to: (**a**) the GCF identifier, (**b**) the annotation of BGCs belonging to the GCF, (**c**) the homologue group identifier, (**d**) the annotation of the homologue group based on PGAP and KOfam profile HMMs (brown), (**e**) the consensus order and (**f**) the direction of the homologue group across BGC instances belonging to the GCF (light blue), (**g**) proportion of samples with GCF with paralogous instances of the homologue group across their genome, (**h**) whether the homologue group is found in single-copy per sample within the context of the GCF (orange), (**i**) the median length in bp of the homologue group (purple), (**j**) whether the homologue group contains a key domain used for BGC detection (green), (**k**) the number of instances of the homologue group found in the GCF, (**l**) the number of samples with the GCF with the homologue group, (**m**) the proportion of samples with the GCF with the homologue group – the conservation of the homologue group in the GCF (red), (**n**) the proportion of sites deemed as ambiguous in the codon alignment for the homologue group, (**o**) Tajima’s D, (**p**) median Beta-RD and max Beta-RD statistics computed for instances of the homologue group in the GCF (pink), and (**r**) the number of populations/sub-clades with the homologue group. Reports are automatically sorted by the consensus order inferred for homologue groups within the GCF.

*Lsa*BGC-PopGene is a core program in the suite that generates convenient reports on the annotation [40, 41], conservation and evolutionary trends of homologue groups found within a GCF (Fig. 4). We used *lsa*BGC-PopGene to infer selective pressures acting on homologue groups within GCFs through calculating inter- and intra-species Tajima’s D [42] using select representative genomes, obtained through genomic dereplication, for each genus, including *Cutibacterium* and *Micrococcus* (Tables S1, S7; Text S1). While *lsa*BGC-PopGene is highly scalable, it is important to perform uniform dereplication of genomes to accurately infer species-wide signatures of selection without being biased by highly represented strains in genomic datasets. Further, only homologue groups that were found as a single copy within the GCF context and observed in four of more genomes were considered. We found that the median intra-species Tajima’s D was centred at a value of roughly 0, in accordance with expectations of the null model (Fig. S7a). Also in alignment with expectations, we observed high values of genus-wide Tajima’s D for homologue groups in GCFs found in multiple species (Fig. S7b).

A focused intra-species analysis within *S. aureus* revealed that a multi-type terpene/typeIII PKS GCF (GCF-3) had the lowest Tajima’s D values and seven homologue groups with values below −2.0, suggestive that they are either highly conserved or, potentially, under sweeping selection (Fig. S7c). The terpene component of this hybrid GCF encodes for staphyloxanthin carotenoid production [43] and manual inspection determined that the type III PKS prediction actually corresponds to hydroxymethylglutaryl-CoA (HMG-CoA) synthase, which is involved in the mevalonate pathway for isoprenoid synthesis [44] and is homologous to KS domains of polyketide synthases [45]. Further, the regions in-between and flanking the staphyloxanthin encoding operon, *crt*, and HMG-CoA synthase include several genes important to staphylococcal virulence, some of which were found in multiple copies in certain species (Fig. S8ab). Because this GCF was predicted to be one of the four GCFs ancestrally acquired by the *S. epidermidis/aureus* clade, the five-gene *crt* operon encoded by it was found in most species belonging to the clade. Surprisingly however, *crt* genes were missing in more than 95 % of the *S. epidermidis* genomes, the most prevalent staphylococcal species on skin [29, 46], likely due to gene loss (Figs S5a, S8b).

### The virulence-associated carotenoid staphyloxanthin is ubiquitous across the genus *Staphylococcus*

**Fig. 5.**
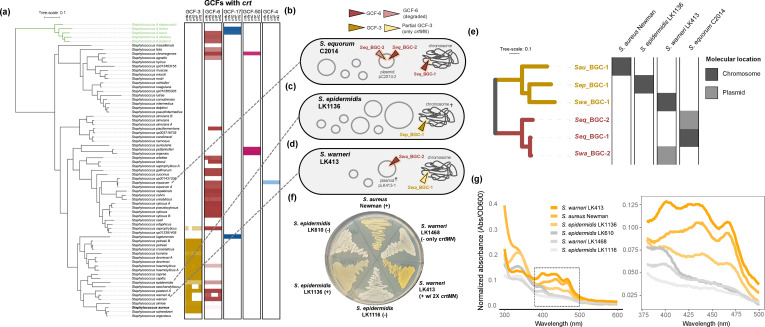
Staphyloxanthin is ubiquitous across *Staphylococcus *. (**a**) A maximum-likelihood phylogeny of the full genus *Staphylococcus* was constructed from ribosomal protein encoding genes. The clade in green represents the newly recognized genus *Mammaliicoccus *. Heatmaps showcase the presence of the five *crt* genes for each species for five separate GCFs containing the *crt* operon. The shading indicates the proportion of a species found to carry homologue groups within a particular GCF context in log_10_ scale. Schematics for the genomes of (**b**) *S. equorum* C2014, (**c**) *S. epidermidis* LK1136 and (**d**) *S. warneri* LK413 are shown with the location of GCFs with the *crt* operon marked. The symbol Ϯ signifies non-circular/incomplete scaffolds. (**e**) A maximum-likelihood phylogeny of CrtMN from GCF instances shown in panels (**b–d)** as well as *S. aureus* st. Newman is depicted with branch colouring based on GCF identity. The heatmap indicates which isolates the sequences correspond to as well as whether they were located on the chromosome or a plasmid. The degraded instance of the GCF-6 BGC in *S. equorum* is not included. (**f**) Representative isolates with and without staphyloxanthin encoding GCFs were grown on TSA to visualize pigmentation production. (**g**) Methanol extractions and wavelength absorption analysis were performed to identify signature peaks in the 400 to 500 nm range associated with the presence of staphyloxanthin in *S. aureus *. Spectra are normalized to cell density.

Although staphyloxanthin is well known as an *S. aureus* virulence factor [47, 48] and other *Staphylococcus* species have been reported as being pigmented [49, 50], we were unable to identify a report of the distribution of the *crt* operon across the genus *Staphylococcus *. To our knowledge, there are only two prior studies formally suggesting its production in one other non-*aureus* species, *Staphylococcus xylosus* [51, 52]. *lsa*BGC analysis revealed a total of five staphylococcal GCFs encoding for staphyloxanthin (Figs 5a, S9a). Following GCF-3, the second most prevalent GCF encoding staphyloxanthin, GCF-6, had a high Beta-RD distribution (Fig. S5c) and was widely distributed across divergent species in the genus (Fig. 5a), as well as *Mammaliicoccus,* the closest phylogenetic neighbours of staphylococci [53]. Constructing a phylogeny based on a concatenated alignment of CrtM and CrtN revealed the appropriate delineation of GCF-3 and GCF-6 despite their co-occurrence in the genomes of particular species, such as *Staphylococcus warneri* (Figs 5a, d, e, S9b). Additional analyses showed that GCF-6 can be found on plasmids and depicts signatures of inter-species HGT (Figs 5b,d,e, S10).

To assess whether carriage of the *crt* operon translates to staphyloxanthin production in non-*aureus* species, we identified staphylococcal isolates cultivated from human skin with whole-genome sequences generated by our laboratory that encode a staphyloxanthin-related GCF. We identified a rare instance of an *S. epidermidis* isolate with GCF-3 (only 2–3 % of *S. epidermidis* genomes encode GCF-3), strain LK1136, and an *S. warneri* isolate with both GCF-3 and GCF-6, strain LK413 (Fig. 5c,d). While we observe GCF-3 in all *S. warneri* genomes, the GCF is degraded and only encodes the dehydrosqualene synthase gene, *crtM*, and the dehydrosqualene desaturase gene, *crtN* (Fig. 5a,d; Table S8). Roughly half of the available *S. warneri* genomes (47.5%) encode GCF-6 in full and thus have two copies of *crtM* and *crtN*. We used long-read sequencing to generate near-complete genomes for *S. epidermidis* LK1136 and *S. warneri* LK413 and found that both strains encode GCF-3 within their chromosomes, while GCF-6 is encoded on a plasmid within *S. warneri *. (Fig. S9b; Text S1).

To test for phenotypic effects associated with strains carrying different staphyloxanthin GCFs, we assessed pigmentation levels in strains of *S. aureus *, *S. epidermidis* and *S. warneri* with and without full *crt* operons (Table S9). Cells of *S. epidermidis* encoding GCF-3 are pigmented compared to strains without the cluster (Fig. 5f). Cells of *S. warneri* LK413 containing the complete *crt* operon (GCF-6) and additional copies of *crtMN* displayed the strongest golden pigmentation, confirmed as staphyloxanthin by spectrophotometry based on absorbance profiles between 400 and 500 nm [43, 54] (Fig. 5g). The genomes of the six isolates were also confirmed to possess *aldH*, which encodes a sixth enzyme, located in trans from the *crt* operon, that is required for staphyloxanthin biosynthesis [55].

### *lsa*BGC provides a framework for mining for base resolution novelty in BGCs

We have demonstrated how the *lsa*BGC suite can be used to group homologous BGC instances into GCFs and subsequently uncover their evolutionary trends and phylogenetic distribution. With increasing metagenomic datasets becoming available, often from complex microbiomes, we developed *lsa*BGC-DiscoVary to directly profile BGCs and rapidly identify novel variation within GCF-related genes from metagenomes (Fig. S2; Text S1). This is particularly useful to understand the sequence conservation of genes from GCFs which are poorly represented in available genomic assembly databases. The *lsa*BGC framework allows users to first identify all GCF instances in genomic assemblies to build a comprehensive database of known alleles for GCF genes and subsequently apply *lsa*BGC-DiscoVary to find novel intragenic SNVs that are absent in available assemblies. Further, *lsa*BGC-DiscoVary extracts the subset of reads supporting the presence of novel SNVs, enabling use of exhaustive methods for validation of novel SNVs. Such validation would otherwise be computationally intensive and impractical to perform with the full set of reads from a metagenome.

First, we assessed the ability of *lsa*BGC-DiscoVary to properly determine the location of SNVs within genic codon alignments. We found a high concordance (>96 % overlap) between reported novel SNVs when compared to an assembly-based approach using whole-genome sequencing readsets for 132 *M *. *luteus* isolates (Fig. S11; Tables S10, S11; Text S1). Next, to demonstrate the precision of *lsa*BGC-DiscoVary when working with metagenomic datasets where multiple strains from a single species could be present, we applied it to profile novel SNVs across skin metagenomes in the recently characterized cutimycin synthesis encoding BGC from *C. acnes* (Text S1). Cutimycin, an anti-staphylococcal thiopeptide [22], is largely restricted to isolates belonging to either clades IB or III *C. acnes* (Fig. S12a), which is the most common species at sebaceous body sites [29]. In metagenomes where an independent software for strain presence assessment, StrainGST [36], detected both clade I and III *C. acnes* strains as present, *lsa*BGC-DiscoVary found novel SNVs specific to reference gene alleles gathered from both clade I and III genomes. However, as expected, for metagenomes where only clade I strains were detected by StrainGST, novel SNVs identified by *lsa*BGC-DiscoVary were never specific to reference gene alleles from clade III genomes (Fig. S12b).

### *lsa*BGC identifies novel SNVs within polyketide synthase catalytic domains

After validating the ability of *lsa*BGC-DiscoVary to reliably identify novel SNVs using single-isolate-read sets with paired assemblies and within the characterized GCF encoding for cutimycin biosynthesis, we applied it to less investigated GCFs across the *C. tuberculosteraricum* species complex. Phylogenomic [56] and ANI [57] investigations revealed that *C. tuberculostearicum* exhibits high genomic similarity (ANI >88 %) to four other species in GTDB (Figs S5b, S13a), which we refer to in aggregate as the *C. tuberculostearicum* species complex. We have since further investigated this species complex in a follow-up study [58]. While metagenomic profiling found that this complex features the most prevalent *Corynebacterium* species within healthy skin microbiomes (Fig. S5b, Table S4), only 22 genomic assemblies were available for the complex at the time of this analysis. This clade thus represents an ideal taxonomic group to examine novel BGC microdiversity in metagenomes.

**Fig. 6.**
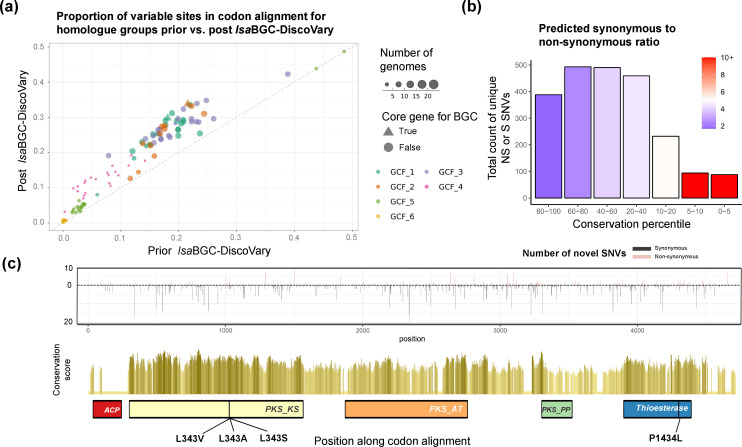
Thousands of novel SNVs identified within BGCs from the *C. tuberculostearicum* species complex. (**a**) A scatterplot showing the proportion of sites along BGC homologue group codon alignments with variability (at least two different alleles) before and after consideration of novel SNVs identified by *lsa*BGC-DiscoVary. (**b**) The number of novel SNVs is shown for ranges of conservation percentile across all homologue groups and metagenomes. The colouring corresponds to the ratio of synonymous to non-synonymous novel SNVs within each conservation range. (**c**) Novel SNVs are shown for the polyketide synthase involved in mycolic acid biosynthesis. The top panel showcases the non-synonymous (up-facing) and synonymous (down-facing) novel SNVs found along the T1PKS gene, with heights corresponding to the number of metagenomes with the novel SNV. The middle panel shows the conservation scores coloured by percentile ranges (lightest, least conserved sites; darkest, most conserved sites). Key domains common to T1PKS are shown underneath, along with the location of predicted non-synonymous mutations within the top 5 % of conserved sites for the gene. Two adjacent SNVs affecting the coding of the residue at position 343 were identified and these could result in a combined effect of encoding for alanine.

We ran *lsa*BGC-DiscoVary systematically on 6 GCFs identified in the species complex, largely encoding for unknown metabolites, and found 40 019 putatively novel SNV instances across 105 homologue groups and 109 metagenomic samples. Of these, 5474 instances were filtered because reads supporting their presence aligned with a higher score to other regions in a concatenated database of all *Corynebacterium* genomes from GTDB as compared to the GCF-specific reference gene databases used for *lsa*BGC-DiscoVary (Text S1). While ubiquitous, *C. tuberculostearicum* species complex members are present at very low abundances in some body sites where metagenomic assembly would struggle to construct their genomes (Fig. S13b; Text S1). We performed sample-specific metagenomic assemblies [59] and found that at least 11 659 novel SNV instances detected by *lsa*BGC-DiscoVary would be missed by assembly-based investigation (Fig. S13c; Text S1). In total, among the 103 homologue groups that had novel SNVs identified, *lsa*BGC-DiscoVary was able to increase the proportion of variable sites along their codon alignments on average by 5.7 % (Fig. 6a).

To further investigate the novel SNVs detected, we focused on 68 context-distinct homologue groups overlapping or near protocore regions of BGCs and further filtered based on standardized coverage metrics, retaining 5802 instances of 2343 unique novel SNVs. Novel SNVs were more likely to correspond to synonymous changes if they were found in multiple metagenomic samples (Fig. S13d; Text S1). Further, metagenomic samples from the same subject at different body sites and from the same body sites across different subjects were more likely to share novel SNVs, compared to metagenomic samples from different body sites and different subjects (*P*<1E-8; two-sided Wilcoxon rank sum test) (Fig. S13e; Text S1).

Finally, we expected and observed that the ratio of predicted synonymous to non-synonymous novel SNVs increases at deeply conserved sites along homologue groups (Fig. 6b; Table S12; Text S1). The homologue group with the greatest number of novel SNVs, and novel, non-synonymous SNVs within highly conserved regions, was the PKS gene responsible for synthesis of mycolic acid, a major component of the cell wall in most *Corynebacterium* species recently shown to have immunomodulatory effects on the skin [25, 60, 61]. We identified and manually validated three non-synonymous SNVs along conserved sites of the PKS gene, including two adjacent SNVs predicted to affect one codon within the ketoacyl synthase domain, which catalyzes condensation [62], and could alter reaction kinetics [63] or scaffold structure [64] (Figs 6c, S14; Table S13).

## Discussion

The *lsa*BGC suite packages several fundamental, as well as novel, functionalities for high-throughput comparative and evolutionary analysis of BGCs from any taxa in a structured framework, such as the ability to detect GCFs within draft-quality genomic assemblies. Of practical relevance, it produces a comprehensive spreadsheet that includes annotation, conservation and evolutionary statistics for homologue groups found in GCFs to enable efficient and high-throughput assessment by users. By incorporating GToTree [56], *lsa*BGC will also automatically generate visualizations showing the distribution of GCFs across a species phylogeny. However, for more constrained taxonomic investigations, which might focus on a single lineage, more resolute whole-genome phylogenies might be appropriate to use.

Paralogy of homologue groups or full BGCs is an important consideration when interpreting evolutionary statistics [64, 65]. As such, we now clearly mark which homologue groups do not correspond to single-copy orthologues within GCF contexts in resulting reports produced by *lsa*BGC and have made it the default setting to use more resolute hierarchical orthogroups determined by OrthoFinder, which are better able to partition paralogous proteins.

The metagenomic mining functionalities of *lsa*BGC further provide insight into the presence and sequence diversity of a specific taxa’s BGCs across diverse microbiomes. This is a particularly important feature for rare BGCs and taxa with a limited number of genomic assemblies available, such as taxa that are difficult to cultivate from complex metagenomes. *lsa*BGC-DiscoVary prioritizes the reduction in false positives at the expense of incurring false negatives using a multitude of conditional assessments. While these settings should be generally applicable to most short-read sequencing metagenomic datasets, users should assess the conditions to ensure appropriate application for their research aims. A current limitation of *lsa*BGC-DisoVary is the inability to discover novel SNVs in intergenic regions within BGCs, which might be important for transcriptional regulation, and should be considered for future versions of the suite. The use of pan-genome alignment approaches [66] to potentially increase the efficiency of *lsa*BGC for metagenomic identification of novel variants within BGC genes may also be explored.

Here, we apply *lsa*BGC’s functionalities to major taxa commonly found in skin microbiomes as a proof of concept to demonstrate the potential to advance understanding of the diversity, evolutionary trends and prevalence of both well-studied and uncharacterized secondary metabolites. For example, we uncovered the ubiquity of the carotenoid staphyloxanthin across the genus *Staphylococcus,* a molecule conferring the signature golden pigmentation in *S. aureus* [49, 50]. These findings raise the hypothesis that staphyloxanthin, which has been identified primarily as a virulence factor conferring oxidative stress resistance in the pathogen *S. aureus* [47, 67], could similarly contribute to virulence in other staphylococcal species or serve additional unknown functions. Supporting this hypothesis, we observe that one staphyloxanthin encoding GCF appears to be ancestral to the skin-associated, multi-species clade that features both *S. aureus* and *S. epidermidis *. The observation that multiple copies of *crt* genes can be found within a single genome further suggests that staphyloxanthin is of functional importance, since evolutionary retention of paralogous genes has previously been shown to underlie ecological advantages [68, 69]. Additionally, while this GCF is present in most skin-associated species, it is absent in >95 % of *S. epidermidis* genomes, suggesting large-scale loss within the species. It will thus be interesting to further explore how the loss of staphyloxanthin, if it contributes to virulence in *S. epidermidis *, coincides with the species’ reputation as a beneficial symbiont of host skin [29, 70].

Using the novel metagenomic mining functionality of *lsa*BGC, we identified novel SNVs distinguishing uncultured *C. acnes* and *Corynebacterium* strains from isolates with genomes available in public databases. We further determined that some of the most common skin-residing species [28, 37] belonging to the *C. tuberculostearicum* species complex, vastly underrepresented in public databases, contain thousands of novel SNVs within the BGCs of the clade. Critically, some of these variants could have functional implications, as they lie within highly conserved regions of important enzymatic domains for metabolite biosynthesis.

The comprehensive and open-source packaging of *lsa*BGC allows analyses to be applied to both public and proprietary microbial genomic databases. Besides accelerating fundamental evolutionary research of BGCs from diverse taxa, we envision that *lsa*BGC will be particularly useful for natural product discovery and guiding mechanism-of-action studies. Evolutionary insight gathered from *lsa*BGC can be used as prior information to train algorithms in an emerging field using artificial intelligence to identify new antibiotics [71, 72]. Further, an improved understanding of the relationship between intra-taxa genomic diversity and BGC content novelty at base resolution can also highlight which sub-lineages or sub-clades bear the largest reservoir of untapped secondary metabolic potential. Finally, we posit that identifying evolutionary trends of BGCs detected by highly reliable rule-based approaches, such as antiSMASH [9], can be used to assess the validity of BGC predictions from newly developed machine learning approaches, such as DeepBGC [12] or GECCO [73], which we now support use of in *lsa*BGC.

## Methods

### Overview of *lsa*BGC programs and workflows

*lsa*BGC v1.0 was used for the analyses in this study unless otherwise noted. Since the initial release of the suite*,* changes to core programs have largely been minimal and updates have largely focused on improving the interface through newly introduced programs to simplify usage of the suite. One of the new programs is the *lsa*BGC-Easy, which is our current recommended workflow for running *lsa*BGC, and can be run using a simple one-line command on any focal bacterial taxa of interest. Programs and workflows provided in *lsa*BGC are described in depth in the Text S1 subsection ‘Descriptions of *lsa*BGC programs and algorithms’ and on its Github wiki. Benchmarking analyses described in the Results are explained in greater detail in the Text S1 section ‘Benchmarking Analyses for *lsa*BGC-AutoExpansion and *lsa*BGC-DiscoVary’. Brief descriptions of the individual programs and workflows within *lsa*BGC are provided here with greater details on algorithms, considerations and parameters described in Text S1.

***lsa*** **BGC-Cluster** (**core program**) clusters homologous and non-fragmented instances of BGCs, identified by antiSMASH, similar to BiG-SCAPE [10]. Users can then generate a report to select for the most appropriate parameters for downstream analyses of interest.

***lsa*BGC-Expansion** (**core program**) can directly identify GCF instances within genomes, following gene calling, using an HMM-based approach that is similar to ClusterFinder [11]. It uses profile hidden Markov models (HMMs) to identify homologues of GCF-associated genes in assemblies followed by a classical HMM approach to define BGC regions and their boundaries. It is specifically designed to account for BGC fragmentation due to incomplete genomic assemblies. The ***lsa*BGC-AutoExpansion** (**workflow**) provides a systematic workflow for identification of GCFs determined from BGCs in a primary or core set of genomes in a separate, additional set of genomes, which might be of draft quality. Use of *lsa*BGC-AutoExpansion is recommended instead of *lsa*BGC-Expansion individually because the workflow additionally features a consolidation step in which BGC instances identified as potentially belonging to multiple GCFs are reassessed and assigned to only the single best fitting GCF (Fig. S1c).

***lsa*BGC-Refiner** (**core program**) provides the option to manually separate hybrid or multi-class GCFs based on user-defined boundary homologue groups.

***lsa*** **BGC-See** (**core program**) permits visualization of BGCs across a user-provided species tree or a BGC-based phylogeny and can handle multiple fragmented BGC instances from the same genome, depicting them as neighbouring leaves on phylogenies. **GSeeF.py** (**auxiliary program**) is a standalone program that can visualize GCFs as a heatmap across a species phylogeny using either *lsa*BGC or BiG-SCAPE results.

***lsa*BGC-PopGene** (**core program**) can then infer evolutionary and population genetics statistics for each homologue group associated with a GCF. It produces a convenient table report that makes assessment of homologue group syntenic context, annotation and evolutionary metrics easy for users (Fig. 3).

***lsa*BGC-Divergence** (**core program**) calculates a comprehensive statistic between pairs of homologous BGCs from the same GCF but different genomes, which we refer to as Beta-RD, that captures how similar the BGC sequences are relative to what would be expected based on genome-wide similarity. **compareBGCtoGenomeCodoneUsage.py** (**auxiliary program**) is a standalone program that can compare the codon frequencies observed in a BGC to the background genomic context using standard output from antiSMASH [9].

***lsa*BGC-DiscoVary** (**core program)** is a multi-functional program that can mine raw sequencing readsets from metagenomes or single genomes for GCF instances and then reliably identify whether they possess novel single-nucleotide variants (SNVs) yet to be observed in available genomic assemblies for a taxon (Fig. S2).

***lsa*** **BGC-AutoProcess** (**workflow**) was the original workflow for processing user-provided genomes into the input files required for downstream *lsa*BGC analyses (Fig. S1a). The workflow, used for the analyses described in this study, performs gene calling and standard annotation using Prokka [32], identifies BGCs using antiSMASH [9] and determines homologue groups using OrthoFinder [14]. In newer versions of *lsa*BGC, the worklow is no longer supported and has been replaced with ***lsa*BGC-Ready** (**core program**), which provides similar functionalities and can reformat existing BGC prediction results, as well as clustering into GCFs performed using BiG-SCAPE [10], to generate the input files needed for *lsa*BGC.

***lsa*BGC-AutoAnalyze** (**workflow**) runs the core analytical programs *lsa*BGC-PopGene, *lsa*BGC-See, *lsa*BGC-PopGene and, optionally, *lsa*BGC-DiscoVary for each GCF and creates a few consolidated reports and visualizations after completion. Earlier versions of the workflow, which were used in this study, ran CompareM [74] or FastANI [57] to estimate genome-wide expected differences, whereas in the latest *lsa*BGC releases, this information is instead provided as an argument and calculated upstream in *lsa*BGC-Ready using core-gene alignments generated by GToTree [56].

***lsa*BGC-Easy** (**workflow**) simplifies the application of *lsa*BGC by allowing users to perform the majority of analyses by simply issuing a single command. It can automatically perform BGC prediction, using GECCO [73] by default, dereplicate genomes, download genomes for a specific taxon and run *lsa*BGC-Ready and *lsa*BGC-AutoAnalyze.

### Use of genomic and metagenomic datasets

To overcome issues with misclassification and contamination, we used GTDB (release 202) [75] to query for reliable genomic assemblies and respective species designations for the genera *Corynebacterium *, *Cutibacterium *, *Micrococcus* and *Staphylococcus* (Table S1). Two staphylococcal genomes (GCF_900098335.1 and GCF_000276505.1) were dropped from analysis due to failure to process in *lsa*BGC-AutoProcess. For *Micrococcus *, we further included 132 additional genomes of the type species *M. luteus* that our laboratory had sequenced (BioProject ID PRJNA803478) but had not yet been uploaded to NCBI to be included in the GTDB release used. These isolates were classified as *M. luteus* by GTDB-tk [76] and exhibited >95 % ANI to the NCBI representative genome for the species *M. luteus* NCTC 2665. A similar approach was used for gathering genome sets for species-level analyses performed (Table S1). For the *C. tuberculostearicum* species complex, an additional five genomes from NCBI were gathered, classified using GTDB-tk [76] and incorporated into analysis if they belonged to one of the five species designated as belonging to the complex.

Because a single representative per species as provided by GTDB might not adequately capture the diversity of BGCs found across a genus, we instead employed a more granular approach to select for representative genomes for a genus. We performed dereplication using MASH [77] with an estimated ANI cutoff of 0.99 to pair similar genomes and dynamically select representative genomes from each cluster based on assembly N50 (Table S1). This dereplication was performed using the script popSizeAndSampleSelector.py provided in the *lsa*BGC suite. Complete or chromosome-level assemblies among the representative genomes were run through lsaBGC-AutoProcess.py and lsaBGC-Cluster.py to identify the core set of GCFs per genus. GCFs determined from the core set of genomes were comprehensively identified in the remaining genomes using lsaBGC-AutoExpansion.py, including those that were not deemed as representatives from the MASH based analysis to showcase scalability (e.g. all 15 055 *Staphylococcus* and not just the 229 representative genomes, of which 77 corresponded to complete/chromosome-level assemblies). To expedite population genetics analysis, however, we only ran the downstream functional analytics of *lsa*BGC using the representative genomes for each genus. For species-level analyses, all genomes available were used for such downstream analyses and to comprehensively profile known alleles for genes in BGCs, which was critical to enable metagenomic mining for base resolution novelty, previously unseen in assemblies available for a species. For population-level analyses within species, strain delineations were performed using the popSizeAndSampleSelector.py script in *lsa*BGC with strain groups determined from single-linkage clustering of pairs of genomes found to exhibit >98 % ANI and >80 % genomic content similarity by FastANI [57].

For determining skin-associated species amongst the genera investigated in our study and to demonstrate mining functionalities with *lsa*BGC-DiscoVary, we used a paired-end metagenomics dataset sequenced on the Illumina NovaSeq, where individual samples corresponded to microbiome surveys of 1 of 8 body sites for 1 of 34 participants, collected in the US state of Wisconsin (BioProject ID PRJNA763232) [37]. As previously described [37], adapter removal, quality filtering, human sequence decontamination and tandem repeat removal were performed using fastp (v0.21.0) [78] and KneadData (v0.8.0) (https://huttenhower.sph.harvard.edu/kneaddata/).

### Genomic assessment for staphyloxanthin carriage in the laboratory isolate collection

We had previously constructed draft genomic assemblies for 44 *Staphylococcus* isolates from skin (BioProject ID PRJNA803478; BioProject ID PRJNA830888). Illumina sequencing was performed at the Microbial Genome Sequencing Center (MiGS). Briefly, assemblies were generated using Unicycler [79] with default settings after quality and adapter trimming using fastp [78]. The set of 44 *Staphylococcus* genomes were searched for carriage of staphyloxanthin encoding GCFs identified in our study using *lsa*BGC-AutoExpansion (Table S9).

### Library preparation, sequencing and genomic assembly

#### *Reconstruction of Illumina draft assemblies for 132 *M. luteus

We assessed the performance of multiple programs in the *lsa*BGC suite using a set of genomic assemblies for 132 *M *. *luteus* isolates that were cultured from skin. For these isolates, assemblies were reconstructed from raw reads (BioProject ID PRJNA803478) and were uploaded to the NCBI’s GenBank database (Table S1). Briefly, Illumina sequencing reads were processed using fastp (v 0.20.1) [78] to trim for Poly-G tail artifacts and TrimGalore (v 0.6.5) [80] was additionally used to detect and remove adapters. Afterwards, assembly was performed using Unicycler (v 0.4.6) [79] with default settings.

#### *Construction of Illumina+Nanopore hybrid assemblies for staphyloxanthin-producing* S. warneri *LK413 and* S. epidermidis *LK1136*

To learn the genomic location of staphyloxanthin-encoding GCFs in *S. epidermidis* LK1136 and *S. warneri* LK413, we performed additional long-read sequencing using Oxford Nanopore Technologies (ONT). Library construction, using a PCR-free ligation method, and sequencing were performed at the Microbial Genome Sequencing Center (MiGS). Hybrid assemblies were constructed using a modified version of the Hybrid Assembly workflow in seQuoia (https://github.com/broadinstitute/seQuoia). Illumina reads, used for construction of the initial draft genomic assemblies for the isolates, were first reprocessed using fastp (v 0.20.1) [78] to trim for Poly-G tail artifacts and TrimGalore (v 0.6.5) [80] was subsequently used to detect and remove adapters. Similarly, ONT FASTQ reads, provided by MIGS after basecalling, were filtered for potential adapters using PoreChop (v 0.2.3) (https://github.com/rrwick/Porechop) and filtered to retain only reads >3 kb in length as well as subsampled to 300 Mb (while being inclusive of all reads >20 kb) using the script fastqfilter.py included in seQuoia. Afterwards, hybrid assemblies were constructed using Unicycler (v 0.4.6) [79] with default settings and additional Pilon (v 1.23) [81] polishing was performed iteratively by aligning ONT reads [82], as well as the Illumina reads [83], to assemblies and refining them, allowing for a maximum of 10 iterations. Quality assessment of the two near-complete assemblies was performed using GAEMR (https://github.com/broadinstitute/GAEMR) (Text S1).

### Experimental validation of staphyloxanthin production in non-*aureus Staphylococcus*

Isolates were grown overnight at 37 °C on tryptone soy agar plates and inoculated into tryptone soy broth at a concentration of 1×10^6^ c.f.u. ml^−1^. Liquid cultures were grown in shaking conditions at 37 °C for 24 h; 10 ml of the liquid culture was then pelleted and washed twice with phosphate-buffered saline (PBS) before pigment extraction in 1 ml of methanol at 55 °C for 15 min. Spectral scans from 300 to 600 nm were taken using a plate reader (EPOCH2, Biotek, Winooski, VT, USA). Absorbance readings were blanked with methanol and normalized to liquid culture cell density based on OD_600_. The spectra shown are representative of at least two biological replicates.

### MASH distance neighbour-joining tree construction

Average nucleotide identity estimates between pairs of genomes were computed with MASH [77] and used to create a distance matrix. The distance matrix was then used to construct a neighbour-joining tree with the ape library in R [84].

### Phylogenetic constructions

#### Ribosomal phylogeny construction

Ribosomal phylogenies for each species were constructed based on a concatenated alignment of 16 ribosomal proteins [85]. HMMER (v3) was used to search for profile HMMs of each ribosomal protein in the predicted proteomes of samples of interest belonging to a particular taxonomic group. Afterwards, the best matching hits for each sample based on alignment E-value were identified and used to construct individual alignments for each ribosomal protein with the l-INS-I method in MAFFT [86]. Protein alignments were then converted to codon alignments using PAL2NAL [87] and subsequently concatenated together. Samples that featured >25 % gaps in the concatenated alignment were filtered, after which core SNV sites were extracted and used to construct a maximum-likelihood phylogeny with RAxML [88], specifying a GTRCAT model and performing 100 bootstraps. Where needed, ribosomal phylogenies were subset to showcase relationships between select samples using PareTree (http://emmahodcroft.com/PareTree.html; v1.0.2).

#### Staphyloxanthin phylogeny construction

A maximum-likelihood phylogeny was constructed from a concatenated protein alignment of CrtM and CrtN amongst representative *Staphylococcus* genomes to understand the relationship between different GCFs encoding for staphyloxanthin. Proteins designated as OG0001876 (CrtM) and OG0001904 (CrtN) were gathered for each staphyloxanthin-encoding GCF found in each sample and aligned using the l-INS-I method in MAFFT [86]. To account for assembly fragmentation resulting in multiple proteins from a sample being assigned to the same homologue group for a specific GCF, a consensus sequence was determined and multi-allele sites were replaced as gaps. A concatenated alignment of such consensus sequences was subsequently constructed and used for phylogeny construction with RAxML using a PROTCAT model with JM selected as the best amino acid replacement matrix through the automatic maximum-likelihood model selector [88]. The phylogeny of CrtMN sequences exhibited high correspondence to GCF delineations, with the exception of a single *S. lugdunensis* BGC, which is likely misclassified as GCF-17 and should be GCF-3. Upon further examination, it was identified that the reason for the misclassification was likely due to the absence of mevalonate pathway genes, classified as a type III polyketide synthase (T3PKS), within *S. lugdunensis *, which are found downstream of the *crt* operon in all other species with GCF-3 (Figs S9a, S10a).

#### Pyrazinone phylogeny construction

A maximum-likelihood phylogeny was constructed from a concatenated protein alignment of PznA and PznB amongst representative *Staphylococcus* genomes using the same methods as described for phylogeny construction from CrtMN proteins.

#### *Validation of the* C. tuberculostearicum *species complex as a monophyletic clade*

To validate that the *C. tuberculostearicum* species complex is a monophyletic clade, GToTree was used to construct an approximate maximum-likelihood phylogeny of all 1118 *Corynebacterium* genomes considered in this study, including all 22 *C *. *tuberculostearicum* species complex members, using FastTree [89] built off a concatenated alignment of 138 genes regarded as single-copy core for Actinomycetota.

### Strain detection and ubiquity assessment of skin-residing species of *Staphylococcus *, *Corynebacterium* and *Cutibacterium*

We searched for the presence of representative *Staphylococcus *, *Corynebacterium* and *Cutibacterium* strains in 270 skin metagenomes from 34 participants across 8 body sites using StrainGST [36]. To construct the database of distinct representative genomes for each genus, the protocol described in the StrainGE documentation was followed. *S. aureus* genomes 16 405 and 278 were dropped from the analysis because at the time of downloading the plasmid and chromosome were switched. Briefly, genomes were downloaded from NCBI using ncbi-genome-download (https://github.com/kblin/ncbi-genome-download) and dereplication was performed using built-in StrainGE functionalities (v 0.1+191.g7fcfbcd.dirty). For the *Cutibacterium* database, the incomplete *C. acnes* Asn12 genome was added manually to include a representative from clade III. As some representative genomes were not part of GTDB release R202, GTDB-tk [76] was used to annotate such genomes and perform species classification.

For *Corynebacterium* and *Staphylococcus *, we considered a species as skin-associated if it was found in at least 10 of the 270 skin metagenomes, regardless of the abundance (Table S4). Representative genomes that GTDB-tk analysis classified as non-* Corynebacterium* (*n*=1; not found in metagenomes) or *Corynebacterium* species not yet catalogued in database release R202 (*n*=2; each detected in only one metagenome) are not shown in Table S4. Certain skin-associated species from each genus were also identified in the 19 negative control metagenomic samples, which included air-swab, bench, desk and door-handle samplings (Table S4). Because we expect heavy overlap in species content between these environmental samples and skin microbiome samples, we still considered such species as skin-associated if they were found in 10 or more skin metagenomes.

We found that the majority of skin-associated *Staphylococcus* belonged to the *S. epidermidis/aureus* clade, as has previously been noted [45]. Similarly, most of the skin-associated *Corynebacterium* belonged to *C. tuberculostearicum* and its close neighbouring species (including *C. kefirresidentii*, *C. aurimucosum* type E, *C*. sp. 900539985, *C. tuberculostearicum *, *C. tuberculostearicum* type C), which we denote in this study as the *C. tuberculostearicum* species complex. The minimal ANI separating genomes belonging to this species complex was determined to be 88.59 % using FastANI [57]. To assess whether any of the 24 new *Corynebacterium* species reported by Saheb Kashaf *et al*. [31] belonged to this species complex, FastANI was used to look at their ANI similarity to genomes from the clade and regard them as additional members if they exhibited 88.59 % ANI or greater. Of note, many additional *Corynebacterium* species have been routinely isolated from the skin [37], which we did not regard as skin-associated based on our criteria in this study. In particular, the species of *C. amycolatum* (detected in four metagenomes), *C. kroppenstedtii* (detected in four metagenomes) and *C. simulans* (detected in five metagenomes) are commonly found on skin.

The relative abundance prediction for *Corynebacterium* strains in metagenomic samples and species was taken as the *rapct* metric reported by StrainGST (v 1.3.3; Figure S13b).

### Calculation of Tajima’s D for orthologues in BGCs from *Staphylococcus*

For the comprehensive analysis of Tajima’s D reported in this study, we only considered homologue groups that were found in four or more genomes and were strictly single-copy within GCF contexts (Table S7). We investigated Tajima’s D values for common homologue groups across all four genera in our study and found that they reflected expectations of a null model, with the median intra-species Tajima’s D being centred at a value of roughly 0 (Fig. S7a). When calculating Tajima’s D in aggregate across species, a large fraction of homologue groups (17.3%) were found to exhibit high values (>2), suggesting balancing selection; however, most of these homologue groups appear to be ancestrally acquired and likely diverged in sequence simply due to speciation rather than targeted selective pressures (Fig. S7b). To better discern true instances of balancing selection (Tajima’s D >2) and conservation or sweeping selection (Tajima’s D <−2), we compared the aggregate genus-wide Tajima’s D with the maximum and minimum value of Tajima’s D observed for a single species (Fig. S7b). Species-specific Tajima’s D values for *S. aureus* strict single-copy orthologues (in the GCF and species context) highlighted the staphyloxanthin-encoding GCF-3 as featuring the greatest number of highly conserved homologue groups (Tajima’s D <−2) (Fig. S7c).

### Bayesian shrinking analysis to assess Beta-RD distributions for GCFs in *Staphylococcus* and *Corynebacterium*

To better understand how distributions of Beta-RD differ across GCFs, in this study, we applied a Bayesian hierarchical model to perform shrinking analysis and appropriately account for differences in the number of genomes GCFs that are found (Fig. S5cd; Table S6). For computational efficiency, a maximum of 500 Beta-RD observations were used for each GCF. Posterior predictions for Beta-RD were made using rstan (Stan Development Team, 2021) with the following hierarchical model:

$$\bar{\mu }∼norm\left(\mu_{0},\sigma_{0}^{2}\right)$$

$$\tau ∼half-t_{7}\left(1\right)$$

$$\mu_{1}\ldots \mu_{J}∼norm\left(\bar{\mu },\tau \right)$$

$$\sigma ∼half-t_{7}$$

$$y_{j}∼norm\left(\mu_{j},\sigma^{2}\right)$$

where for each GCF *j*, the posterior Beta-RD distribution is sampled as *y*_*j*_ using Markov chain Monte Carlo with four chains, each featuring 2000 iterations, including a burn-in of 1000 iterations. A hierarchical prior was specified for *μ*_*j*_, which represents a GCF-specific baseline. The values of *μ*_0_ and σ_0_ were set to the respective mean and standard deviation of raw Beta-RD values across all GCFs. R and STAN code used for this analysis are available within the scripts subdirectory of the *lsa*BGC software package.

### Phylogenetic and gene cluster visualizations

We used the iTol webserver [90] and packages in R, including ggtree [91] and gggenes, to construct custom phylogenetic and gene cluster visualizations for our study. Example visualizations produced by *lsa*BGC-See and GSeeF.py are provided on the *lsa*BGC GitHub wiki and are similarly based on these plotting frameworks.

For visualization of staphyloxanthin encoding GCFs across the *Staphylococcus* phylogeny (Figs 4a, S8b), only a single representative genome from each species, as classified by GTDB [75], was selected and used to prune the original ribosomal phylogeny created from the diversity representation genome set. The percentage of species members with GCFs for these figures was determined using *lsa*BGC-AutoExpansion results for all ~15K *Staphylococcus* genomes, and was shown in log_10_ scale. For generating comparative views between different *crt* encoding GCF representatives (Fig. S9a) we used clinker [92].

## Supplementary Data

Supplementary material 1

Supplementary material 2

## References

[1] Salamzade R, Alex Cheong JZ, Sandstrom S, Swaney MH, Stubbendieck RM (2023). Figshare.

[2] Katz L, Baltz RH (2016). Natural product discovery: past, present, and future. J Ind Microbiol Biotechnol.

[3] Watve MG, Tickoo R, Jog MM, Bhole BD (2001). How many antibiotics are produced by the genus *Streptomyces*?. Arch Microbiol.

[4] Hopwood DA (2007). Streptomyces in nature and medicine: The antibiotic makers.

[5] Chevrette MG, Carlson CM, Ortega HE, Thomas C, Ananiev GE (2019). The antimicrobial potential of *Streptomyces* from insect microbiomes. Nat Commun.

[6] Waterworth SC, Flórez LV, Rees ER, Hertweck C, Kaltenpoth M (2020). Horizontal gene transfer to a defensive symbiont with a reduced genome in a multipartite beetle microbiome. mBio.

[7] Drott MT, Rush TA, Satterlee TR, Giannone RJ, Abraham PE (2021). Microevolution in the pansecondary metabolome of *Aspergillus flavus* and its potential macroevolutionary implications for filamentous fungi. Proc Natl Acad Sci.

[8] Steinke K, Mohite OS, Weber T, Kovács ÁT (2021). Phylogenetic distribution of secondary metabolites in the *Bacillus subtilis* species complex. mSystems.

[9] Blin K, Shaw S, Kloosterman AM, Charlop-Powers Z, van Wezel GP (2021). antiSMASH 6.0: improving cluster detection and comparison capabilities. Nucleic Acids Res.

[10] Navarro-Muñoz JC, Selem-Mojica N, Mullowney MW, Kautsar SA, Tryon JH (2020). A computational framework to explore large-scale biosynthetic diversity. Nat Chem Biol.

[11] Cimermancic P, Medema MH, Claesen J, Kurita K, Wieland Brown LC (2014). Insights into secondary metabolism from a global analysis of prokaryotic biosynthetic gene clusters. Cell.

[12] Hannigan GD, Prihoda D, Palicka A, Soukup J, Klempir O (2019). A deep learning genome-mining strategy for biosynthetic gene cluster prediction. Nucleic Acids Res.

[13] Kautsar SA, van der Hooft JJJ, de Ridder D, Medema MH (2021). BiG-SLiCE: a highly scalable tool maps the diversity of 1.2 million biosynthetic gene clusters. Gigascience.

[14] Emms DM, Kelly S (2019). OrthoFinder: phylogenetic orthology inference for comparative genomics. Genome Biol.

[15] Crits-Christoph A, Diamond S, Butterfield CN, Thomas BC, Banfield JF (2018). Novel soil bacteria possess diverse genes for secondary metabolite biosynthesis. Nature.

[16] Sugimoto Y, Camacho FR, Wang S, Chankhamjon P, Odabas A (2019). A metagenomic strategy for harnessing the chemical repertoire of the human microbiome. Science.

[17] Andreu VP, Augustijn HE, Chen L, Zhernakova A, Fu J (2021). A systematic analysis of metabolic pathways in the human gut microbiota. Bioinformatics.

[18] Pereira-Flores E, Medema M, Buttigieg PL, Meinicke P, Glöckner FO (2021). Mining metagenomes for natural product biosynthetic gene clusters: unlocking new potential with ultrafast techniques. Bioinformatics.

[19] Chevrette MG, Handelsman J (2021). Needles in haystacks: reevaluating old paradigms for the discovery of bacterial secondary metabolites. Nat Prod Rep.

[20] Chase AB, Sweeney D, Muskat MN, Guillén-Matus DG, Jensen PR (2021). Vertical inheritance facilitates interspecies diversification in biosynthetic gene clusters and specialized metabolites. mBio.

[21] Zipperer A, Konnerth MC, Laux C, Berscheid A, Janek D (2016). Human commensals producing a novel antibiotic impair pathogen colonization. Nature.

[22] Claesen J, Spagnolo JB, Ramos SF, Kurita KL, Byrd AL (2020). A *Cutibacterium acnes* antibiotic modulates human skin microbiota composition in hair follicles. Sci Transl Med.

[23] Nakatsuji T, Chen TH, Narala S, Chun KA, Two AM (2017). Antimicrobials from human skin commensal bacteria protect against *Staphylococcus aureus* and are deficient in atopic dermatitis. Sci Transl Med.

[24] Wyatt MA, Wang W, Roux CM, Beasley FC, Heinrichs DE (2010). *Staphylococcus aureus* nonribosomal peptide secondary metabolites regulate virulence. Science.

[25] Ridaura VK, Bouladoux N, Claesen J, Chen YE, Byrd AL (2018). Contextual control of skin immunity and inflammation by *Corynebacterium*. J Exp Med.

[26] Swaney MH, Kalan LR (2021). Living in your skin: microbes, molecules, and mechanisms. Infect Immun.

[27] Grice EA, Kong HH, Conlan S, Deming CB, Davis J (2009). Topographical and temporal diversity of the human skin microbiome. Science.

[28] Oh J, Byrd AL, Park M, Kong HH, NISC Comparative Sequencing Program (2016). Temporal stability of the human skin microbiome. Cell.

[29] Byrd AL, Belkaid Y, Segre JA (2018). The human skin microbiome. Nat Rev Microbiol.

[30] Acosta EM, Little KA, Bratton BP, Mao X, Payne A Bacterial DNA on the skin surface overrepresents the viable skin microbiome. Microbiology.

[31] Saheb Kashaf S, Proctor DM, Deming C, Saary P, Hölzer M (2022). Integrating cultivation and metagenomics for a multi-kingdom view of skin microbiome diversity and functions. Nat Microbiol.

[32] Seemann T (2014). Prokka: rapid prokaryotic genome annotation. Bioinformatics.

[33] Hyatt D, Chen G-L, Locascio PF, Land ML, Larimer FW (2010). Prodigal: prokaryotic gene recognition and translation initiation site identification. BMC Bioinformatics.

[34] Cacho RA, Tang Y, Chooi Y-H (2014). Next-generation sequencing approach for connecting secondary metabolites to biosynthetic gene clusters in fungi. Front Microbiol.

[35] Gilchrist CLM, Booth TJ, van Wersch B, van Grieken L, Medema MH (2021). cblaster: a remote search tool for rapid identification and visualization of homologous gene clusters. Bioinform Adv.

[36] van Dijk LR, Walker BJ, Straub TJ, Worby CJ, Grote A (2022). StrainGE: a toolkit to track and characterize low-abundance strains in complex microbial communities. Genome Biol.

[37] Swaney MH, Sandstrom S, Kalan LR (2022). Cobamide sharing is predicted in the human skin microbiome. mSystems.

[38] Zimmermann M, Fischbach MA (2010). A family of pyrazinone natural products from a conserved nonribosomal peptide synthetase in *Staphylococcus aureus*. Chem Biol.

[39] Tauch A, Kaiser O, Hain T, Goesmann A, Weisshaar B (2005). Complete genome sequence and analysis of the multiresistant nosocomial pathogen *Corynebacterium jeikeium* K411, a lipid-requiring bacterium of the human skin flora. J Bacteriol.

[40] Aramaki T, Blanc-Mathieu R, Endo H, Ohkubo K, Kanehisa M (2019). KofamKOALA: KEGG ortholog assignment based on profile HMM and adaptive score threshold. Bioinformatics.

[41] Li W, O’Neill KR, Haft DH, DiCuccio M, Chetvernin V (2021). RefSeq: expanding the prokaryotic genome annotation pipeline reach with protein family model curation. Nucleic Acids Res.

[42] Tajima F (1989). Statistical method for testing the neutral mutation hypothesis by DNA polymorphism. Genetics.

[43] Pelz A, Wieland K-P, Putzbach K, Hentschel P, Albert K (2005). Structure and biosynthesis of staphyloxanthin from *Staphylococcus aureus*. J Biol Chem.

[44] Misic AM, Cain CL, Morris DO, Rankin SC, Beiting DP (2016). Divergent isoprenoid biosynthesis pathways in *Staphylococcus* species constitute a drug target for treating infections in companion animals. mSphere.

[45] Balibar CJ, Shen X, Tao J (2009). The mevalonate pathway of *Staphylococcus aureus*. J Bacteriol.

[46] Grice EA, Kong HH, Renaud G, Young AC, Bouffard GG (2008). A diversity profile of the human skin microbiota. Genome Res.

[47] Clauditz A, Resch A, Wieland K-P, Peschel A, Götz F (2006). Staphyloxanthin plays a role in the fitness of *Staphylococcus aureus* and its ability to cope with oxidative stress. Infect Immun.

[48] Holt DC, Holden MTG, Tong SYC, Castillo-Ramirez S, Clarke L (2011). A very early-branching *Staphylococcus aureus* lineage lacking the carotenoid pigment staphyloxanthin. Genome Biol Evol.

[49] Kloos WE, Schleifer KH (1975). Isolation and characterization of *Staphylococci* from human skin II. descriptions of four new species: *Staphylococcus warneri*, *Staphylococcus capitis*, *Staphylococcus hominis*, and *Staphylococcus* simulans. Int J Syst Bacteriol.

[50] Becker K, Heilmann C, Peters G (2014). Coagulase-negative *Staphylococci*. Clin Microbiol Rev.

[51] Vermassen A, Dordet-Frisoni E, de La Foye A, Micheau P, Laroute V (2016). Adaptation of *Staphylococcus xylosus* to nutrients and osmotic stress in a salted meat model. Front Microbiol.

[52] Seel W, Baust D, Sons D, Albers M, Etzbach L (2020). Carotenoids are used as regulators for membrane fluidity by *Staphylococcus xylosus*. Sci Rep.

[53] Madhaiyan M, Wirth JS, Saravanan VS (2020). Phylogenomic analyses of the *Staphylococcaceae* family suggest the reclassification of five species within the genus *Staphylococcus* as heterotypic synonyms, the promotion of five subspecies to novel species, the taxonomic reassignment of five *Staphylococcus* species to *Mammaliicoccus* gen. nov., and the formal assignment of *Nosocomiicoccus* to the family *Staphylococcaceae*. Int J Syst Evol Microbiol.

[54] Dong P-T, Mohammad H, Hui J, Leanse LG, Li J (2019). Photolysis of Staphyloxanthin in methicillin-resistant *Staphylococcus aureus* potentiates killing by reactive oxygen species. Adv Sci.

[55] Kim SH, Lee PC (2012). Functional expression and extension of staphylococcal staphyloxanthin biosynthetic pathway in *Escherichia coli*. J Biol Chem.

[56] Lee MD (2019). GToTree: a user-friendly workflow for phylogenomics. Bioinformatics.

[57] Jain C, Rodriguez-R LM, Phillippy AM, Konstantinidis KT, Aluru S (2018). High throughput ANI analysis of 90K prokaryotic genomes reveals clear species boundaries. Nat Commun.

[58] Salamzade R, Swaney MH, Kalan LR (2023). Comparative genomic and metagenomic investigations of the *Corynebacterium tuberculostearicum* species complex reveals potential mechanisms underlying associations to skin health and disease. Microbiol Spectr.

[59] Li D, Liu C-M, Luo R, Sadakane K, Lam T-W (2015). MEGAHIT: an ultra-fast single-node solution for large and complex metagenomics assembly via succinct de Bruijn graph. Bioinformatics.

[60] Gande R, Gibson KJC, Brown AK, Krumbach K, Dover LG (2004). Acyl-CoA carboxylases (accD2 and accD3), together with a unique polyketide synthase (Cg-pks), are key to mycolic acid biosynthesis in Corynebacterianeae such as *Corynebacterium glutamicum* and *Mycobacterium tuberculosis*. J Biol Chem.

[61] Portevin D, De Sousa-D’Auria C, Houssin C, Grimaldi C, Chami M (2004). A polyketide synthase catalyzes the last condensation step of mycolic acid biosynthesis in mycobacteria and related organisms. Proc Natl Acad Sci.

[62] Chen Y, Kelly EE, Masluk RP, Nelson CL, Cantu DC (2011). Structural classification and properties of ketoacyl synthases. Protein Sci.

[63] Klaus M, Buyachuihan L, Grininger M (2020). Ketosynthase domain constrains the design of polyketide synthases. ACS Chem Biol.

[64] Medema MH, Cimermancic P, Sali A, Takano E, Fischbach MA (2014). A systematic computational analysis of biosynthetic gene cluster evolution: lessons for engineering biosynthesis. PLoS Comput Biol.

[65] Rokas A, Mead ME, Steenwyk JL, Raja HA, Oberlies NH (2020). Biosynthetic gene clusters and the evolution of fungal chemodiversity. Nat Prod Rep.

[66] Garrison E, Sirén J, Novak AM, Hickey G, Eizenga JM (2018). Variation graph toolkit improves read mapping by representing genetic variation in the reference. Nat Biotechnol.

[67] Liu C-I, Liu GY, Song Y, Yin F, Hensler ME (2008). A cholesterol biosynthesis inhibitor blocks *Staphylococcus aureus* virulence. Science.

[68] Blount ZD, Barrick JE, Davidson CJ, Lenski RE (2012). Genomic analysis of a key innovation in an experimental *Escherichia coli* population. Nature.

[69] Ghosh S, O’Connor TJ (2017). Beyond paralogs: the multiple layers of redundancy in bacterial pathogenesis. Front Cell Infect Microbiol.

[70] Otto M (2009). *Staphylococcus* epidermidis--the “accidental” pathogen. Nat Rev Microbiol.

[71] Stokes JM, Yang K, Swanson K, Jin W, Cubillos-Ruiz A (2020). A deep learning approach to antibiotic discovery. Cell.

[72] Melo MCR, Maasch J, de la Fuente-Nunez C (2021). Accelerating antibiotic discovery through artificial intelligence. Commun Biol.

[73] Carroll LM, Larralde M, Fleck JS, Ponnudurai R, Milanese A (2021). Accurate de novo identification of biosynthetic gene clusters with GECCO. Bioinformatics.

[74] Parks D CompareM: A toolbox for comparative genomics. https://github.com/dparks1134/CompareM.

[75] Parks DH, Chuvochina M, Rinke C, Mussig AJ, Chaumeil P-A (2022). GTDB: an ongoing census of bacterial and archaeal diversity through a phylogenetically consistent, rank normalized and complete genome-based taxonomy. Nucleic Acids Res.

[76] Chaumeil P-A, Mussig AJ, Hugenholtz P, Parks DH (2019). GTDB-Tk: a toolkit to classify genomes with the Genome Taxonomy Database. Bioinformatics.

[77] Ondov BD, Treangen TJ, Melsted P, Mallonee AB, Bergman NH (2016). Mash: fast genome and metagenome distance estimation using MinHash. Genome Biol.

[78] Chen S, Zhou Y, Chen Y, Gu J (2018). fastp: an ultra-fast all-in-one FASTQ preprocessor. Bioinformatics.

[79] Wick RR, Judd LM, Gorrie CL, Holt KE (2017). Unicycler: resolving bacterial genome assemblies from short and long sequencing reads. PLoS Comput Biol.

[80] Krueger F, James F, Ewels P, Afyounian E, Schuster-Boeckler B FelixKrueger/TrimGalore. https://zenodo.org/record/5127899.

[81] Walker BJ, Abeel T, Shea T, Priest M, Abouelliel A (2014). Pilon: an integrated tool for comprehensive microbial variant detection and genome assembly improvement. PLoS One.

[82] Li H, Birol I (2018). Minimap2: pairwise alignment for nucleotide sequences. Bioinformatics.

[83] Li H, Durbin R (2009). Fast and accurate short read alignment with Burrows–Wheeler transform. Bioinformatics.

[84] Paradis E, Claude J, Strimmer K (2004). APE: Analyses of Phylogenetics and Evolution in R language. Bioinformatics.

[85] Hug LA, Baker BJ, Anantharaman K, Brown CT, Probst AJ (2016). A new view of the tree of life. Nat Microbiol.

[86] Katoh K, Standley DM (2013). MAFFT multiple sequence alignment software version 7: improvements in performance and usability. Mol Biol Evol.

[87] Suyama M, Torrents D, Bork P (2006). PAL2NAL: robust conversion of protein sequence alignments into the corresponding codon alignments. Nucleic Acids Res.

[88] Stamatakis A (2014). RAxML version 8: a tool for phylogenetic analysis and post-analysis of large phylogenies. Bioinformatics.

[89] Price MN, Dehal PS, Arkin AP (2010). FastTree 2--approximately maximum-likelihood trees for large alignments. PLoS One.

[90] Letunic I, Bork P (2019). Interactive Tree Of Life (iTOL) v4: recent updates and new developments. Nucleic Acids Res.

[91] Yu G, Smith DK, Zhu H, Guan Y, Lam T-Y (2017). Ggtree: an r package for visualization and annotation of phylogenetic trees with their covariates and other associated data. Methods Ecol Evol.

[92] Gilchrist CLM, Chooi Y-H (2021). Clinker & clustermap.js: automatic generation of gene cluster comparison figures. Bioinformatics.

