# Supplementary material for: Evolutionary investigations of the biosynthetic diversity in the skin microbiome using lsaBGC
Source: Microb Genom. 2023 Apr 28;9(4):mgen000988. doi: 10.1099/mgen.0.000988 (PMC10210951; doi:10.1099/mgen.0.000988)
Supplement: Supplementary material 2 [file mgen-9-988-s002.pdf]

Supplementary Text and Figures

Evolutionary investigations of the biosynthetic diversity in the skin microbiome using *lsa*BGC

Salamzade R, Cheong JZA, Sandstrom S, Swaney MH, Stubbendieck RM,
Lane Starr N, Currie CR, Singh AM, and Kalan LR

Contents

Descriptions of *lsa*BGC programs and algorithms 2

_lsa_BGC-AutoProcess and _lsa_BGC-Ready: Gene calling, standard and BGC annotation, and homology inference 2

_lsa_BGC-Cluster: Clustering of BGCs into GCFs 2

_lsa_BGC-Expansion: High-throughput identification of homologous instances of GCFs in assemblies 3

_lsa_BGC-See and GSeeF.py: Visualization of BGCs across phylogenies 5

_lsa_BGC-PopGene: Assessing the conservation, annotation, and evolution of genes within GCFs 5

_lsa_BGC-Divergence and compareBGCtoGenomeCodonUsage.py: Assessing the divergence of BGCs relative to their background genomic contexts 6

_lsa_BGC-DiscoVary: Metagenomic mining for BGCs, homolog groups, and novel SNVs 7

Overview of the _lsa_BGC-Easy workflow 11

Updates to _lsa_BGC since release 1.0 11

Benchmarking Analyses for _lsa_BGC-AutoExpansion and _lsa_BGC-DiscoVary 12

Additional Details on Analyses in Study 15

Parameters and thresholds used for clustering GCFs and subsequent annotation 15

Ancestral inference of gene cluster family carriage 15

Identification of homolog groups associated with skin-residing species of _Staphylococcus_ and _Corynebacterium_ 15

Inspection of additional GCFs from _Staphylococcus_ and _Corynebacterium_ with high Beta-RD values 16

Assessment of hybrid genomic assemblies constructed for _S. epidermidis_ LK1136 and _S. warneri_ LK413 ... 16

Designation of species belonging to _Mammaliicoccus_ 16

_lsa_BGC-DiscoVary based assessment of cutimycin presence in skin metagenomes 17

_lsa_BGC-DiscoVary based analyses of BGCs from the _C. tuberculostearicum_ species complex 17

Supplementary Text References 18

Supplementary Figures 24

Descriptions of *lsa*BGC programs and algorithms

*lsa*BGC-AutoProcess and *lsa*BGC-Ready: Gene calling, standard and BGC annotation, and homology inference

For this study, complete or chromosome level assemblies were run through lsaBGC-AutoProcess.py, which invokes Prokka[1] for gene calling and annotation, antiSMASH for BGC detection (v6)[2], and OrthoFinder2[3] for delineating homologous clusters of proteins (Fig. S1a). Since version 1.1, we no longer recommend usage of lsaBGC-AutoProcess.py and instead suggest using the lsaBGC-Easy.py workflow or lsaBGC-Ready.py. lsaBGC-Ready.py can perform many of the same functions as lsaBGC-AutoProcess.py with expanded options to: (i) automatically perform clustering of BGCs into GCFs using lsaBGC-Cluster.py or to incorporate user-provided BiG-SCAPE[4] clustering results, (ii) identify additional instances of GCFs directly in draft quality assemblies using lsaBGC-AutoExpansion.py, and (iii) construct a species phylogeny using GToTree[5].

Briefly, lsaBGC-Ready.py performs gene-calling on genomes using prodigal[6], consolidates gene calling with those present in BGC GenBanks, performs annotation using KOfam and PGAP profile HMMs[7,8], and can automatically run lsaBGC-Cluster.py and lsaBGC-AutoExpansion.py. lsaBGC-Ready.py takes as input genomes and BGC predictions (from either antiSMASH, GECCO, or DeepBGC) and does not perform BGC prediction in contrast to lsaBGC-AutoAnalyze.py. The program can optionally generate a species phylogeny using GToTree and use the core-genome alignment constructed in the process to estimate genome to genome expected similarities, removing the need for CompareM[9] and FastANI[10] as dependencies of *lsa*BGC to simplify the installation process.

*lsa*BGC-Cluster: Clustering of BGCs into GCFs

lsaBGC-Cluster.py leverages homology information from OrthoFinder2[3] together with syntenic similarity akin to the approach taken in BiG-SCAPE[4]. However, unlike BiG-SCAPE, which is reliant on domains, lsaBGC-Cluster.py uses entire protein sequences designated to discrete homolog groups, offers a tailored report for users on appropriate parameter selection, and employs Markov chain clustering (MCL)[11] instead of affinity propagation for granular GCF delineation. Similar to BiG-SCAPE, lsaBGC-Cluster.py also uses both the presence of shared coding units (domains for BiG-SCAPE; homolog groups for lsaBGC-Cluster.py) as well as sequence similarity between such units to appropriately cluster BGCs. In lsaBGC-Cluster.py, the presence of homolog groups and their sequence similarity is implicit to the OrthoFinder2 algorithm which determines appropriate thresholds for designating homologous, ideally orthologous, protein instances by accounting for genome-wide similarity[3]. Also, similar to BiG-SCAPE in concept[4], lsaBGC-Cluster.py can be set to require syntenic similarity between BGC instances for clustering, but the methodology in lsaBGC-Cluster.py is based on global syntenic similarity measured using the absolute value of the Pearson's correlation for sets of $\geq$3 homolog groups shared between BGC instances. BiG-SCAPE on the contrary uses more localized information on adjacent-pairs of domains to infer overall syntenic similarity. Finally, both lsaBGC-Cluster.py and BiG-SCAPE offer flexibility for whether to perform preliminary partitioning of BGCs based on the predicted class by antiSMASH. By default, lsaBGC-Cluster.py does not perform such partitioning.

Unique to lsaBGC-Cluster.py is an option to produce a user-friendly PDF report guiding users for optimal parameters for clustering. This report showcases how different values of the MCL inflation parameter and the Jaccard similarity threshold for shared homologs between pairs of BGCs affects the final clustering. In this report, users can see how different parameter combinations can influence the number of singleton GCFs, GCFs with a single BGC member, the number of the core homolog groups observed in all BGCs belonging to a particular GCF (Table S2, S3). Such a report is critical for users to be able to move forward with an appropriate delineation of GCFs and not to re-track and manually experiment with different clustering configurations. An example report, pertaining to clustering BGCs from 77 *Staphylococcus* genomes with completed or chromosome-level assemblies, as well as descriptions for

each of the figures featured in its 52 pages can be found on the Github wiki page for the lsaBGC-Cluster.py program.

To additionally provide users with versatility to better define the boundaries of a GCF, which could be particularly useful when dealing with hybrid BGCs or multiple BGCs co-located nearby each other, we further provide lsaBGC-Refiner.py. lsaBGC-Refiner.py takes in as input a GCF listing file of BGC instances belonging to it and a pair of user-defined boundary homolog groups. It then filters BGC Genbanks to retain only genes found in between the two boundary homolog groups. This functionality is particularly useful for instances where antiSMASH is inconsistent in defining hybrid BGCs due to variable inter-protocore content which has recently been highlighted[12].

*lsa*BGC-Expansion: High-throughput identification of homologous instances of GCFs in assemblies

To mine metagenomic datasets for base-resolution novelty within BGCs previously unobserved from available assemblies for a given taxa, it is first necessary to comprehensively profile all allelic variants of a homolog group from such a set of assemblies. We thus developed lsaBGC-Expansion.py to efficiently and systematically identify orthologous instances of GCFs across the comprehensive set of assemblies available for a taxa, which for certain *Staphylococcus* species could range in the thousands on NCBI's GenBank database (Fig. 2, S1b). In concept, lsaBGC-Expansion.py is similar to the now common bioinformatics practice of defining homolog groups for proteins upfront and then searching for their presence in new genomes[7,13,14]. In fact, re-identifying instances of homolog groups found within GCFs is the first component of lsaBGC-Expansion.py. This is performed by first constructing profile HMMs for such homolog groups using MAFFT local alignment[15] with standard settings and HMMER3[16], followed by searching genomic assemblies directly using HMMER3 or emitting the consensus sequence and searching via DIAMOND[17], as inspired by the approach developed by Melnyk et al. 2019[13]. For the analyses presented in this paper, we used the DIAMOND-based approach (specified by --quick_mode argument).

In a preliminary step, profile HMMs are similarly aligned to the predicted proteome of genomes from the initial set of genomes which were run through antiSMASH and used to establish GCFs. This reflexive alignment enables the determination of appropriate E-value thresholds to gauge the presence or absence of homolog groups in new genomes. Specifically, the E-value thresholds for each homolog group is determined as either: (i) the lowest E-value of false positive alignments multiplied by a factor of 1E-5 or (ii) a default threshold of 1E-10 if E-values for some true positive alignments are found to be higher than those for false positive alignments. Importantly, this methodology for selecting E-value thresholds assumes that new genomes being searched are phylogenetically interspersed with genomes used for initial analysis and construction of the profile HMMs. We additionally required proteins which aligned to homolog group profile HMMs to also be of similar length to the proteins used to construct the profile HMMs, requiring them to be at most 1.5X the max(25, median absolute divergence) base-pairs shorter or longer than the median length of the representative proteins of the homolog group.

A classical HMM framework, leveraging the pomegranate library[18], is used to scan predicted coding genes across assembly scaffolds, where coding genes are regarded to follow a binary state based on whether they exhibit homology to a GCF-associated homolog group. Our approach mimics the algorithm of ClusterFinder[19], but features several important differences to identify fragmented instances matching well-characterized BGCs rather than search for novel BGCs with remote homology to known instances. We estimate the emission probability of each homolog group based on whether it's profile HMM is able to distinguish true hits from false hits from reflexive alignments (see previous paragraph). If it is able to distinguish true-hits from false-hits, then detecting a homolog group at the E-value threshold described above is very likely to represent a predicted protein belongs to the GCF and the homolog group will have an emission probability of 0.99 for the "GCF State" and an emission probability of 0.01 for the "Background State". If the profile HMM is unable to distinguish true-hits from false-hits, then the emission probability for the "Background State" is set to a maximum of either: (i) 1.0 - (# of GCF

instances / # of total instances) or (ii) 0.2. The emission probability for the "GCF State" in this case will be set to the complement of the "Background State" probability. The transition probabilities between states are set to 0.1 by default and to 0.9 for transitioning to the same state. The start and end of a scaffold are equally likely to correspond to either the states of GCF or Background.

Genomic neighborhoods of predicted coding genes detected by the HMM as corresponding to the GCF (herein referred to as "potential GCF segments") are next conditionally assessed to avoid reporting false positive neighborhoods which are likely not related to the GCF in question. Each potential GCF segment must feature at least 3 homolog groups found in a known instance of the GCF. Additionally, all potential GCF segments must display reasonable syntenic similarity (gene positioning) to a known instance of the GCF found in the high-quality genomic assemblies. Correlations are only calculated if the genes in the segment under consideration and the comparing known BGC instance have genes in the same relative direction and are found in single copy in the context of the GCF. A default correlation of 0.8 to at least one known GCF instance is required by each segment (with p-value < 0.1).

Segments are reported as part of the GCF automatically if any of the following criteria are met: (i) The segment features >= 5 homolog groups and segment features >= 3 "core" homolog groups ("core" homolog groups are those which are observed in all known instances of a GCF from the high-quality genomic assemblies), (ii) the segment features a GCF "specific" homolog group (these are homolog groups which are only observed within the GCF in the high-quality genome assemblies, thus their presence is a good indication the segment belongs to the GCF and is not a false positive), or (iii) the segment features homolog groups which overlap with the core of protoclusters in GCFs as delineated by antiSMASH. If segments do not meet any of the three criteria above, they can still be reported if they are on the edge of scaffolds (within 2,000 bp from the end of a scaffold). These segments do not need to meet criteria (i) individually but do need to feature >=5 homolog groups and >=3 core homolog groups of the GCF in unison. Additionally, only one edge segment per scaffold is allowed. Finally, a single "protocore" homolog group (for which ≥50% belong to protocore regions in antiSMASH BGC predictions) is required to be present in at least one of segments meeting the criteria described for the GCF to be regarded as present in a genome and for segments to be reported.

To ensure we are not missing more evolutionarily diverged variants of homolog groups not represented in the initial lsaBGC analysis, we perform a final "polishing" step for segments which are considered to belong to a GCF. This involves reassessing genes on GCF segments which are unassigned to any homolog group and additionally searching for surrounding genes (up to 5 genes on each side) which display homology to GCF-associated homolog groups. A gene only needs to display homology at a level of less than 1E-10 E-value to be assigned to a homolog group. Critically, we only assign these genes to homolog groups after the segment is determined as belonging to the focal GCF to ensure our detection of segments is based on more concrete/significant homology-based evidence

Ultimately, we do not recommend running lsaBGC-Expansion.py individually on a GCF-by-GCF basis, but to instead use the wrapper workflow program lsaBGC-AutoExpansion.py, to automatically search for homologous instances across all GCFs. This is because lsaBGC-AutoExpansion.py is able to resolve potential conflicts where some part of a genomic assembly might independently be assigned to two separate GCFs (Fig. S1c). After running lsaBGC-Expansion.py individually per GCF, it consolidates results and resolves any conflict in genes from the same genomic assembly being assigned to multiple GCFs. It resolves such conflicts by performing pairwise comparisons of the gene sets across every BGC instance from every GCF. Overlap between two BGCs from distinct GCFs is considered a conflict if they overlap with more than 5% of the number genes of one of the BGCs. If this is the case, the sum of the exponents of E-values of each BGC's genes to their respective homolog group profile HMMs are compared and the BGC with the lower sum (indicating more genes in the BGC match a GCF profile or that genes match better to the GCF profile) is retained while the other is discarded. lsaBGC-AutoExpansion.py also creates consolidated result files, including: (i) an updated/expanded sample vs. homolog group gene matrix, (ii) an updated/expanded sample listings file (listing the path to each

sample's GenBank assembly and predicted proteome), and (iii) an updated listing file of BGCs for each GCF.

*lsa*BGC-See and GSeeF.py: Visualization of BGCs across phylogenies

We have provided the lsaBGC-See.py program to allow users to visualize GCF instances across either a user-provided species phylogeny or automatically generated GCF-specific phylogeny. While the latter functionality is present in CORASON[4], the former feature, which can provide a differing and important evolutionary perspective, is currently unique to *lsa*BGC. *lsa*BGC creates automated PDF reports using R visualization libraries ggplot2[20] and ggtree[21] as well as creates a track for visualization in iTol[22]. Additionally, the constructed GCF phylogeny and user-provided species phylogeny are reformatted to a Newick file in which genome identifiers are expanded if multiple instances of a GCF are found for it, which is critical for visualizing fragmented BGCs from draft genomes. The program generates a GCF specific phylogeny if requested by creating codon alignments, concatenating these, filtering for conserved SNVs and generating an approximate maximum-likelihood phylogeny using FastTree2[23].

Since version 1.3, we also provide the largely standalone program GSeeF.py within the *lsa*BGC suite, which allows for construction of species phylogenies using GToTree[5] and phylogenomic heatmaps showcasing the presence of GCFs, defined by lsaBGC-Cluster.py or BiG-SCAPE, with the coloring of the heatmap corresponding to annotation types of BGCs extracted from antiSMASH BGC GenBanks. GSeeF.py can be used to create similar views such as what we have depicted in Fig. 2C.

*lsa*BGC-PopGene: Assessing the conservation, annotation, and evolution of genes within GCFs

A core program of the *lsa*BGC suite is lsaBGC-PopGene.py which generates a table report of conservation and evolutionary statistics for each homolog group found within a specific GCF. To calculate certain homolog group statistics within this report, lsaBGC-PopGene.py begins by constructing protein alignments[15] for each homolog group and translating those to codon-based alignments using PAL2NAL[24]. Codon-based alignments are additionally used to visualize conservation and domain structure for homolog groups and downstream in lsaBGC-DiscoVary.py. Three of the evolutionary statistics which codon alignments are used for computing are: (i) homolog-group specific variants of the Beta-Relative Divergence statistic, (ii) the rate of non-synonymous mutations relative to the rate of synonymous mutations (dN/dS), and (iii) Tajima's D statistic, which can be used to detect signatures of sweeping vs. balancing selection. lsaBGC-PopGene.py also allows users to specify population designations for each isolate with the focal GCF and computes additional statistics per homolog group pertaining to this information.

Assuming users provide genome-wide similarity estimates, either ANI or AAI, to lsaBGC-PopGene.py, it will calculate how pairwise homolog group similarities in codon alignments or protein alignments compare to such genome-wide expectations. This is reported as the median Beta-RelativeDivergence (analogous to the BGC-wide metric described in the following subsection) across all pairs of genomes with the homolog group found in the focal GCF. Note, in more recent versions of *lsa*BGC, expected genome-wide similarities are estimated from a core protein alignment generated by GToTree.

Tajima's D is a simple statistic where values below -2 typically indicate conservation or sweeping selection and a low ratio of rare minor alleles to high-frequency minor alleles while values above 2 indicate balancing selection. To calculate Tajima's D we modified a previous implementation[25] into a function within *lsa*BGC. Namely, we adjusted the calculation of Tajima's D to better reflect the mathematical derivation described in Tajima 1989[26] and account for the presence of gaps in alignments. Gaps are first minimized in codon alignments by filtering sequences which exhibited ≥25% of their sites as gaps and subsequently filtering codon sites where ≥10% of the retained sequences featured gaps and no polymorphism was observed. Pairwise differences are only considered when bases mismatched and not when a gap was observed in one or both of the sequences being compared. Segregating sites were regarded as sites where valid pairwise differences were observed. A minimum of 4 sequences, 21 codon

sites, and 3 segregating sites in filtered codon alignments are required for calculation of the statistic. As an alternate assessment of sequence variation to Tajima's D, we also report the proportion of sites across homolog group multiple sequence alignments where multiple alleles exist (major allele < 98%) and the proportion of sites where the major allele is non-dominant (<75%).

The rate of non-synonymous mutations relative to the rate of synonymous mutations is a classical statistic used to infer the effect of positive versus negative selection. If the rate for non-synonymous mutations is higher than the rate for synonymous mutations across different instances of a gene (dN/dS > 1), it could suggest positive selection; whereas, if the reverse is true (dN/dS < 1), it could suggest negative or purifying selection. We used Biopython's codonalign.codonseq module[27] to calculate dN/dS using the method described by Nei and Gojobori 1986[28]. Codon alignments were similarly filtered to minimize the presence of gaps as described for the calculation of Tajima's D. Additionally, to ensure we avoid excessive computation when the number of sequences with a homolog group is large, we have implemented an empirical but non-exhaustive framework in which we calculate the median dN/dS between 1000 randomly selected pairs of samples. This random sampling and calculation of dN/dS is performed for 20 iterations and the median of dN/dS estimates across iterations, along with the absolute median deviation to assess robustness, is reported. Because we are actively seeking to improve our calculations of dN/dS in *lsa*BGC and Biopython's codonalign module is under development currently, we do not discuss results related to dN/dS in this study.

Inference of consensus order and directionality of homolog groups for a GCF: We developed an algorithm to infer the consensus order of homolog groups relative to each other in the GCF, as well as their relative consensus directionality (sense vs. antisense). This algorithm works by first computing how many times a homolog group proceeds another homolog group across BGC instances belonging to a GCF. To gather this information, a single BGC instance, with the most homolog groups, is selected as the reference and used for configuring the general direction of the remaining GCF instances, deciding whether to flip them to better align with the order of the reference. The gene order information for each BGC, encoded as a dictionary, is then used to construct a primary path, starting from the homolog group most often found at one edge of a GCF to the homolog group most often found on the opposite edge. Afterwards, homolog groups which were not featured in this consensus path, potentially because they are infrequently found and not core to the GCF, are attempted to be placed in their most appropriate locations. The core ordering path will be structured primarily by more prevalent homolog groups. When looking at the lsaBGC-PopGene.py report table sorted by the consensus ordering of the homolog groups, it is thus important to consider the proportion of samples with the GCF which have a particular homolog group. The consensus directionality of homolog groups is simply based on whether most instances are in the same or opposite direction relative to a reference gene from the chosen representative BGC.

*lsa*BGC-Divergence and compareBGCtoGenomeCodonUsage.py: Assessing the divergence of BGCs relative to their background genomic contexts

BGC divergence does not always track with background genomic divergence and speciation[29]. The program *lsa*BGC-Divergence allows for easy assessment of how BGC divergence compares to whole-genome divergence, primarily through computing a statistic we refer to as Beta-Relative-Divergence (Beta-RD) between pairs of genomes for a single GCF. The Beta-RD statistic is simply the average amino or nucleotide identity between homologous instances of proteins for a GCF observed in two genomes normalized by the average amino or nucleotide identity observed between the full genomes. *lsa*BGC allows users to specify whether amino or nucleotide divergence should be used and automated workflows can be used to compute pairwise AAI or ANI between genomes using CompareM (https://github.com/dparks1134/CompareM) or FastANI[10], respectively. In more recent versions of the suite, the core-genome alignment produced by GToTree to produce a species tree is instead used to estimate genome-to-genome expected similarities and make installation of the *lsa*BGC suite easier.

Codon usage of individual genes can be compared to the general distribution of all coding genes across a genome to infer potential genomic islands[30]. Depending on when the horizontal gene transfer event occurred, genes horizontally inherited could potentially assimilate for codon usage within the genome through vertical evolution. We developed a standalone program, compareBGCtoGenomeCodonUsage.py, provided in *lsa*BGC as a ready to use executable for calculating codon usage differences between coding genes within BGCs of a particular GCF and genes observed outside of the GCF context. Briefly, this program calculates the frequency of codons for coding genes within BGCs and then compares those to the frequency of codons observed in the complementary set of coding genes across the genome. For comparison of the two codon frequency distributions, the program computes both the cosine distance and Spearman's correlation rho. Genes of a length not divisible by three are ignored.

In *lsa*BGC v1.31, we introduced an option to further calculate an empirical P-value for inferring the significance of observing as high a cosine distance between BGC coding genes and genome-wide coding genes through simulating a distribution of expected cosine distances. In each of the 10,000 simulations performed to construct the distribution, genes across the genome are shuffled in order and codons are selected from the first G genes until C codons have been gathered, where C is the number of codons observed in the BGC. The cosine distance is then computed between the frequency of codons selected to the frequency of codons in the remainder of codons. The empirical P-value is simply the proportion of occurrences that a simulated cosine distance is larger than the observed cosine distance for the GCF with a pseudocount of one appended to the numerator and denominator.

*lsa*BGC-DiscoVary: Metagenomic mining for BGCs, homolog groups, and novel SNVs

Metagenomic methods to explore microdiversity within microbiomes are continually advancing[31,32]. We developed lsaBGC-DiscoVary.py to explore the micro-diversity of BGCs beyond the limited set of single-isolate genomes available for a lineage or taxa of interest and allow users to identify BGC genes and base-resolution novel SNVs within metagenomic datasets (Fig. S2). Briefly, lsaBGC-DiscoVary.py serves two roles:

1. Assessing the presence of GCFs, individual homolog groups and if requested, phasing them (uses a custom approach leveraging DESMAN[33]), and
2. If users construct a comprehensive database of homolog groups alleles observed across all available assemblies for a taxonomy, lsaBGC-DiscoVary.py can also be used to identify putatively novel SNVs never previously observed at a particular site in a homolog group.

<u>Selection of Representative Alleles for Reference Database:</u> lsaBGC-DiscoVary.py begins by identifying representative allelic sequences for each homolog group in the focal GCF from the codon alignments listing file provided. To select representative alleles, it parses each homolog group's MSA FASTA file, considers sequences within a single median absolute deviation from the median length, and determines the number of differences between pairs of sequences. Sequences are deemed to be members of the same allelic cluster if they exhibit ≥99% identity of the shorter sequence and they differ at less than 10 sites from each other. Afterwards, pairs of such similar sequences are joined into larger clusters through single-linkage clustering and a representative sequence is chosen based on the minimal summed differences to the other sequences in the allelic cluster (e.g. the centroid). A Bowtie2[34] reference database is finally constructed from the representative sequences.

<u>Read Alignment to Reference Database and Alignment Parsing</u>: Processed sequencing reads are next aligned against the database of representative sequences for homolog group alleles using Bowtie2 in "--very-sensitive-local" mode[34]. Because our database is currently based on individual genes (which are rather short), we found that aligning paired-end reads individually (as unpaired reads), increased sensitivity and did not significantly compromise specificity. This is similar to the approach for mapping used in MetaMLST[35]. After alignment, sorting and indexing of BAM files is performed using samtools[36]. Alignments for each homolog group are then processed and investigated using the pysam library in

Python[37]. An allele of a homolog group is considered potentially present if 90% of its sites are covered by at least one read, accounting for whether a read has lower than 30 base-quality and whether a site is a skipped region in the reference or corresponds to a deletion. Each alignment of a read to an allelic representative of a homolog group is then assessed as to whether it exhibits:

1. Alignment to multiple allelic representatives of the homolog group. Only alignments with the top/maximum alignment score of the read to any of the allelic representatives of the homolog group will be considered. This allows partitioning a read into one or more allelic representatives.

2. The read displays at least 95% identity to the reference allele sequence within the core alignment, where the core alignment is defined as the part of the alignment in between the first and last positions where reference and query sequences both have valid nucleotides (even if non-matching). If the core alignment length is >= 100 bp, 95% identity is required, while if it is shorter, >= 60 bp, 99% identity is required.

3. The aggregate indel length within the core alignment is < 5 bp. This allows for some leniency around small deletions and insertions, so as to not discard otherwise high-quality alignments. Reads with indels will be used for phasing or determining consensus alleles for homolog groups within metagenomes, but are not used as support for potentially novel SNVs reported.

If an alignment meets the above criteria, it is next assessed at each position for high base quality (>= 30 PHRED). If so, then the reference allele site is considered to be covered and the base of the read/query is noted. Further, because the reference allelic sequences were already aligned to each other and provided as the codon alignment inputs, we can translate the position of a site on the reference allele to a position in the codon multiple sequence alignment and ultimately gain a universal tally of base counts at particular sites of a homolog group. The synchronization of reference gene positions to codon alignment positions is a key feature of lsaBGC-DiscoVary.py which enables it to identify novel SNVs.

Final Assessment of Homolog Group Presence: As is often cautioned in gene-based metagenomic analysis, faulty alignment of reads belonging to the lineage of interest or from other taxa can lead to a misinterpretation of enzyme presence or association with the lineage of interest. To further filter out faulty alignments, we parse the codon alignments of each homolog group, featuring reference and representative allelic sequence, and mark regions along it which are particularly "gappy", >10% of sequences have no allelic residue, as troublesome, including +/- 50bp around the start and end of each region. These regions were observed to present problems as default gap opening and extension costs in the aligning algorithm can occasionally lead to faulty alignments. Based on a similar logic that alignment and alignment scores could behave irrationally when only part of reads should properly align to a reference, we also deem the first and last 50 bp of a MSA as "troublesome".

Additionally, we self-align the full predicted proteome of the genomes used to establish representative alleles for homolog groups with DIAMOND[17] to identify regions along the homolog group MSAs which are similar at high-identity to potential paralogs. The criteria for defining these regions matches our criteria for mapping reads to allelic sequences. Thus, alignments which are ≥20 residues long and exhibit ≥99% amino acid identity or ≥33 residues long and exhibit ≥90% amino acid identity for ≥5% of the initial sample set are marked as "troublesome" for accurate alignment of reads.

For each sequencing read set, each homolog group is next more thoroughly assessed for carriage in the context of the full BGC. Up to this stage, the criteria for consideration of a BGC homolog group as present is pretty lenient and simply requires 1X coverage at 90% of sites for one of the representative alleles of the homolog group. Here, we further refine this criteria to require 1X coverage at ≥90% of sites in the codon alignment of the homolog group which are deemed as non-troublesome to align. For homolog groups which meet this requirement, the median depth of the middle 80% of positions is computed. The median of these homolog group specific median depths is then calculated along with the

median absolute deviance. Because these homolog groups are expected to be co-located together in a BGC, potential variability in sequencing coverage across the genome, associated with active replication[38], should not result in a difference in coverage between homolog groups of the BGC. Based on this assumption, we next aim to identify and disregard homolog groups which are outliers in terms of their coverage (>2 median absolute deviances from the median of 80% trimmed median depths). These homolog groups will be difficult to gauge from raw metagenomics sequencing data as they will likely either lack enough coverage for resolved allele typing or have too much coverage and correspond to potentially multi-copy or common enzymes where undesired reads (from outside the focal BGC or lineage) are being aligned to the homolog group. Homolog groups are also disregarded if one of their predicted products contains mobile genetic element MGE suggestive keywords 'integrase' or 'transp'. In the most recent release of *lsa*BGC, we have updated the second keyword to ‘transpos’ to still allow for novel SNV detection on transporters.

If there are at least 5 homolog groups which are deemed present and not filtered by the above criteria, then BGC presence is assessed as a whole based on whether ≥70% of the core homolog groups are present (where the core homolog groups are those found in all BGCs from the initial *lsa*BGC processing/clustering analysis - e.g. the BGCs from high-quality genomes) or if just a single GCF specific homolog group is observed. Homolog groups are also disregarded if ≥5% of the initial set of the high-quality genomes used for initial lsaBGC analysis featured multiple copies of the homolog group (paralogs were common). Finally, a report file will be generated featuring only homolog groups deemed present (not filtered by above criteria) for samples which are regarded as featuring the BGC.

Consensus allele determination, allelic phasing, and phylogenetic visualization: For the set of retained homolog groups deemed as present within a sequencing readset for the focal GCF, the proportion of positions which are heterozygous are next computed and used to determine whether allelic phasing is needed or appropriate (current default ≥5% of sites along present homolog groups in the sequencing dataset need to be heterozygous to turn on phasing mode). If phasing mode is initiated and specified by users, DESMAN[33] is used to first determine the most likely number of strains and then phase the distinct haplotypes. If phasing mode is not initiated, then the consensus / majority-rule allele is selected for each site along the homolog group. Critically, the output from emission of the consensus sequence or multiple phased alleles for the homolog groups is not an independent sequence but rather an allele call for the sequencing / metagenomic read set at each position in the homolog group codon alignment. This allows us to avoid inferring faulty frameshifts in our sample-specific sequence(s) of homolog groups and allows direct incorporation into the codon alignment to build phylogenetic views. Additionally, we require that each base emitted / inferred has a minimum depth (current default is 5) and for the total depth at each position to be within a reasonable range of the median depth observed across all homolog groups regarded as present. For each inferred sequence, if an in-frame stop codon is observed, then downstream sites are automatically emitted as gaps.

The sequences inferred for each homolog group are brought into the context of the full codon alignments of reference alleles, after which filtering is performed to remove sequences which have gaps at more than 25% of non-"troublesome" sites of the alignments. Of the sequences retained after this filtering, individual sites are filtered to retain only those in which at most 10% of sequences have gaps / ambiguity. The resulting FASTA files are input into FastTree2[23] to infer a quick homolog group specific phylogeny which is then visualized in R and used to display the similarity of newly identified alleles / sequences to known / reference sequences.

Identification of novel SNVs: The cornerstone feature of lsaBGC-DiscoVary.py is its ability to search metagenomic / raw-read datasets and then assess whether they possess any potential novel SNVs not previously observed in the comprehensive set of known alleles gathered from all available assemblies for a taxa. Currently, only SNVs which are supported by at least 5 reads and not located in sites along the codon-alignment marked as "troublesome" are reported. Additionally, SNVs are not reported after the first in-frame stop codon observed from preliminary scanning. A comprehensive report of putatively

novel SNVs is produced for all sequencing samples and homolog groups. A subset of reads which are supportive of putatively novel SNVs (last column in the report) are written in gzipped FASTQ format for each sequencing / metagenomic sample. This allows users to quickly taxonomically profile and assess that reads in fact belong to the lineage of interest (see subsection below).

Further scrutinization of putative novel variants for lsaBGC-DiscoVary application to cutimycin in C. acnes *and the comprehensive set of GCFs for the* C. tuberculostearicum *species complex:* By default, lsaBGC-DiscoVary.py only considers reads aligning at 95% identity to reference gene sequences and avoids reporting novel variants on genes if either annotation or coverage (relative to other genes in the BGC context) suggests they might be MGEs. The program also automatically extracts the subset of reads supporting the existence of novel variants, which can then be further screened with high stringency filters using otherwise computationally expensive methods. This can involve mapping reads supporting putative novel SNVs to a representative database of whole-genomes to observe for higher quality alignments or checking whether reads are classified as a particular taxonomy by Kraken2 (v2.0.8-beta)[39]. As described previously[40], we used a customized database for Kraken2 analysis constructed from chromosomally complete bacterial, viral, archaeal, fungal, protozoan genomes as well as the human genome available on RefSeq, where plasmid sequences were distinguished to alleviate potential misclassifications of reads to a particular taxonomy.

We generally do not recommend screening variant-supporting reads for an appropriate taxonomic match with Kraken2 as it can generally lead to false negatives (on average only 92.8% of read-pairs per sample were taxonomically classified and misclassification can also occur; Fig. S14). For reads supporting putative novel variants within the cutimycin GCF of *C. acnes* or the six GCFs of the *C. tuberculostearicum* species complex, we required them to not align with a higher alignment score to a comprehensive database of all *Cutibacterium* or *Corynebacterium* genomes, respectively.

We further validated that reads supporting the existence of variants at conserved sites in the mycolic acid biosynthesis gene mapped best to the *C. tuberculostearicum* species and confirmed our suspicion that they could be misclassified by Kraken2 given the representation in the underlying database (Fig. S14). This *ad hoc* analysis was performed using paired-end information when mapping in Bowtie2 and accounting for concordant read pair alignments. While many reads supporting the existence of novel SNVs in the biosynthesis gene were classified as *C. acnes* by Kraken2, we found that none of the reads concordantly aligned to the comprehensive *Cutibacterium* genomes Bowtie 2 database whereas 85.07% concordantly aligned to the comprehensive *Corynebacterium* genomes Bowtie 2 database.

Note, the reasoning behind mapping SNV supporting reads to comprehensive genomic databases to purge false positive SNV calls, is that if a read truly aligns best to the BGC-associated homolog group where it was used to call a SNV, then it should at most generate a mapping to this comprehensive database of equal mapping quality. We align paired-end reads individually as was performed in lsaBGC-DiscoVary.py to have mapping scores be comparable with those from the original alignment. Furthermore, because our initial database within lsaBGC-DiscoVary.py consisted of allelic representatives of homolog groups, we realized that reads mapping to the start and end of genes could result in a lower mapping score than if a larger genomic context was provided. To ensure we do not remove such reads mapping to gene edges, scrutunizeNovelSNVSupport.py, the program we developed to perform Kraken 2 and genomic database alignment-based filtering of novel SNV reports from lsaBGC-DiscoVary.py, also compares the reference sequences when it finds a better Bowtie 2 mapping for SNV-supporting reads in genomic databases. If the reference sequence in the genomic database encompasses the reference sequence in the original alignment to the lsaBGC-DiscoVary.py homolog group database and the read was previously noted to map to a gene edge, then reads are still retained and SNV read support is not decremented. Five retained and supportive reads are required for an SNV to be featured in the filtered report produced by the program.

Overview of the *lsa*BGC-Easy workflow

Although not used in this study, recent versions of the *lsa*BGC suite introduced the *lsa*BGC-Easy workflow which enables automatically running most of the core programs in the suite for various bacterial taxa. As with the rest of the suite, it is designed to be applied to a single genus, species, or lineage and can gather accessions of genomes in NCBI associated with a particular taxonomic group based on classifications provided by GTDB. The workflow will then automatically download genomes using ncbi-genome-download (https://github.com/kblin/ncbi-genome-download) and perform gene-calling using prodigal. This is followed by GToTree species tree construction and determination of genome-to-genome similarity estimates using the core-genome alignment from GToTree. The core-genome alignment is also used for dereplication of genomes so that evolutionary statistics calculated downstream in lsaBGC-PopGene.py are not biased due to overrepresentation of particular strains or lineages in the NCBI Genomes database. Next, GECCO is used by default to perform BGC annotation; however, users can also specify to use antiSMASH or DeepBGC instead. GECCO is used as the default because it is lightweight and included in the *lsa*BGC conda environment as a dependency. For the latter two software, a task file will be produced with antiSMASH or DeepBGC commands for each genome which will need to be run by users in an environment with the respective programs available. After the commands are run, users can reinitiate *lsa*BGC-Easy using the same command they had issued originally to continue in the workflow. The following steps after BGC prediction are performing homolog group identification and annotation using lsaBGC-Ready.py. By default, only complete BGCs are selected and provided to lsaBGC-Ready.py because lsaBGC-AutoExpansion.py is used next to identify more comprehensive delineations of such fragmented BGCs anyway. This procedure leads to more accurate depictions of more common GCFs but results in a tradeoff that rare BGCs that only occur in a single genome and were fragmented could be missed and thus not investigated. Finally, lsaBGC-AutoAnalyze.py is used to run various *lsa*BGC core programs and generate a consolidated set of reports and visualizations.

A schematic and example commands for *lsa*BGC-Easy is provided on the *lsa*BGC GitHub wiki at: https://github.com/Kalan-Lab/lsaBGC/wiki/14.-lsaBGC-Easy-Tutorial. Various parameters controlling the workflow, such as inclusion of incomplete BGCs and skipping genome dereplication, are also available and described in the help function and on this Wiki page.

Updates to *lsa*BGC since release 1.0

Changes to *lsa*BGC since its initial release have primarily focused on simplifying usage of the suite through development of lsaBGC-Ready.py and lsaBGC-Easy.py. Analytical changes have mostly been minimal but include: (i) updated formatting of result files from the lsaBGC-AutoAnalyze.py workflow, (ii) changing how gaps are accounted for in calculating the Beta-RD statistic from codon-alignments, (iii) an adjustment to key-word searches used to prevent lsaBGC-DiscoVary from calling variants upon genes for which annotation suggests are MGEs, (iv) using MAGUS[41] in place of MAFFT[15] for protein alignment, to allow for better scalability, (v) introducing a more stringent requirement for homolog groups regarded as part of the protocore of BGC predictions by antiSMASH[2], (vi) use of refined hierarchical orthogrouping by OrthoFinder, and (vii) use of score or bitscore to assign new genes to the refined homolog groups during the expansion process for identification of new GCF instances. Incorporation of GToTree[5] also provided the opportunity to efficiently and more easily estimate expected similarities between genomes using protein alignments of single copy genes used by the software to construct phylogenies. As such, we have removed support for inferring Beta-RD using ANI or AAI between genomes. We have also added support for GECCO[42] and DeepBGC[43] predictions of BGCs. Finally, we have begun to introduce code to enable application of *lsa*BGC to fungi and plants but are still testing these functionalities.

Benchmarking Analyses for *lsa*BGC-AutoExpansion and *lsa*BGC-DiscoVary

M. luteus - *Benchmarking comprehensive antiSMASH vs. lsaBGC-AutoExpansion framework:* To benchmark the sensitivity and specificity of the *lsa*BGC framework, whereby we perform antiSMASH[2] based identification on a subset of complete genomes for a taxa and then identify homologous instances in draft quality genomes, against simply running antiSMASH on all the available genomes for the taxa, we performed two comparative analyses using *M. luteus* genomes (Fig. 2ab, S3, S4). Because lsaBGC-Expansion.py accounts for all homolog groups found in a GCF rather than being dependent on a smaller subset of BGC-associated domains, lsaBGC-Expansion.py should have increased sensitivity for detection of genomic regions belonging to a GCF compared to antiSMASH. For instance, if in truth a BGC exists across two separate scaffolds, but only one segment features all the core domains encoding for the secondary metabolite biosynthesis machinery, then a rule-based approach seeking specific domains might struggle with detection of the second segment consisting of only auxiliary cargo, even if some of those genes encode enzymes critical for the production of the final metabolite.

In the first experiment, to assess the sensitivity of lsaBGC-Expansion.py, we compared the final GCFs of two different lsaBGC analyses: (i) an expansion based analysis where we ran initial processing, antiSMASH based BGC identification, and GCF clustering for 14 high-quality "complete" / "chromosome" quality *M. luteus* assemblies and then perform expansion of the GCFs with 213 additional *M. luteus* draft genomic assemblies of lower quality, and (ii) a comprehensive clustering analysis where we ran initial processing, antiSMASH based BGC identification, and GCF clustering across all 227 genomic assemblies in consideration (Fig. S3a).

This comparative analysis essentially allowed us to assess how many instances (in this case contiguous segments) of a particular GCF are missed by the *lsa*BGC expansion-based framework (Fig. S3b). We found that almost all instances of BGCs found when running antiSMASH on the draft assemblies directly, were also identified through lsaBGC-AutoExpansion.py using BGCs from the 14 high-quality genomic assemblies as references (97.8%; 1206/1233). Nearly all the exceptions not detected in the expansion-based analysis (88.9%; 24 of 27) corresponded to rare GCFs which had no representatives in the high-quality assemblies. These cases demonstrate a significant limitation of the *lsa*BGC core framework in which BGCs are identified *de novo* in only a subset of genomic assemblies and is an important consideration for users.

The analysis also allowed us to assess how many instances of a particular GCF were only found by lsaBGC-AutoExpansion.py and not by running antiSMASH directly on draft genomes (Fig. S3c). Given that our approach is a taxa-focused analysis and utilizes all homolog groups associated with a GCF, not just those containing core BGC domains, it is not surprising that we were able to find substantially more instances of BGC segments in the draft assemblies using lsaBGC-AutoExpansion.py which were missed by antiSMASH. This is likely because of assembly fragmentation, as described in the example scenario above. In total, we found 689 new GCF instances only through lsaBGC-Expansion. Of these, most (87.1%, 600 of 689) correspond to true "expansions" (the GCF was detected as present in the samples in the comprehensive clustering analysis using antiSMASH, but additional segments containing auxiliary content on different scaffolds of the assembly were only detected by lsaBGC-AutoExpansion.py).

In the second benchmarking experiment, we again use the 14 completed *M. luteus* genomes available, but in this setup our aim was to compare how the *lsa*BGC approach compared to running *de novo* antiSMASH when applied to fragmented versions of the original genomic assemblies (Fig. S4a). To generate the fragmented assemblies, paired-end reads were first simulated from assemblies using ART[44] at 5X coverage and then re-assembled using Unicycler[45]. Five fragmented assemblies were created for each of the original 14 completed *M. luteus* genomes. On average fragmented assemblies had an N50 of 13,935 bp. This benchmarking setup allowed us to assess how many coding genes initially identified as part of distinct GCFs on complete genomes are re-identified after genomes are artificially fragmented.

Fragmented genomes were searched for BGCs using *de novo* antiSMASH and lsaBGC-AutoExpansion.py (run using GCF profiles based on the initial antiSMASH BGC predictions identified in the completed/unfragmented genomes). antiSMASH BGC predictions on fragmented genomes were mapped to GCFs determined from clustering antiSMASH BGC predictions on complete genomes via BLASTn analysis[46]. GCF classifications were transferred to BGCs from fragmented genomes if they aligned with at least 80% query coverage and 95% identity to a BGC from the completed genomes (99.1%; 331 of 334). lsaBGC-AutoExpansion.py automatically detects GCFs instances, thus such an analysis was not necessary (see section "High-throughput identification of homologous instances of GCFs in assemblies"). The 14 complete genomes were also searched for BGCs using lsaBGC-AutoExpansion.py in a reflexive analysis, since GCF profiles were based on antiSMASH BGC predictions on the same genomes.

As expected, based on the algorithms employed, we found that lsaBGC-AutoExpansion.py was generally able to identify larger BGCs relative to standard antiSMASH for both the fragmented and completed genomes (Fig. 2ab). Auxiliary genes that were excluded in some genomes as being part of the BGC but included in others by antiSMASH, due to variability in conservation and distance to core BGC domains, were systematically included in all BGC predictions by lsaBGC-AutoExpansion.py, thus "synchronizing" BGC boundaries for those belonging to the same GCF. This smoothing of BGC boundaries across homologous instances can be seen as another key feature of lsaBGC-AutoExpansion.py because it can allow more comprehensive and robust comparative genomics of GCFs downstream.

Also as expected, differences in the recovered length of BGCs between antiSMASH and lsaBGC-AutoExpansion.py were less apparent for shorter BGCs, because shorter BGCs are less likely to be fragmented in draft-quality assemblies (e.g. GCF-2 in Fig. 2ab). For a few singleton GCFs, which corresponded to rare BGCs found in only one genome (e.g. GCF-7, GCF-8, and GCF-9), lsaBGC-AutoExpansion.py predicted slightly shorter BGCs than what was originally detected by antiSMASH (Fig. 2ab). Investigations revealed that this was because antiSMASH BGCs included intergenic regions extending slightly past the boundary coding genes which lsaBGC-AutoExpansion.py was using to reidentify the BGCs. Another observation that was in alignment with expectations was that *de novo* antiSMASH run on fragmented genomes identified at most a single instance per GCF per fragmented genome. This is expected because when a BGC is fragmented across multiple scaffolds, the key domains used by antiSMASH for detection are likely only present on one scaffold (at least in *Micrococcus* where larger hybrid or multi-class BGCs are not common).

Coding genes were also matched between the initial completed genomes with their fragmented versions based on exact sequence mapping and by checking if ORFs from the fragmented genome were subsequences of ORFs from the complete genomes. lsaBGC-AutoExpansion.py was able to re-identify more of the initial coding genes associated with BGCs in complete genomes compared to rerunning antiSMASH *de novo* (Fig. 2ab, S4b). Rerunning antiSMASH on fragmented assemblies detected only 45.3% on average of the proteins it had originally associated with BGCs in complete genomes, while lsaBGC-AutoExpansion.py was able to recover 77.1% of them on average. However, lsaBGC-AutoExpansion.py did identify multiple instances of homolog groups which were not present in the initial BGC predictions on complete genomes (Fig. S1c, S4bc). As described in the previous paragraph, this is because lsaBGC-AutoExpansion.py used all antiSMASH predicted BGC instances from across the 14 genomes as references to identify GCFs in the fragmented genomes and thus increased the boundary of GCFs beyond what they might have initially been set to by the initial antiSMASH run on the complete (unfragmented) genomes (Fig. 2ab; S4bc).

Rapid determination of 63 GCFs in >15K Staphylococcus *genomes*: We were able to use lsaBGC-AutoExpansion.py to rapidly identify GCFs determined from complete *Staphylococcus* genomes in all ~15K *Staphylococcus* genomes represented in GTDB release R202. As expected, we found that fewer homolog groups in BGCs were detected for low quality assemblies (N50 < 10K; median of 105 homolog groups in BGCs per genome) as compared to high-quality assemblies (N50 > 100K; median of

164 homolog groups in BGCs per genome) (p=8.58E-36; two-sided Wilcoxon rank sum test). Furthermore, we found a lower percentage of homolog groups found together on the same BGC fragment with core biosynthetic machinery for low quality assemblies (N50 < 10K; median of 54%) as compared to high-quality assemblies (N50 > 100K; median of 100%) (p=8.64E-39; two-sided Wilcoxon rank sum test) (Fig. 2d). Note, the time taken to run lsaBGC-AutoExpansion.py on all draft-quality *Staphylococcus* genomes described in the main text is not inclusive of preliminary gene-calling using Prokka[1] performed via lsaBGC-AutoProcess.py.

Benchmarking lsaBGC-DiscoVary against assembly-based novel variant detection using M. luteus *single-isolate sequencing readsets:* The correspondence for novel single nucleotide variants (SNVs) reported by lsaBGC-DiscoVary.py was assessed using whole-genome sequencing readsets for single isolates and compared to SNV identification based on an assembly-based approach (Fig. S11). lsaBGC-AutoProcess.py was run on all complete instances of *M. luteus* genomes present in GTDB to identify BGCs which were then clustered into 9 GCFs using lsaBGC-Cluster.py. Afterwards, lsaBGC-AutoExpansion.py was used to search all remaining, draft-quality, genomes from the species for homologous instances of the 9 GCFs identified and lsaBGC-PopGene.py was run to generate codon-based alignments for homolog groups associated with each GCF. We then used the sequencing reads from 132 *M. luteus* isolates sequenced by our lab, and absent in the current GTDB release, to run lsaBGC-DiscoVary.py and identify novel variants not previously observed in available genomes for the species. Because we had constructed draft assemblies for these same samples, we also identified GCF and homolog group instances in each assembly using lsaBGC-AutoExpansion.py. MAFFT[15] was then used to incorporate the homolog group sequences identified in the draft assemblies into the codon alignments used for lsaBGC-DiscoVary.py analysis and the expanded codon alignments were subsequently parsed and assessed for novel SNVs. We found a high concordance between the lsaBGC-DiscoVary.py and assembly-based approaches for novel SNV detection, with 1788 of 1798 (99.5%) novel SNVs reported by DiscoVary also being found by the assembly based approach and 1788 of 1858 (96.3%) novel SNVs found by the assembly based approach also being reported by lsaBGC-DiscoVary (Table S11). Of the 69 novel SNVs only found by the assembly based approach, 30 were identified by lsaBGC-DiscoVary.py but not reported due to alleles exhibiting more or less coverage than expected given the median coverage of the BGC. Among the remaining 39 novel SNVs, manual examination of a common SNV (homolog group OG0001039 - position 65 in the codon alignments; found in 15 samples), revealed that it involved a cytosine allele within a lengthy stretch of 21 C/Gs, which could lead to less confident read alignment in lsaBGC-DiscoVary.py. Of the nine novel SNVs found by lsaBGC-DiscoVary, six correspond to minor alleles, explaining why they were not represented in sample assemblies.

Determination of whether novel SNVs detected for BGCs of the C. tuberculostearicum *species complex would be identified in metagenomic assemblies:* Because the *C. tuberculostearicum* species complex can be found at low abundance at certain body sites within individuals, we wanted to determine whether the 34,545 novel SNVs found in BGC contexts of the species complex, after filtering SNVs where reads mostly map with higher scores to other regions of *Corynebacterium* genomes (as described in the previous subsection), would also be detected through metagenomic assembly. MEGAHIT[47] assembly was performed individually for all metagenomic readsets using default options and filtered to only retain 2 kb or longer contigs. Sample specific metagenomic assemblies were then combined into one consolidated FASTA file and used to construct a Bowtie 2 database[34]. No pooling of samples for metagenomic assembly or subsequent dereplication was performed to ensure strain-specific variability is retained. After assessing the presence of putative novel SNVs and filtering those where supportive reads are mostly mapped to alternate regions in *Corynebacterium* genomes at higher scores (as described in the *Materials and Methods*), we performed similar investigations through Bowtie 2 mapping of reads supporting novel SNV presence to the concatenated database of contigs from individual metagenomes. Exact mappings of SNV-supporting reads to contigs were identified and used to assess whether novel SNVs are represented in metagenomic assemblies. Of the 34,545 novel SNVs deemed as reliable after mapping to the comprehensive database of *Corynebacterium* genomes, we found that 22,886 would also

be identified from metagenomic assembly. Thus, 11,659 SNVs detected by lsaBGC-DiscoVary.py would be missed by metagenomic assembly and are not represented in contigs of considerable length. This is expected to be an underestimate as a concatenated database of metagenomic contigs from multiple samples was used. We additionally tested mapping SNV supporting reads to only a subset of the metagenomic contigs which we deemed as belonging to *Corynebacterium.* These contigs were identified by BLASTn analysis[46] of the metagenomic contigs to the comprehensive *Corynebacterium* genomics database used to filter putative novel SNVs in the previous subsection. Contigs with HSPs with query coverage greater than 25% and sequence identity greater than 85% or query coverage greater than 70% and sequence identity greater than 70% to one of the known *Corynebacterium* genomes were classified as *Corynebacterium*. Of the 556,030 total concatenated metagenomic contigs, 91,713 were classified as *Corynebacterium*. However, using this subset of contigs instead of the full metagenomic assembly database resulted in a minor increase in the number of SNVs which would be undetected by metagenomic assembly (11,763 instead of 11,659; an increase of 0.9%), suggesting that reads supporting novel SNVs detected by lsaBGC-DiscoVary.py were >99% *Corynebacterium.*

Additional Details on Analyses in Study

Parameters and thresholds used for clustering GCFs and subsequent annotation

lsaBGC-Cluster.py was used to cluster BGCs into GCFs within this study. For consistency and simplicity, we ran both the genus-level and species-level analyses using identical parameters for lsaBGC-Cluster.py. The MCL inflation parameter was set to 4.0, the minimal syntenic similarity threshold was set to 0.7, and the Jaccard similarity of homolog groups shared vs. observed in union between two BGCs threshold was set to 20.

For biosynthetic class designations of GCFs in this study, we regarded GCFs as a single class/type if at least 90% of BGC instances from complete genomes were predicted by antiSMASH as encoding for the class; otherwise, the GCF was regarded as a hybrid.

Ancestral inference of gene cluster family carriage

We performed ancestral inference of GCF carriage to predict vertical descent of GCFs within the skin-associated phylogenetic clades of *Staphylococcus* and *Corynebacterium* (Fig. S5ab)[48]. GCF carriage across genomes was encoded as a binary trait and AncestralGeneRator (https://github.com/broadinstitute/AncestralGeneRator) was used to perform ancestral state reconstruction to infer carriage for inner-nodes of the *Corynebacterium* and *Staphylococcus* ribosomal phylogenies. Briefly, PAUP (v4b)[49] was used to perform maximum parsimony with the ACCTRAN algorithm, setting gain and loss costs to 10 and 5, respectively. Afterwards, result files from AncestralGeneRator were further processed and were visualized using iTol[22]. This analysis revealed that four GCFs were ancestral to the *S. epidermidis/aureus* clade (predicted siderophore, terpene/T3PKS, cyclic lactone autoinducer, and hserlactone) and five GCFs were ancestral to the *C. tuberculostearicum* species complex (predicted siderophore, terpene, T1PKS, NAPAA, and NAPAA/betalactone).

Identification of homolog groups associated with skin-residing species of *Staphylococcus* and *Corynebacterium*

We developed a script called crawlingFisher.py, provided in the *lsa*BGC suite, which tests for enrichment or depletion of homolog group presence amongst genomes in focal clades using Fisher's exact test. Provided with a phylogenetic tree and matrix specifying the presence of homolog groups across genomes, the program automatically performs such tests for each homolog group at each inner-node of the phylogeny. Multiple testing correction was performed comprehensively at the end using Benjamini-Hochberg false discovery rate and cases were considered statistically significant if the adjusted p-value was less than 0.05. Results were further filtered to retain only homolog groups which were present in at

least 80% of genomes under the focal node and at most 20% of other genomes or, alternatively, at most 20% of genomes under the focal node and at least 80% of other genomes. Finally, only results pertaining to focal nodes where at least 80% of genomes were classified as skin-associated were reported (Fig. S5ab, Table S5).

Inspection of additional GCFs from *Staphylococcus* and *Corynebacterium* with high Beta-RD values

Investigation into High Beta-RD of lugdunin encoding NRPS GCF-46 in Staphylococcus: Among staphylococcal GCFs, GCF-46 depicted the highest posterior Beta-RD distribution (Fig. S5c). This GCF encodes for the lugdunin NRPS, which was determined to be an antibiotic produced by *S. lugdunensis* which is active against *S. aureus*[50]. The ubiquity of this GCF being regarded as present in multiple staphylococcal species by lsaBGC-AutoExpansion.py was unexpected; however, further investigation revealed a rare instance of a small insertion-sequence element, which is ubiquitous across *Staphylococcus*, within the protocore region of the lugdunin BGC, thus resulting in lsaBGC-AutoExpansion.py over-classifying the presence of the GCF as it assumes the genes within the element are highly BGC-associated. Although we suspect such cases to be rare, we have since introduced more stringent requirements for defining homolog groups as part of the protocore of BGCs predicted by antiSMASH in more recent releases of *lsa*BGC.

Investigation into Corynebacterium *GCF with the second highest Beta-RD value*: In addition to *Corynebacterium* NRPS encoding GCF-50, the NRPS encoding GCF-9 exhibited a similarly high posterior Beta-RD distribution (Fig. S4d). GCF-9 was found to also be present in multiple species on the skin and was most prevalent in the well-known skin pathogen *C. diphtheria*[51]. This GCF exhibited a similar scenario to GCF-50, in which it featured a highly conserved region with NRP synthase(s) flanked by MGEs in addition to a nearby phage and toxin/antitoxin system. GCF-9 was not further investigated because the NRP synthase (OG0002384) within GCF-9 could be found in multiple copies within certain genomes. To address such limitations, in version 1.3 of *lsa*BGC, we have switched the default settings to use more resolute orthogroup classifications by OrthoFinder (hierarchical orthogroups), which we found are able to better partition paralogous groups of NRP synthases.

Assessment of hybrid genomic assemblies constructed for *S. epidermidis* LK1136 and *S. warneri* LK413

We chose to investigate staphyloxanthin production in *S. epidermidis* and *S. warneri* because they are *Staphylococcus* species commonly isolated from skin. Specifically, the former species *S. epidermidis*, is the most abundant staphylococcal species on skin and the latter species, *S. warneri*, featured two distinct staphyloxanthin encoding GCFs. For the *S. epidermidis* LK1136 hybrid ONT and Illumina genome assembly, we found that the isolate featured eight plasmids, including a 225 kb mega-plasmid (Table S7b). All eight plasmids were regarded as circularized; however, the chromosome was not. For the *S. warneri* LK413 hybrid genome assembly, we found that the isolate featured three plasmids, of which two were circularized (Table S7b). The chromosome for this isolate was circularized and regarded as complete. The incomplete scaffold in *S. warneri* was found to feature staphyloxanthin encoding GCF-6 and to validate it represented a plasmid, we used the BLASTn[46] to NCBI's nt analysis performed in GAEMR(https://software.broadinstitute.org/software/gaemr/) to confirm that all reported alignments were to *Staphylococcus* plasmid sequences. Additionally, this scaffold featured a slightly elevated coverage, ~3X greater than the chromosome.

Designation of species belonging to *Mammaliicoccus*

The *Staphylococcus_A* genus designation in GTDB R202 was largely concordant with the recent reclassification of five species from *Staphylococcus* to *Mammaliicoccus*[52]. In the latest GTDB R207 release, this genus has now been reclassified to *Mammaliicoccus*. The recent update to taxonomic names also includes the reclassification of species *S. pasteuri_A* to *M. fleurettii*. Notably, we do not regard *S.*

schleiferi as part of *Mammaliicoccus* because it is categorized as *Staphylococcus* by GTDB (both R202 and R207) and groups within the *Staphylococcus* genus in our ribosomal protein-based maximum-likelihood phylogeny. Similar to our assessment, the reclassification of this species to *Mammaliicoccus* was recently questioned[53].

*lsa*BGC-DiscoVary based assessment of cutimycin presence in skin metagenomes

For investigations of the presence of cutimycin from *C. acnes* in skin metagenomes, we manually specified the core homolog groups involved in the thiopeptides biosynthesis[54] to increase sensitivity through an optional setting in lsaBGC-DiscoVary.py.

*lsa*BGC-DiscoVary based analyses of BGCs from the *C. tuberculostearicum* species complex

For the following analyses, we used 5,802 instances of 2,343 unique novel SNVs found across 66 homolog groups which were core or adjacent to core biosynthesis machinery of BGCs from the *C. tuberculostearicum* species complex. A total of 68 context-distinct homolog groups were determined after partitioning multi-GCF occurring homolog groups based on their GCF context (OG0000094 and OG0000100 were each found in two separate GFCs and thus each was partitioned as two separate homolog groups). The 2,343 novel SNVs had site and allele-specific coverage values less than two median absolute deviations from the median coverage for the entire BGC.

Comparing SNVs shared across body sites and subjects: To comprehensively assess whether novel SNVs were shared more frequently for microbiomes of the same body site or from the same individual, we performed a pairwise, multi-iteration resampling analysis. For each pair of metagenomes, where each sample had at least 30 novel SNVs, we calculated the average Jaccard similarity across 1,000 simulations in which we randomly drew sets of 30 SNVs from each metagenomic sample to control for sequencing depth. Pairs of metagenomes were classified as one of three categories: "different body site, different participant", "different body site, same participant", and "same body site, different participant" (Fig. S13e). A two-sided Wilcoxon rank sum test for differences between the three distributions of average Jaccard similarities revealed that there was a statistically significant difference between "different body site, same participant' and "same body site, different participant" (p=6.01E-3). Of greater relevance, there were statistically strong significant differences between the "different body site, different participant" distribution and the two other distributions: "different body site, same participant" and "same body site, different participant" (p=3.67E-09 and p=1.39E-116, respectively). Thus, pairs of metagenomes were more likely to share novel SNVs if they were either from the same body site or same participant compared to if they were from different participants and different body sites. Two metagenomic samples for participant S002 were not used because they corresponded to a resampling of two of their body sites and we did not have enough of such samples to pursue a temporal analysis. We assessed this testing using different considerations/cutoffs for: (i) the number of novel SNVs needed for a metagenome to be considered in the analysis and (ii) whether or not to account for singleton SNVs. In all cases, statistical testing for differences between distributions of pairwise metagenome categories yielded similar conclusions.

Assessing trends for novel SNV predicted synonymous to non-synonymous rate with metagenome ubiquity and site conservation: Using reports from lsaBGC-DiscoVary.py, we performed a systematic prediction of whether novel SNVs identified on the 68 context-distinct homolog groups nearby or overlapping with protocore regions of BGCs in *C. tuberculostearicum* corresponded to synonymous or non-synonymous substitutions. As described in other sections, SNV alleles were compared to corresponding alleles observed at the site on the reference gene sequences which SNVs were called upon. We aimed to check whether the rate of synonymous to non-synonymous novel SNVs showed a relationship to: (i) how common SNVs were across metagenomic samples and (ii) conservation levels across protein sequences.

Novel SNV predicted synonymous to non-synonymous rate increases with SNV metagenome ubiquity amongst metagenomic samples: The occurrence of novel SNVs, a novel allele observed at a specific site in the multiple sequence codon alignment of a particular homolog group, was tabulated across metagenomic samples. While novel SNVs found in a single metagenome were only 2.4X as likely to correspond to a synonymous change as opposed to a non-synonymous change, more prevalent novel SNVs, found in ten or more samples, were 8.4X more likely to correspond to a synonymous change (Fig. S13d).

Novel SNV predicted synonymous to non-synonymous rate increases at conserved sites in protein sequences: We searched RefSeq's bacterial NR database for remote homologs of the 68 context-distinct homolog groups within or adjacent to protocore regions of BGCs. The top 20 homologs which belonged to classified bacterial species outside of the *Corynebacterium* genus or *Corynebacteriales* family were identified using hmmsearch from HMMER3 with profile-HMMs gathered from *lsa*BGC-Expansion for each homolog group and an E-value threshold of 1E-20 (Table S12). Of the 2,343 unique novel SNVs identified in protocore or protocore adjacent homolog groups of BGCs from the *C. tuberculostearicum* species complex, 2,244 were found in 34 context-distinct homolog groups with conservation information calculated from alignments featuring distantly related homologs from diverse bacteria. The sequences of representative homologs were added to protein alignments for homolog groups in *lsa*BGC-PopGene results using the '--add' option in MAFFT[15]. Afterwards, we used the alignments for scoring protein sequence conservation with Jensen-Shannon divergence[55]. Conservation scores were used to determine conservation percentiles of sites along protein alignments for individual homolog groups.

Rates of predicted synonymous to non-synonymous novel SNVs were calculated for each conservation percentile across all homolog groups. Novel SNVs found in the top five percentile of conserved sites for a homolog group were approximately 9.8X more likely to correspond to a synonymous change as opposed to a non-synonymous change (Fig. 6b). In contrast, the bottom 20% of conserved sites (the least conserved sites), were only 1.7X as likely to represent non-synonymous substitutions as synonymous substitutions. Further, the number of novel SNVs, either synonymous or non-synonymous observed at conserved sites, amongst the top 20% of conserved sites was 412, and lower than the number of novel SNVs found in the 20-40 (n=459), 40-60 (n=490), and 60-80 (n=493). Only 388 novel SNVs were found in the 80-100 percentile ranges of conserved sites and we suspect that this decrease is due to read alignments to such regions becoming more challenging at the thresholds required by lsaBGC-DiscoVary.py.

For the three SNVs predicted to result in non-synonymous differences and in the top five percentile of conserved sites along the mycolic acid PKS, we inspected their codon contexts to check that there were no additional variants which, in aggregate, might result in a synonymous change (Fig. 6c; Table S13).

Supplementary Text References

1. Seemann, T. Prokka: rapid prokaryotic genome annotation. *Bioinformatics* **30**, 2068–2069 (2014).

2. Blin, K. *et al.* antiSMASH 6.0: improving cluster detection and comparison capabilities. *Nucleic Acids Res.* **49**, W29–W35 (2021).

3. Emms, D. M. & Kelly, S. OrthoFinder: phylogenetic orthology inference for comparative genomics. *Genome Biol.* **20**, 238 (2019).

4. Navarro-Muñoz, J. C. *et al.* A computational framework to explore large-scale biosynthetic diversity. *Nat. Chem. Biol.* **16**, 60–68 (2020).
5. Lee, M. D. GToTree: a user-friendly workflow for phylogenomics. *Bioinformatics* **35**, 4162–4164 (2019).
6. Hyatt, D. *et al.* Prodigal: prokaryotic gene recognition and translation initiation site identification. *BMC Bioinformatics* **11**, 119 (2010).
7. Aramaki, T. *et al.* KofamKOALA: KEGG Ortholog assignment based on profile HMM and adaptive score threshold. *Bioinformatics* **36**, 2251–2252 (2019).
8. Tatusova, T. *et al.* NCBI prokaryotic genome annotation pipeline. *Nucleic Acids Res.* **44**, 6614–6624 (2016).
9. Parks, D. *CompareM: A toolbox for comparative genomics*. (Github).
10. Jain, C., Rodriguez-R, L. M., Phillippy, A. M., Konstantinidis, K. T. & Aluru, S. High throughput ANI analysis of 90K prokaryotic genomes reveals clear species boundaries. *Nat. Commun.* **9**, 1–8 (2018).
11. van Dongen, S. & Abreu-Goodger, C. Using MCL to extract clusters from networks. *Methods Mol. Biol.* **804**, 281–295 (2012).
12. Steinke, K., Mohite, O. S., Weber, T. & Kovács, Á. T. Phylogenetic Distribution of Secondary Metabolites in the Bacillus subtilis Species Complex. *mSystems* **6**, (2021).
13. Melnyk, R. A., Hossain, S. S. & Haney, C. H. Convergent gain and loss of genomic islands drive lifestyle changes in plant-associated Pseudomonas. *ISME J.* **13**, 1575–1588 (2019).
14. Cantalapiedra, C. P., Hernández-Plaza, A., Letunic, I., Bork, P. & Huerta-Cepas, J. eggNOG-mapper v2: Functional Annotation, Orthology Assignments, and Domain Prediction at the Metagenomic Scale. *Mol. Biol. Evol.* **38**, 5825–5829 (2021).

15. Katoh, K. & Standley, D. M. MAFFT multiple sequence alignment software version 7: improvements in performance and usability. *Mol. Biol. Evol.* **30**, 772–780 (2013).

16. Eddy, S. R. Accelerated Profile HMM Searches. *PLoS Comput. Biol.* **7**, e1002195 (2011).

17. Buchfink, B., Xie, C. & Huson, D. H. Fast and sensitive protein alignment using DIAMOND. *Nat. Methods* **12**, 59–60 (2014).

18. Schreiber, J. Pomegranate: fast and flexible probabilistic modeling in python. *J. Mach. Learn. Res.* (2017).

19. Cimermancic, P. *et al.* Insights into secondary metabolism from a global analysis of prokaryotic biosynthetic gene clusters. *Cell* **158**, 412–421 (2014).

20. Wickham, H. *ggplot2: Elegant Graphics for Data Analysis*. (Springer, New York, NY, 2009).

21. Yu, G., Smith, D. K., Zhu, H., Guan, Y. & Lam, T. T.-Y. Ggtree : An r package for visualization and annotation of phylogenetic trees with their covariates and other associated data. *Methods Ecol. Evol.* **8**, 28–36 (2017).

22. Letunic, I. & Bork, P. Interactive Tree Of Life (iTOL) v4: recent updates and new developments. *Nucleic Acids Res.* **47**, W256–W259 (2019).

23. Price, M. N., Dehal, P. S. & Arkin, A. P. FastTree 2--approximately maximum-likelihood trees for large alignments. *PLoS One* **5**, e9490 (2010).

24. Suyama, M., Torrents, D. & Bork, P. PAL2NAL: robust conversion of protein sequence alignments into the corresponding codon alignments. *Nucleic Acids Res.* **34**, W609-12 (2006).

25. Whalley, T. *LICENSE at master · WhalleyT/tajima*. (Github).

26. Tajima, F. Statistical method for testing the neutral mutation hypothesis by DNA polymorphism. *Genetics* **123**, 585–595 (1989).

27. Cock, P. J. A. *et al.* Biopython: freely available Python tools for computational molecular biology and bioinformatics. *Bioinformatics* **25**, 1422–1423 (2009).

28. Nei, M. & Gojobori, T. Simple methods for estimating the numbers of synonymous and nonsynonymous nucleotide substitutions. *Mol. Biol. Evol.* **3**, 418–426 (1986).

29. Chase, A. B., Sweeney, D., Muskat, M. N., Guillén-Matus, D. G. & Jensen, P. R. Vertical Inheritance Facilitates Interspecies Diversification in Biosynthetic Gene Clusters and Specialized Metabolites. *MBio* **12**, e0270021 (2021).

30. Waack, S. *et al.* Score-based prediction of genomic islands in prokaryotic genomes using hidden Markov models. *BMC Bioinformatics* **7**, 142 (2006).

31. Olm, M. R. *et al.* inStrain profiles population microdiversity from metagenomic data and sensitively detects shared microbial strains. *Nat. Biotechnol.* (2021) doi:10.1038/s41587-020-00797-0.

32. Gregory, A. C. *et al.* MetaPop: a pipeline for macro- and microdiversity analyses and visualization of microbial and viral metagenome-derived populations. *Microbiome* **10**, 49 (2022).

33. Quince, C. *et al.* DESMAN: a new tool for de novo extraction of strains from metagenomes. *Genome Biol.* **18**, 1–22 (2017).

34. Langmead, B. & Salzberg, S. L. Fast gapped-read alignment with Bowtie 2. *Nat. Methods* **9**, 357–359 (2012).

35. Zolfo, M., Tett, A., Jousson, O., Donati, C. & Segata, N. MetaMLST: multi-locus strain-level bacterial typing from metagenomic samples. *Nucleic Acids Res.* **45**, e7 (2017).

36. Li, H. *et al.* The Sequence Alignment/Map format and SAMtools. *Bioinformatics* **25**, 2078–2079 (2009).

37. Gilman, P. *et al.* *PySAM (Python Wrapper for System Advisor Model "SAM").* https://www.osti.gov/servlets/purl/1559931 (2019) doi:10.11578/dc.20190903.1.

38. Bremer, H. & Churchward, G. An examination of the Cooper-Helmstetter theory of DNA replication in bacteria and its underlying assumptions. *J. Theor. Biol.* **69**, 645–654 (1977).

39. Wood, D. E., Lu, J. & Langmead, B. Improved metagenomic analysis with Kraken 2. *Genome Biol.* **20**, 1–13 (2019).

40. Swaney, M. H., Sandstrom, S. & Kalan, L. R. Cobamide sharing drives skin microbiome dynamics. *bioRxiv* 2020.12.02.407395 (2021) doi:10.1101/2020.12.02.407395.

41. Smirnov, V. Recursive MAGUS: Scalable and accurate multiple sequence alignment. *PLoS Comput. Biol.* **17**, e1008950 (2021).

42. Carroll, L. M. *et al.* Accurate de novo identification of biosynthetic gene clusters with GECCO. *bioRxiv* 2021.05.03.442509 (2021) doi:10.1101/2021.05.03.442509.

43. Hannigan, G. D. *et al.* A deep learning genome-mining strategy for biosynthetic gene cluster prediction. *Nucleic Acids Res.* **47**, e110 (2019).

44. Huang, W., Li, L., Myers, J. R. & Marth, G. T. ART: a next-generation sequencing read simulator. *Bioinformatics* **28**, 593–594 (2012).

45. Wick, R. R., Judd, L. M., Gorrie, C. L. & Holt, K. E. Unicycler: Resolving bacterial genome assemblies from short and long sequencing reads. *PLoS Comput. Biol.* **13**, e1005595 (2017).

46. Camacho, C. *et al.* BLAST+: architecture and applications. *BMC Bioinformatics* **10**, 421 (2009).

47. Li, D., Liu, C.-M., Luo, R., Sadakane, K. & Lam, T.-W. MEGAHIT: an ultra-fast single-node solution for large and complex metagenomics assembly via succinct de Bruijn graph. *Bioinformatics* **31**, 1674–1676 (2015).

48. van Dijk, L. R. *et al.* StrainGE: a toolkit to track and characterize low-abundance strains in complex microbial communities. *Genome Biol.* **23**, 74 (2022).

49. Swofford, D. L. PAUP: phylogenetic analysis using parsimony. *Mac Version 3. 1. 1.(Computer program and manual).* (1993).

50. Zipperer, A. *et al.* Human commensals producing a novel antibiotic impair pathogen colonization. *Nature* **535**, 511–516 (2016).

51. Hadfield, T. L., McEvoy, P., Polotsky, Y., Tzinserling, V. A. & Yakovlev, A. A. The pathology of diphtheria. *J. Infect. Dis.* **181 Suppl 1**, S116-20 (2000).

52. Madhaiyan, M., Wirth, J. S. & Saravanan, V. S. Phylogenomic analyses of the Staphylococcaceae family suggest the reclassification of five species within the genus Staphylococcus as heterotypic synonyms, the promotion of five subspecies to novel species, the taxonomic reassignment of five Staphylococcus species to Mammaliicoccus gen. nov., and the formal assignment of Nosocomiicoccus to the family Staphylococcaceae. *Int. J. Syst. Evol. Microbiol.* **70**, 5926–5936 (2020).

53. Kania, S. A. Reclassification of Staphylococcus schleiferi by Madhaiyan et al. lacks key supporting data. *Int. J. Syst. Evol. Microbiol.* **72**, (2022).

54. Claesen, J. *et al.* A Cutibacterium acnes antibiotic modulates human skin microbiota composition in hair follicles. *Sci. Transl. Med.* **12**, (2020).

55. Capra, J. A. & Singh, M. Predicting functionally important residues from sequence conservation. *Bioinformatics* **23**, 1875–1882 (2007).

Supplementary Figures

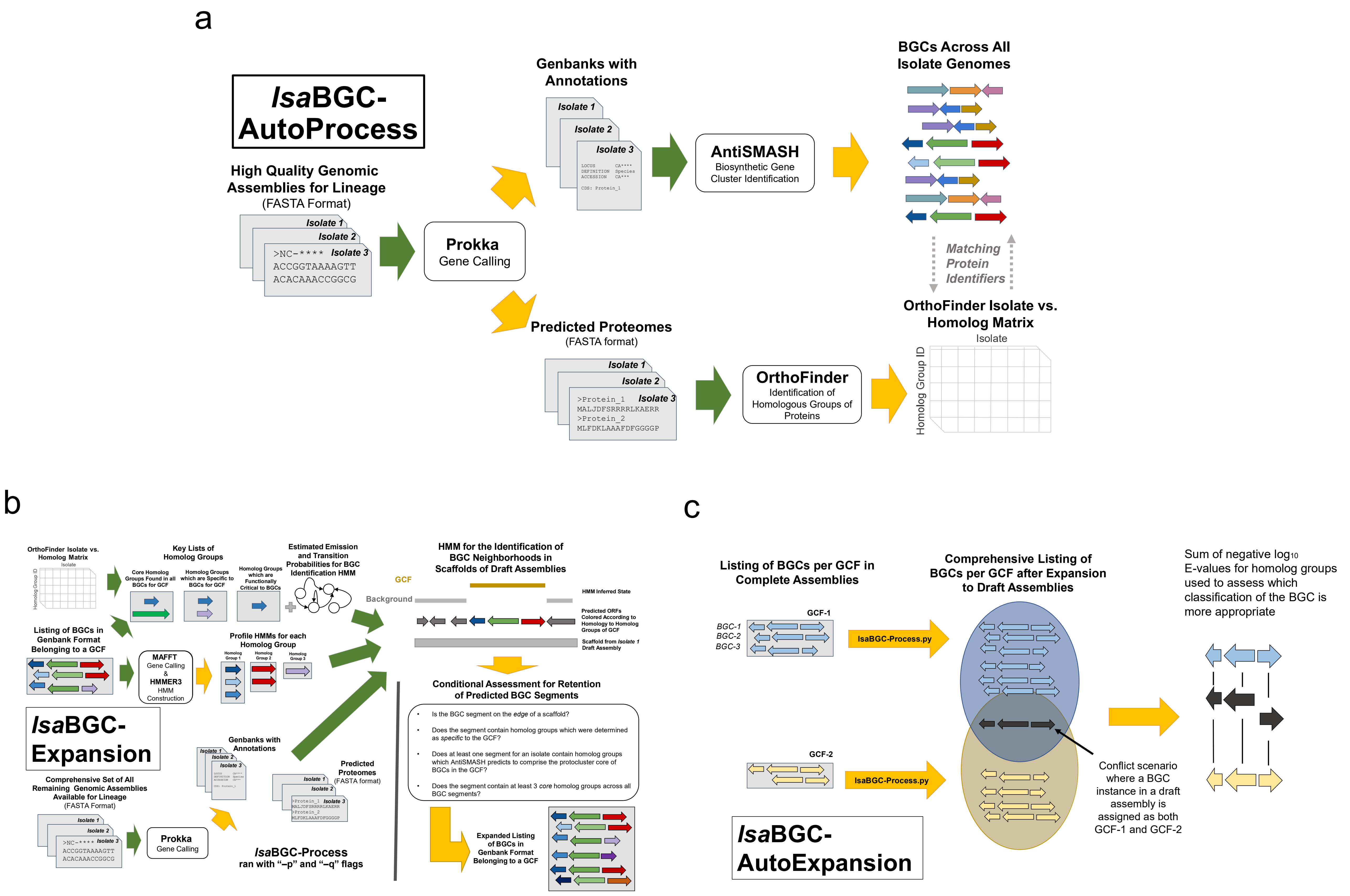

Figure S1: Schematics of select *Isa*BGC programs and workflows. a) A schematic of the *Isa*BGC-AutoProcess workflow which generates the inputs required for IsaBGC analyses. b) An overview of the algorithm behind *Isa*BGC-Expansion used to identify homologous instances of GCFs in a sensitive and efficient manner from potentially draft-quality assemblies. c) A schematic of the *Isa*BGC-AutoExpansion workflow which automatically runs *Isa*BGC-Expansion for each GCF and then resolves conflicts of overlap and consolidates results.

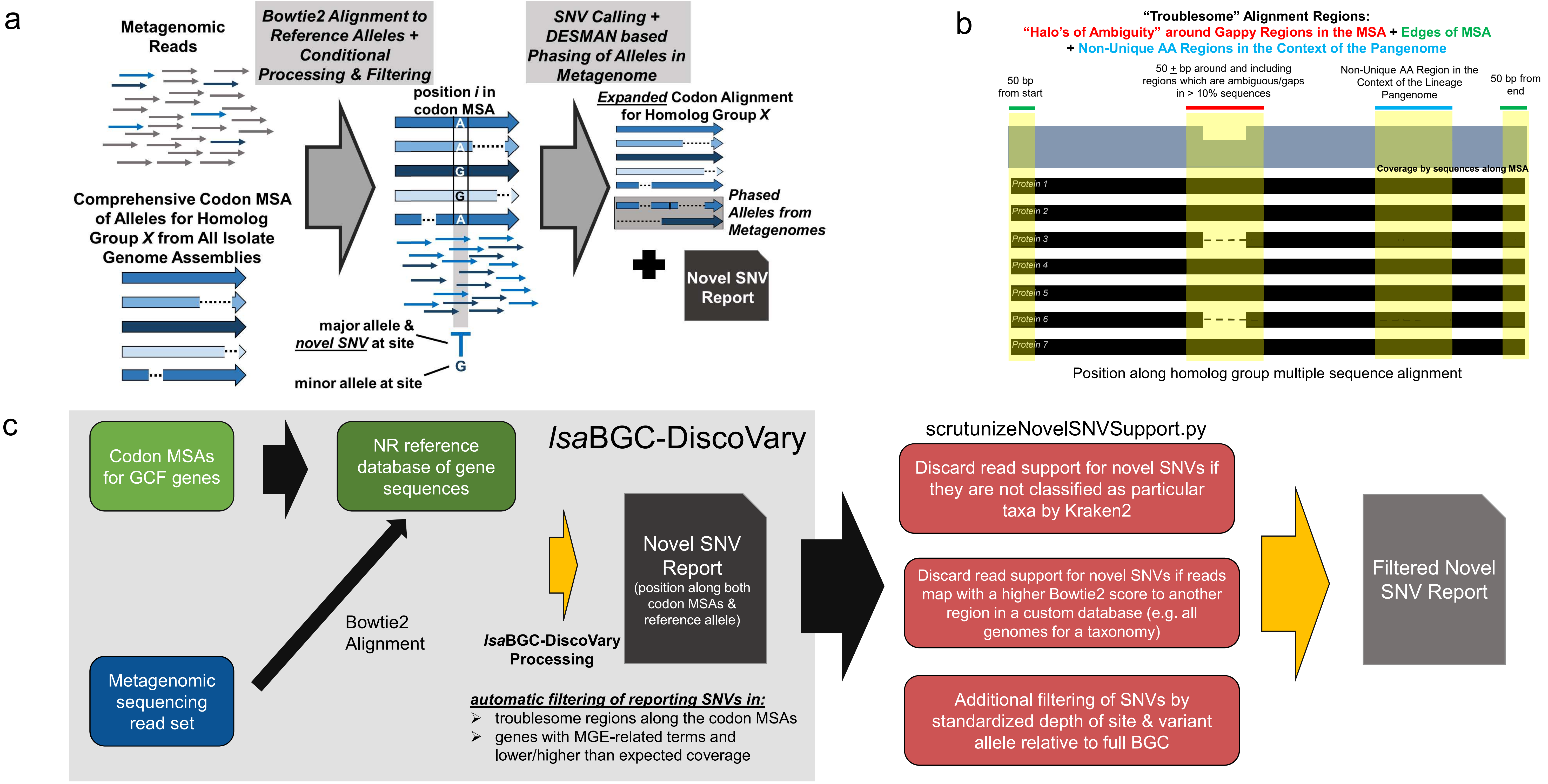

Figure S2: An overview of *lsa*BGC-DiscoVary. a) An overview of the *lsa*BGC-DiscoVary algorithm is shown. Codon alignments for each BGC homolog group, as constructed by *lsa*BGC-PopGene, are used to dereplicate and select representative alleles for mapping metagenomic readsets with Bowtie2. Afterwards, alignments are processed and used to identify whether homolog groups are represented in metagenomic samples. SNV sites along individual representative genes for homolog groups are mapped to comprehensive codon alignments and used to assess whether the allele represented by the SNV has previously been observed at the codon alignment position. Optional phasing of multiple alleles for a homolog group within a lineage or taxa can also be performed using DESMAN. **b**) Novel SNVs are not reported if they are within specific regions along codon alignments, including regions which are towards the beginning or end of the alignment, regions which are highly redundant, and regions where >10% of gene instances have deletions or lack sequence. **c**) An overview of *lsa*BGC-DiscoVary for determining putative novel SNVs which can then be further filtered using exhaustive methods to assert that reads supporting their presence do not map better to other taxa or genomic regions.

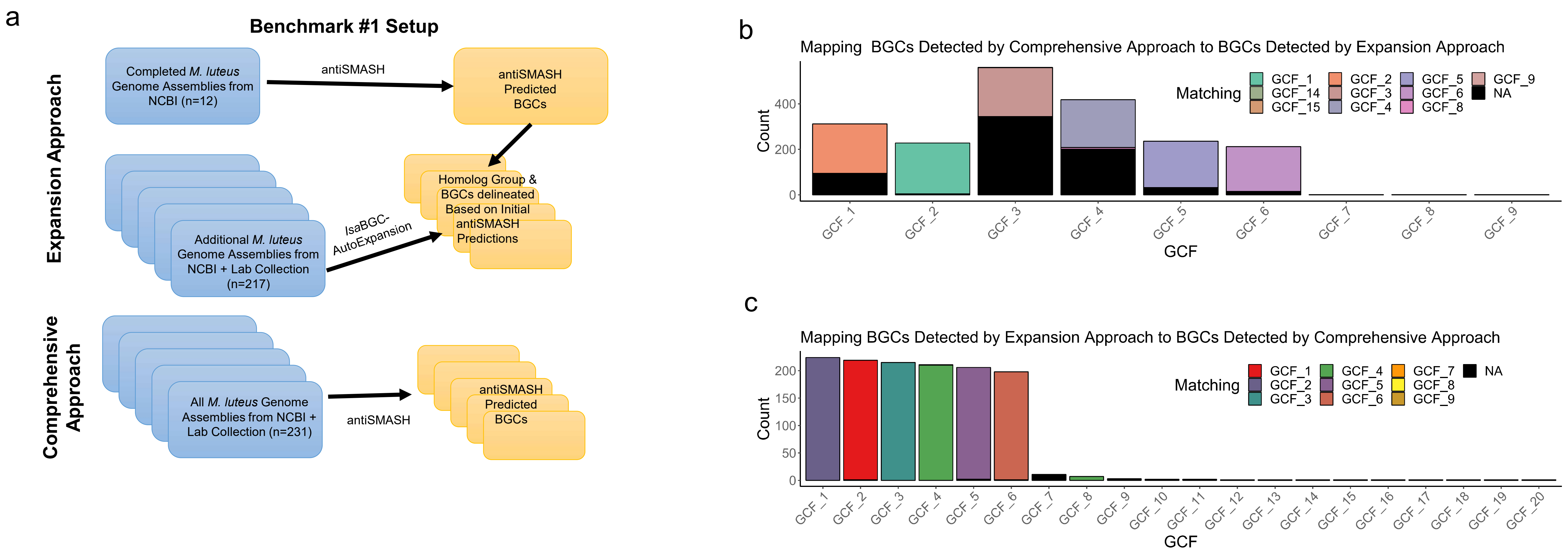

Figure S3: Benchmarking *Isa*BGC-AutoExpansion using actual draft-quality *M. luteus* assemblies. a) An overview of the first benchmarking experiment is shown comparing the use of IsaBGC-AutoExpansion to comprehensive antiSMASH to profile GCFs in 132 *M. luteus* genomes. **b**) Results from the first benchmarking experiment are shown. BGC instances identified by antiSMASH in genomes were mapped to BGC segments identified by running *Isa*BGC-AutoExpansion trained on GCFs from complete *M. luteus* genomes. "NA", shown in black, represent GCF segments which were undetected by antiSMASH. **c**) Results from the first benchmarking experiment are shown. BGC instances identified by *Isa*BGC-AutoExpansion were mapped to BGC segments identified by running *Isa*BGC-AutoExpansion. "NA" , shown in black, represent GCF segments which were undetected by *Isa*BGC-AutoExpansion.

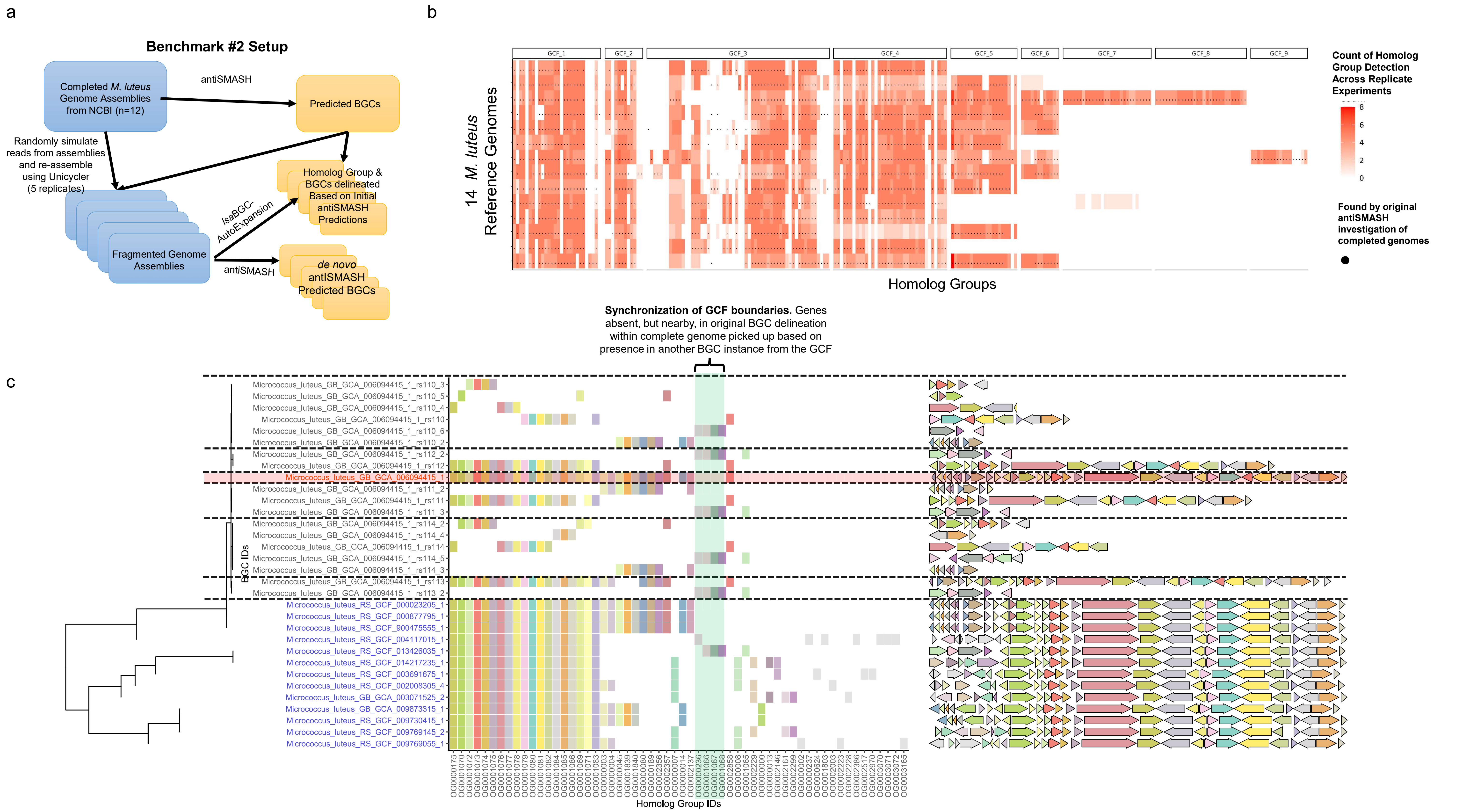

Figure S4: Benchmarking *lsa*BGC-AutoExpansion on simulated fragmented assemblies showcases ability to synchronize GCF boundaries. a) An overview of the second benchmarking experiment is shown comparing the use of *lsa*BGC-AutoExpansion to antiSMASH for predicting BGCs in fragmented versions of 14 complete genomes of *M. luteus*. **b**) The heatmap shows how many times each homolog group from different GCFs were identified by *lsa*BGC-AutoExpansion across 5 replicate simulations where fragmented genomic assemblies were generated from simulated reads for each of the corresponding complete *M. luteus* genomes. Dots signify whether homolog groups were detected as part of a BGC for a particular genome in the original antiSMASH annotation of the completed genome (unfragmented). **c**) *lsa*BGC-See was used to illustrate segments detected for a single GCF, GCF-3, by *lsa*BGC-AutoExpansion in fragmented versions of a single genome, *M. luteus* GCA_006094415, in relation to the BGC detected on the completed genome (unfragmented; highlighted in red) and homologous instances of the GCF in the other 13 completed *M. luteus* genomes (blue labels). Dotted black lines separate GCF instances predicted for the five different replicate fragmented assemblies based on simulated reads from the focal completed genome. The first 6 set of BGC segments illustrates how *lsa*BGC-AutoExpansion is able to find and put together multiple BGC segments into the same GCF despite their dispersion across six different scaffolds. One segment (highlighted in teal) includes genes which were not part of the original BGC delineation on the completed focal genome but were part of homologous BGCs from different completed genomes. We validated these genes are adjacent to the original BGC delineation in the completed focal genome and were simply not included as part of the BGC because they were slightly more distant from core genes/domains of the BGC.

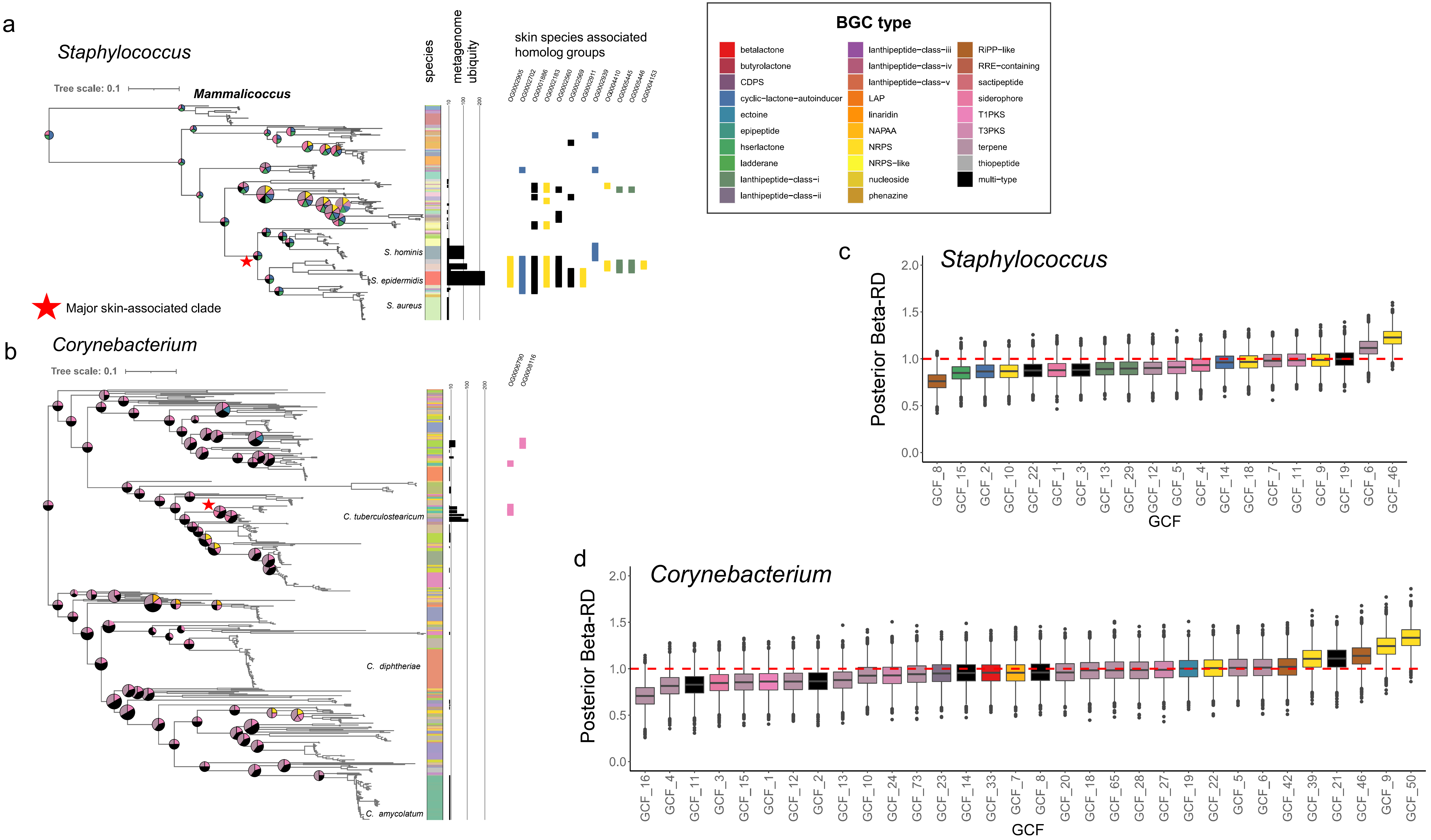

Figure S5: Systematic identification of skin-associated BGC genes and signatures of intra-GCF horizontal gene transfer. A maximum likelihood phylogeny was constructed from ribosomal protein encoding genes for **a**) 229 representative *Staphylococcus* genomes and **b**) 456 representative *Corynebacterium* genomes. Ancestral state reconstruction for GCF carriage was performed using maximum parsimony with the ACCTRAN algorithm and shown as piecharts for innernodes which encapsulate five or more species. Color strips to the right of the phylogenies correspond to the species classification of the genomes. The bar chart to the right of the species color strip corresponds to the number of metagenomes which were found to feature the species. The final track shows the presence of homolog groups found to be enriched in phylogenetic clades where ≥80% of genomes are classified as skin-associated species. lsaBGC-Divergence was used to calculate the Beta-RD statistic between pairs of genomes carrying a particular GCF in representative **c**) *Staphylococcus* and d) *Corynebacterium* genomes. Bayesian shrinking analysis was performed of raw Beta-RD calculates using STAN.

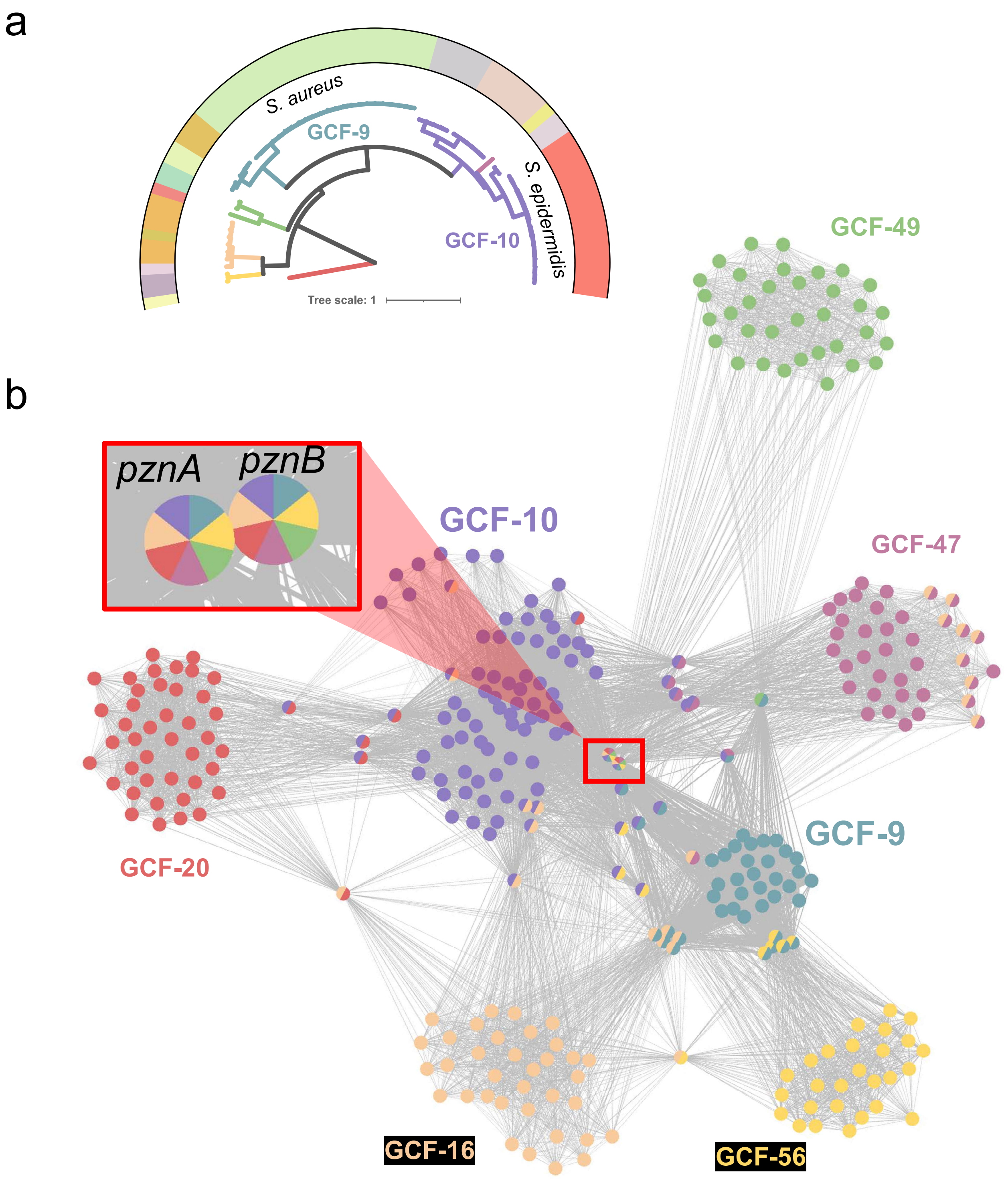

Figure S6: *lsa*BGC expands the known diversity of pyrazinones. a) A maximum-likelihood phylogeny was constructed from PznA and PznB showing the relationship between GCFs encoding for pyrazinones. Branch color corresponds to different GCFs and the outer circular color denotes the species classification for the genome from which the sequences were gathered. **b**) Homolog groups (nodes) across the seven pyrazinone encoding GCFs are depicted as a network where edges indicate that two homolog groups co-occur in the same GCF. The color of each node depicts the distinct pyrazinone GCFs in which the homolog group was found.

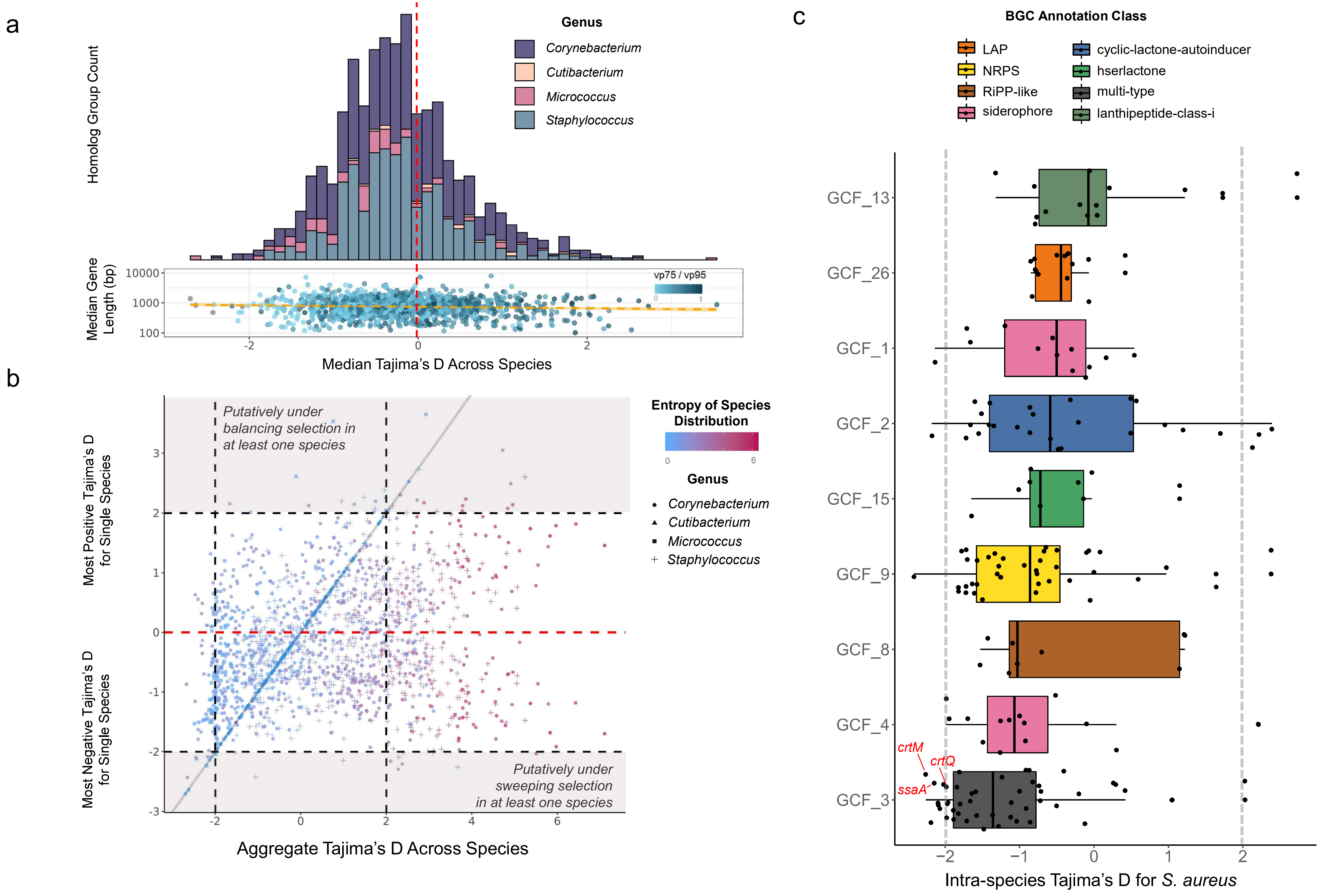

Figure S7: Tajima's D statistic highlights GCFs and homolog groups under selective pressure. a) For each homolog group found in a GCF, Tajima's D statistic was independently calculated per species and the median value across species was determined. The histogram of the median Tajima's D across species exhibits a normal distribution roughly centered around 0. The scatterplot below the histogram showcases that the median Tajima's D across species per homolog group exhibited no correlation with the median length of homolog groups. Each homolog group in the scatterplot is colored according to an analogous statistic to Tajima's D which is the ratio of sites along the homolog group's codon alignment where the major allele is found in $\geq$75% of sequences to sites where the major allele is found in $\geq$95% of sequences. **b**) The aggregate Tajima's D statistic was calculated over all sequences per homolog group and found to be biased by the number of species the homolog group was found in and thus the maximum and minimum Tajima's D per species was investigated instead. **c**) Intra-species Tajima's D calculations using 24 distinct *S. aureus* genomes highlight GCF-3, predicted to encode a hybrid terpene/T3PKS BGC, as feature seven homolog groups with Tajima's D below -2.0, including *crtM*, involved in staphyloxanthin biosynthesis, and *ssaA*, encoding a staphylococcal secretory antigen.

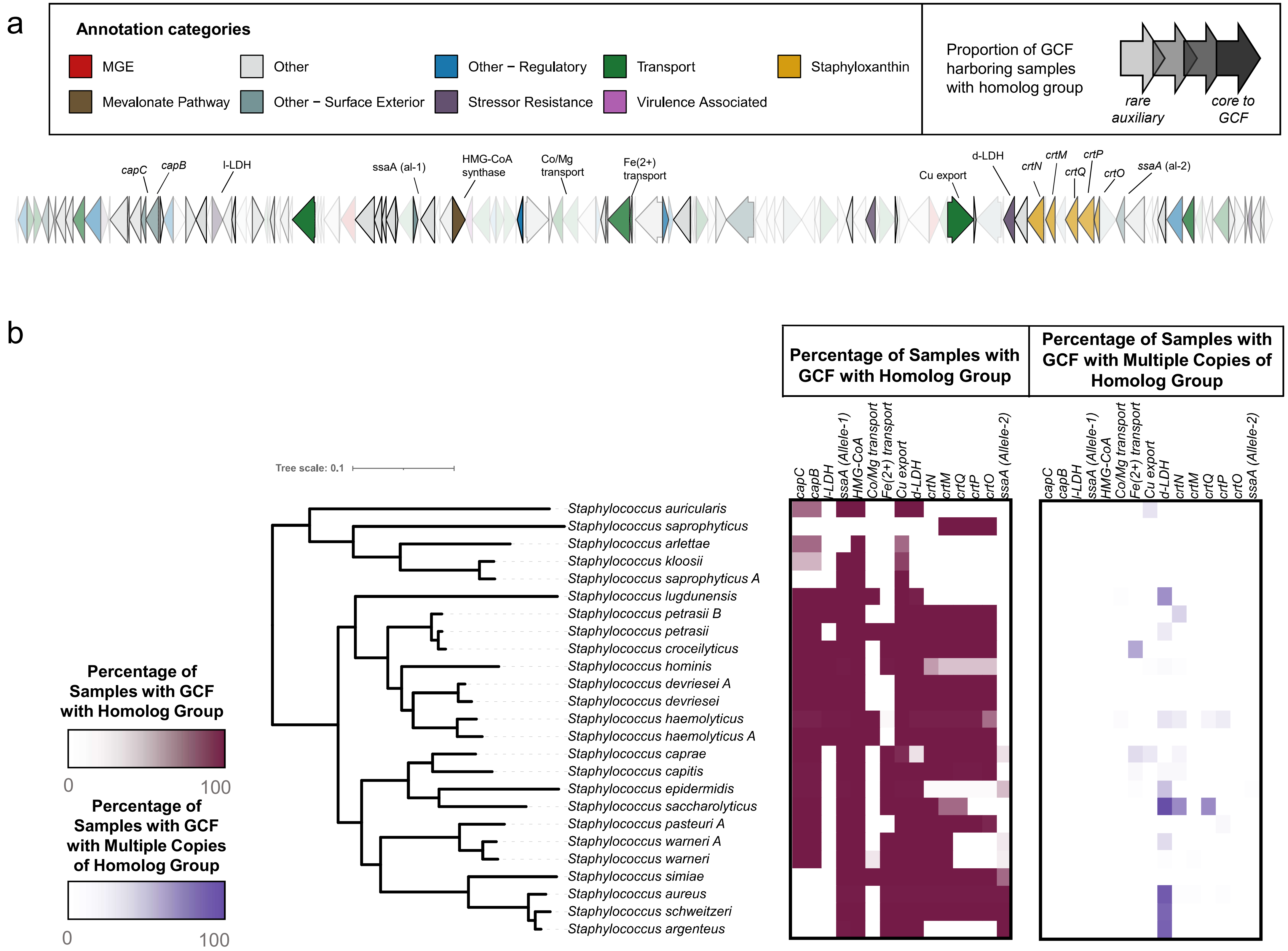

Figure S8: Signatures of conservation and speciation in the surrounding context of staphyloxanthin biosynthesis encoding GCF-3. GCF-3 is a predicted hybrid BGC which includes the staphyloxanthin biosynthesis encoding *crt* operon and the mevalonate pathway related enzyme hydroxymethylglutaryl-CoA synthase, a false positive detection due to homology with type-III polyketide synthases. **a**) A consensus schematic of GCF-3 is shown, generated from individual instances from 103 representative staphylococci found to feature it. Genes are colored according to broad annotation categories and transparency illustrates the proportion of GCF-3 carrying samples found to possess a specific homolog group. **b**) A maximum-likelihood phylogeny for the 25 species within the *S. aureus* / *S. epidermidis* clade found to carry GCF-3 is shown alongside three heatmaps depicting species-specific metrics for select homolog groups. The left heatmap showcases the percentage of the total species genomes found to carry select genes from GCF-3, including the staphyloxanthin encoding *crt* genes. The right heatmap depicts whether homolog groups are found in multiple-copies within the GCF-3 context for different species.

a

b

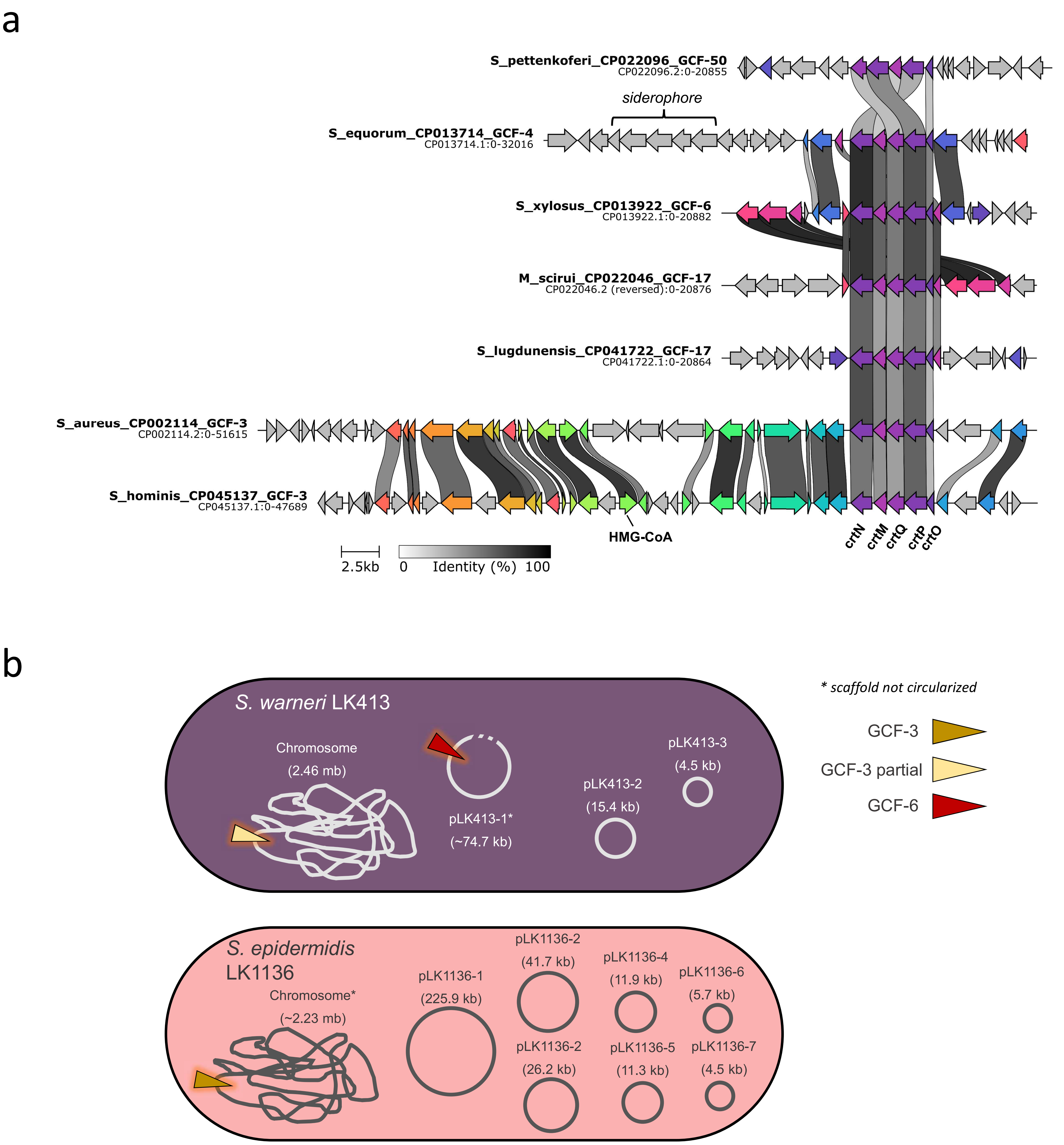

Figure S9: The staphyloxanthin encoding *crt* operon is found in multiple GCFs. a) Sequence and syntenic comparisons between representative instances of GCFs featuring the crt operon encoding for staphyloxanthin were performed and illustrated using clinker. Based on phylogenetic analysis of CrtMN sequences (Figure 3b), the *crt* operon is likely misclassified as GCF-17 for *S. lugdunensis* and should instead be GCF-3. The cause of the misclassification is because the *crt* operon is in a different genomic context within the species as compared to other species in the *S. aureus/epidermidis* clade with GCF-3. **b**) Schematics of the near completed genomic assemblies for *S. epidermidis* LK1136 and *S. warneri* LK413 isolated from skin showcasing the location of staphyloxanthin encoding GCFs.

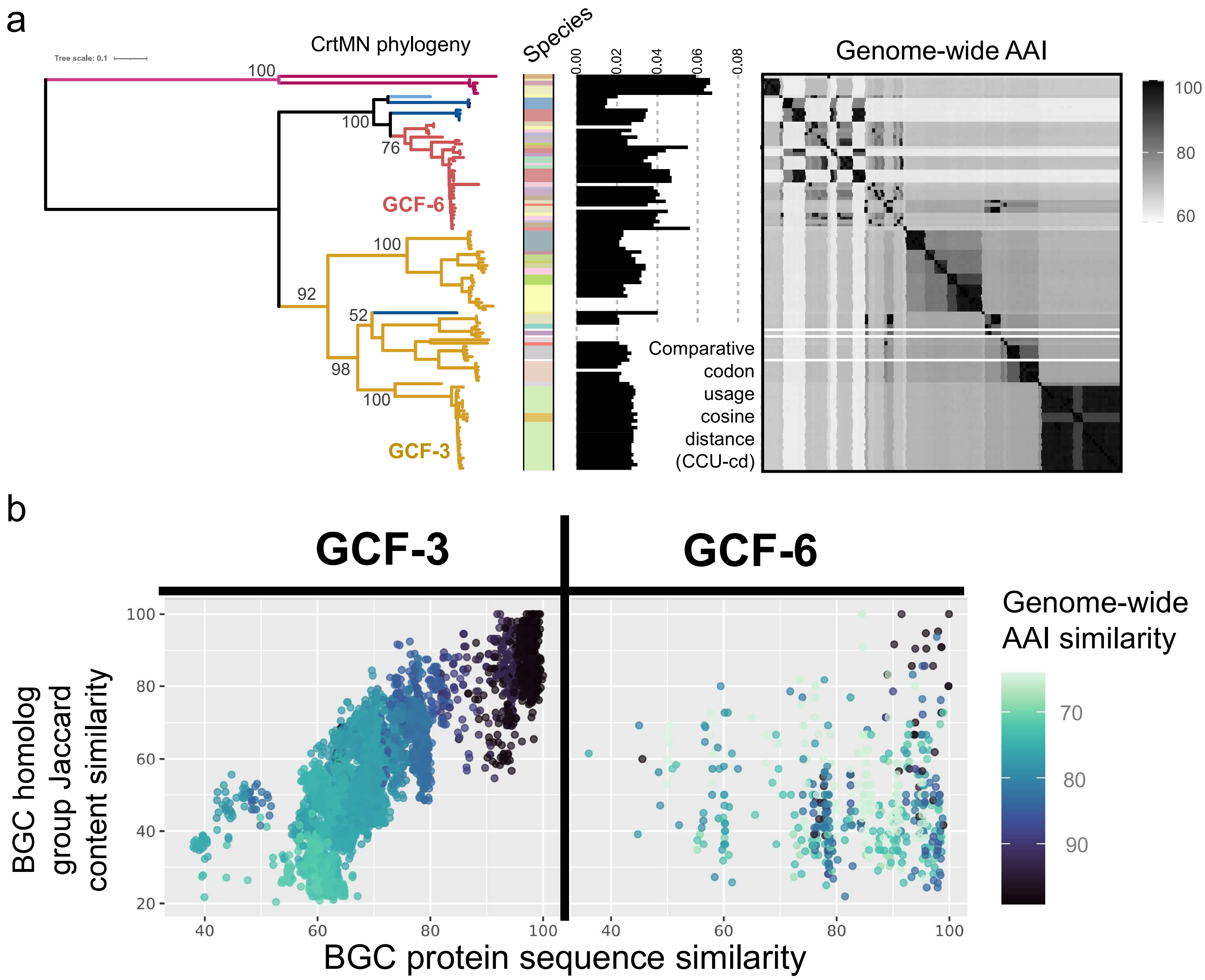

Figure S10: Staphyloxanthin encoding GCF-6 exhibits signatures of mobilization. a) A maximum-likelihood phylogeny was built from CrtM and CrtN protein alignments. Branch colors represent the GCF classification of the CrtMN sequences and bootstrap values are shown for key nodes where GCFs partition. The species the CrtMN sequence was extracted from is shown as a color strip followed by a bar plot depicting the comparative codon usage cosine distance (CCU-cd). CCU-cd was only calculated for the full five gene *crt* operon and represents the codon frequency dissimilarity with the codon frequency of the background genome. The heatmap showcases the genome-wide average amino acid identity (AAI) between pairs of genomes from which CrtMN sequences were gathered. **b**) AAI and shared homolog group content were calculated between pairs of GCF instances from different genomes for GCF-3 and GCF-6 individually. The coloring represents genome-wide AAI similarity.

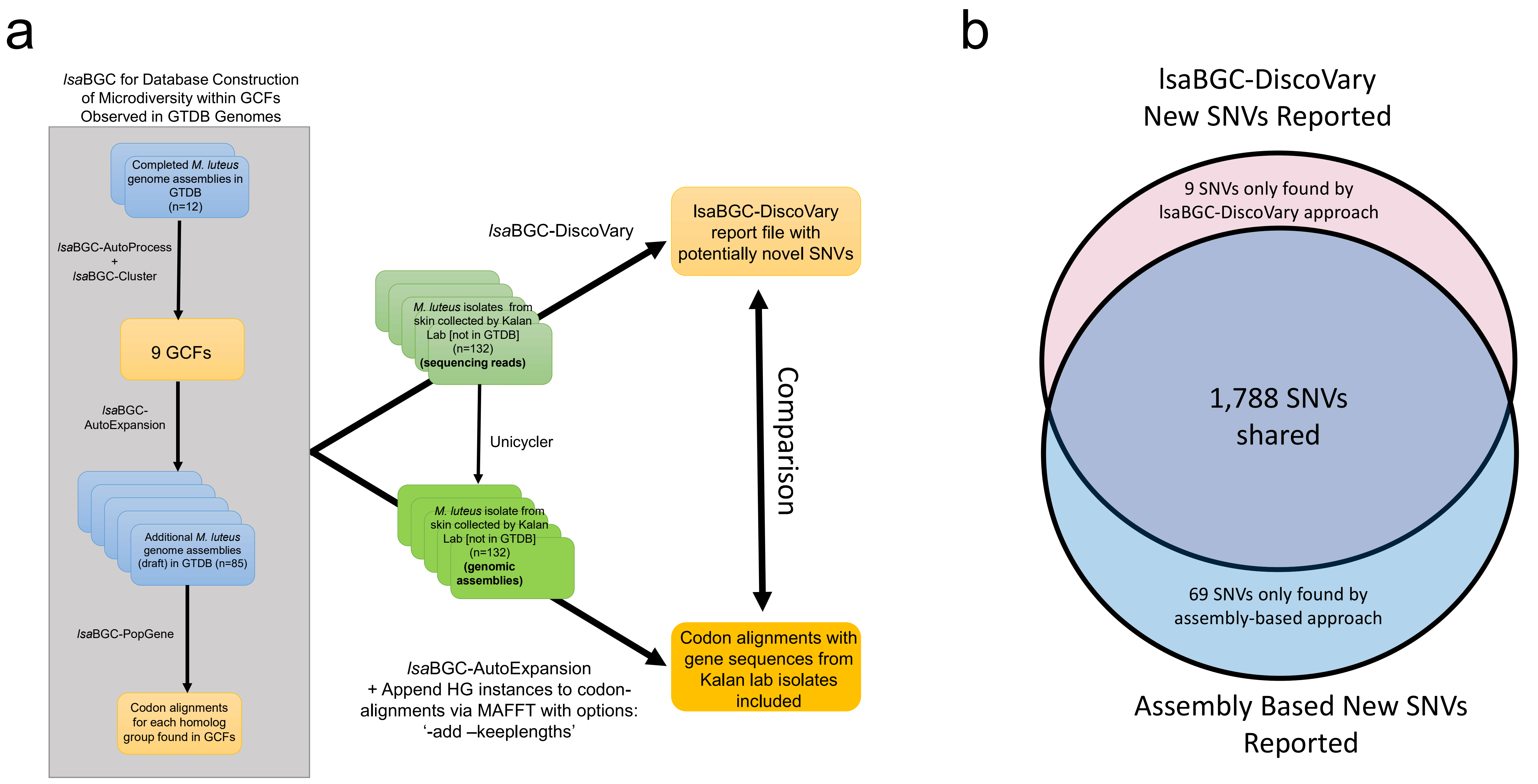

Figure S11: Benchmarking *Isa*BGC-DiscoVary to assembly based SNV identification using single isolate genomic sequencing data for 132 *M. luteus*. a) A schematic of the benchmarking setup for comparing *Isa*BGC-DiscoVary identification of novel SNVs from sequencing reads for 132 *M. luteus* compared to an assembly based identification of novel SNVs for the same isolates. Novel SNVs corresponded to alleles which were not previously represented at specific sites in homolog group codon alignments constructed from publicly available *M. luteus* genomes gathered from NCBI. **b**) A venn diagram showcasing the number of novel SNVs reported by *Isa*BGC-DiscoVary as compared to the novel SNVs found by the assembly-based approach.

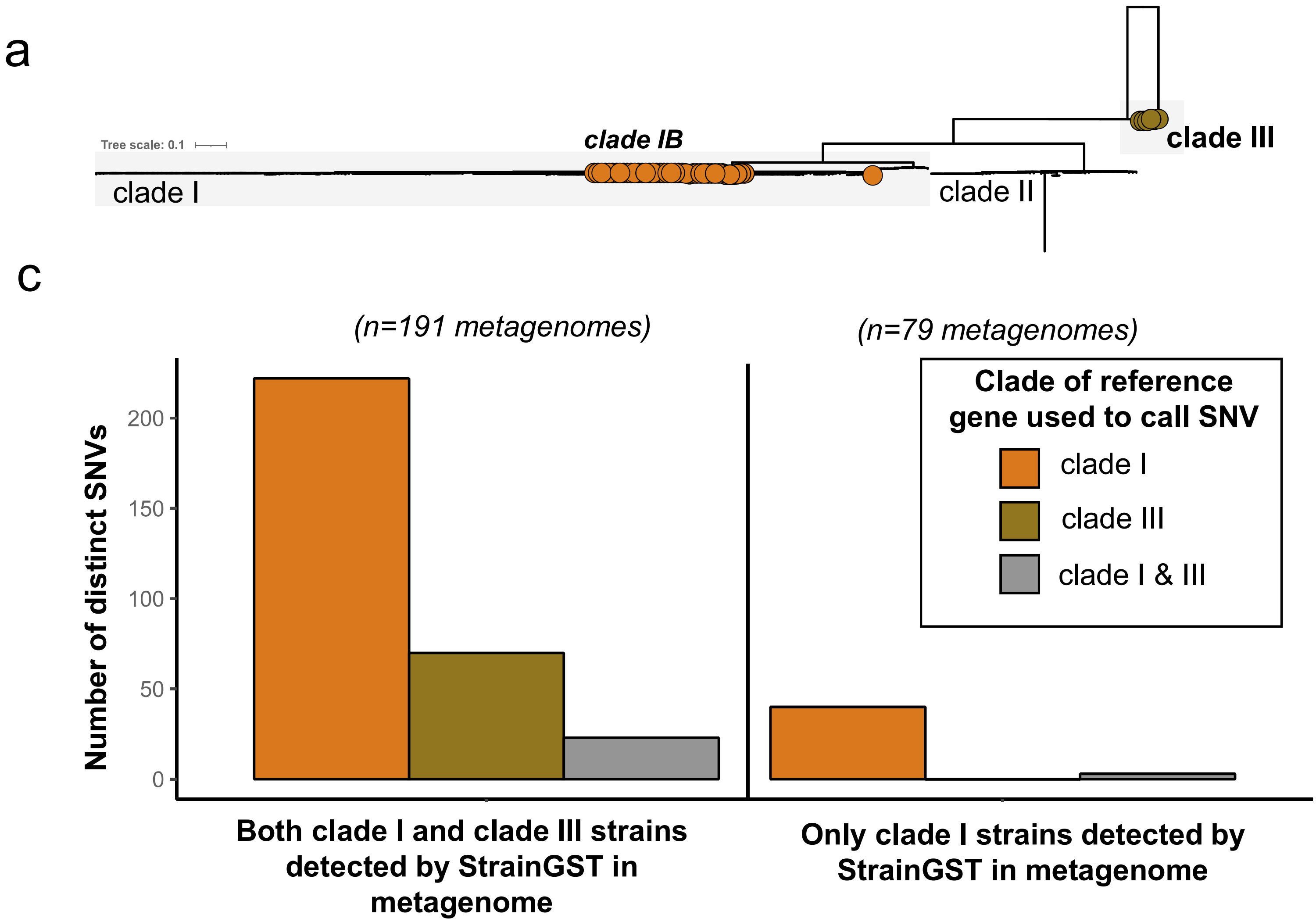

Figure S12: lsaBGC-DiscoVary SNV reporting on BGCs is congruent with whole-genome strain detection tools. a) The cutimycin encoding GCF is found primarily in clade I (subclade IB; orange) and clade III (tan) *C. acnes*. **b**) The mumber of distinct SNVs identified by DiscoVary (post-filtering) which were identified upon reference genes belonging to clade I, clade III, or both clades. Results are shown separately for metagenomes in which StrainGST detected both clade I and clade III strains and metagenomes in which StrainGST only detected clade I strain(s) as present.

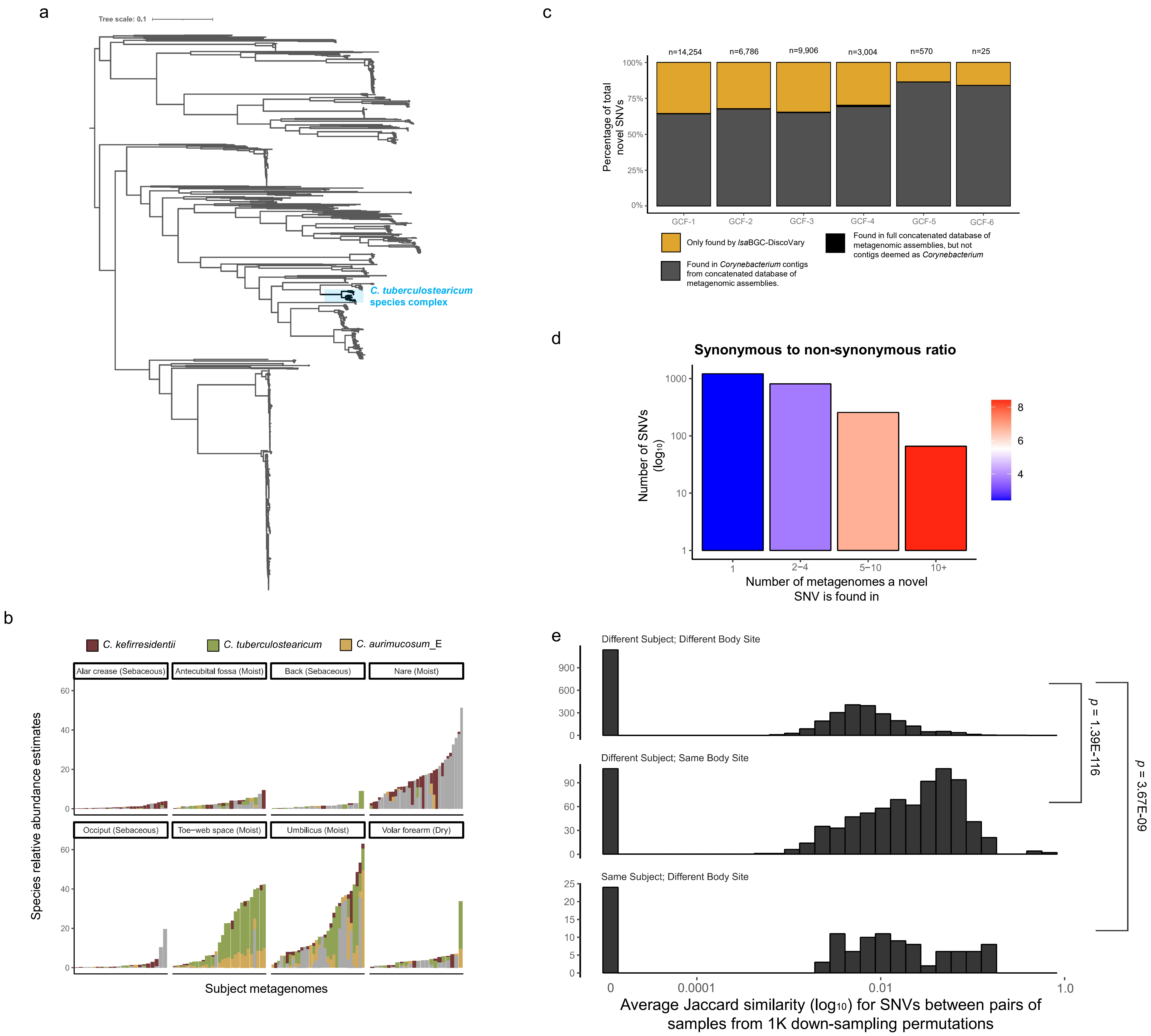

Figure S13: The *C. tuberculostearicum* species complex is highly prevalent across skin metagenomes. a) A more resolute and comprehensive phylogeny of 1,118 *Corynebacterium,* including all 22 genomes found to belong to the *C. tuberculostearicum* species complex in this study, was constructed using a set of 138 genes determined as largely single-copy in Actinomycetota with GToTree. The *C. tuberculostearicum* species complex was found to form a monophyletic clade. **b**) The relative abundance of *Corynebacterium* and representative strains of the *C. tuberculostearicum* species complex in the StrainGST database are shown across skin metagenomes from different body sites and individuals. **c**) Assessment of novel SNVs identified by *Isa*BGC-DiscoVary, post-filtering, for each BGC from the *C. tuberculostearicum* species complex for presence within metagenomic assemblies. **d**) Novel SNVs were tabulated by the number of metagenomes they were observed in. The color of bars corresponds to the ratio of suspected synonymous to non-synonymous SNVs for the sets of novel SNVs. **e**) For metagenomic samples where 30 or more novel SNVs were identified, a multi-iteration, down-sampling based approach was used to compute the average Jaccard similarity for number of shared novel SNVs between pairs of metagenomes. Pairwise comparisons of metagenomes were categorized by whether samples were from the same body-site or subject.

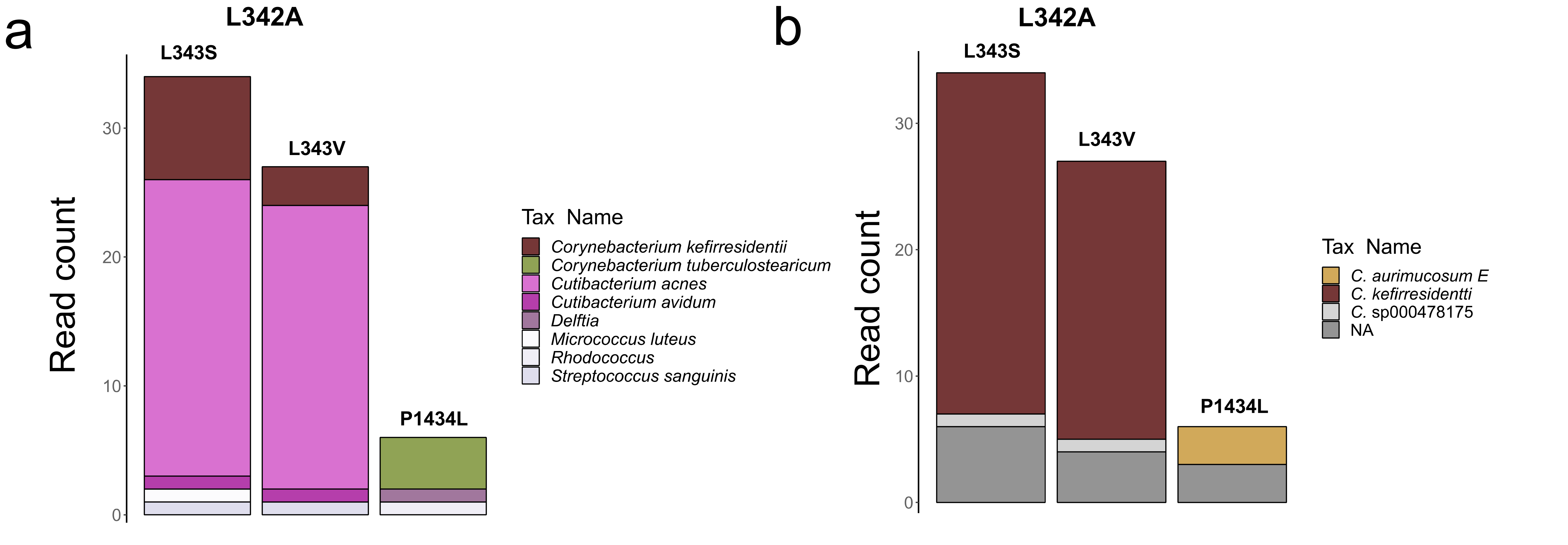

Figure S14: Validation of taxonomic origin for reads supporting the presence of non-synonymous SNVs at highly conserved sites as identified by *lsa*BGC-DiscoVary. a) Reads supporting the existence of three novel SNVs in highly conserved sites of the mycolic acid biosynthesis polyketide synthase (*OG0001691*) were classified taxonomically with Kraken2. Despite many of the reads supporting novel SNV existence being classified as *Cutibacterium*, paired-end alignment to a comprehensive database of all *Cutibacterium* genomes featured in GTDB R202 showed that none aligned concordantly. **b**) Of the reads supporting the existence of novel SNVs, 85.07% aligned as concordant pairs to a comprehensive database of all *Corynebacterium* genomes, with the majority aligning to species belonging to the *C. tuberculostearicum* species complex.
